# Thermal Decomposition and Prebiotic Formation of Adenosine Phosphates in Simulated Early-Earth Evaporative Settings

**DOI:** 10.3390/molecules30173587

**Published:** 2025-09-02

**Authors:** Maheen Gull, Christopher Mehta, Maria Jesus Herrero Perez, Annika Seeley, Karyn L. Rogers, Matthew A. Pasek

**Affiliations:** 1 Department of Earth and Environmental Sciences, Rensselaer Polytechnic Institute, Troy, NY 12180, USA; herrem2@rpi.edu (M.J.H.P.); seelea@rpi.edu (A.S.); rogerk5@rpi.edu (K.L.R.); 2 Rensselaer Astrobiology Research and Education Center (RARE), Rensselaer Polytechnic Institute, Troy, NY 12180, USA; 3 Howard University Plasma Lab (HUPL), Howard University, Washington, DC 20059, USA; chris.mehta@huscience.org

**Keywords:** early earth, phosphorylation, decomposition rates, adenosine phosphates, condensed phosphates, pyrophosphate, nucleotides, origin of life, RNA world

## Abstract

Adenosine nucleotides and polyphosphates play a significant role in biochemistry, from participating in the formation of genetic material to serving as metabolic energy currency. In this study, we examine the stability and decomposition rates of adenosine phosphates—5′-AMP, 5′-ADP, and 5′-ATP (mentioned simply as AMP, ADP and ATP hereafter)—at temperatures of 22–25 °C, 50–55 °C, 70–75 °C, and 85–90 °C, at a pH of 4, over periods of 2 and 4 days, in both saltwater and ultrapure water, under unsealed and completely dried down conditions. We found that adenosine phosphates degrade rapidly under heat and dehydration, particularly at temperatures above 25 °C. Among the three compounds, AMP is the most stable, maintaining its integrity between 22 and 55 °C, whereas ATP begins to degrade at 22–25 °C and ADP is completely decomposed at temperatures above this range. Decomposition rates were analyzed using quantitative ^31^P-NMR, based on the detection of various phosphorus-containing species. AMP primarily hydrolyzed into phosphate, pyrophosphate and even formed 2′,3′-cAMP. In contrast, the condensed adenosine phosphates (ADP and ATP) hydrolyzed to AMP, phosphate, pyrophosphate, triphosphate, 5′-AMP and, in some cases, 2′,3′-cyclic adenosine monophosphate (2′,3′-cAMP). We also investigated the formation of these compounds in the presence of N-containing additives such as thiourea, urea, imidazole, and cyanamide at temperatures between 65 and 70 °C. Among these, cyanamide and urea were particularly effective in promoting the synthesis of adenosine monophosphates (AMPs) from phosphate and adenosine. The major products observed were 2′,3′,5′-AMPs and cyclic 2′,3′-AMPs. In some experiments, adenosine diphosphate (ADP) and dimeric nucleotide species were also detected. These findings suggest that moderately heated evaporating pools could facilitate the abiotic formation of AMPs. However, such environments would likely have been unsuitable for the long-term accumulation of these compounds due to continued degradation, though there would exist some level of these nucleotides at steady state.

## 1. Introduction

Adenosine phosphates—AMP (adenosine monophosphate), ADP (adenosine diphosphate), and ATP (adenosine triphosphate)—are central to both modern biology and theories regarding the origin of life. These molecules play crucial roles in the biochemistry of living organisms. ATP functions as the primary energy currency of the cell, powering nearly all cellular processes by transferring its terminal phosphate group to other molecules through phosphorylation. ADP and AMP also participate in this energy cycling system, with the reversible interconversion between ATP ⇌ ADP ⇌ AMP forming a fundamental basis of metabolism.

Beyond their role in energy transfer, adenosine phosphates are involved in metabolic pathways such as glycolysis, cellular respiration, and photosynthesis. They also participate in signal transduction pathways; for example, cyclic AMP (cAMP), a derivative of AMP, acts as a key messenger in many signaling cascades [1,2]. Moreover, AMP is one of the four nucleotide monomers that make up RNA. Thus, adenosine phosphates serve not only as energy carriers but also as informational molecules, linking metabolism to genetic information [3,4].

It is widely believed that an RNA world preceded the emergence of DNA-based life [5,6], and that the components necessary to form RNA may have been present in prebiotic environments, where they could undergo chemical reactions leading to the synthesis of replicating and evolving polynucleotides [6,7]. The chemical structure of an RNA nucleotide consists of three molecular subunits: a phosphate group, a ribose sugar, and a nitrogenous base. It is hypothesized that these subunits were formed independently on the early Earth via distinct prebiotic pathways [8,9,10,11,12,13,14] and were subsequently joined through dehydration-condensation reactions [9]. The formation of both the phosphate–ribose and ribose–nucleobase linkages involves dehydration and is thermodynamically endergonic [15] in water.

The energy required to phosphorylate organic molecules is approximately 15 kJ/mol and typically needs either very low water activity or the presence of reactive inorganic phosphorus compounds [15]. Such conditions—characterized by low water activity—were likely found on early Earth in arid regions or in drying, warm ponds, as originally suggested by Darwin [16,17], where phosphorylation reactions could have been promoted [18,19]. Evaporating (and possibly warm or hot) small bodies of water—such as rain puddles, geothermal ponds, and tidal pools—would have provided ideal environments for the formation and concentration of nucleotides. These scenarios appear both realistic for early Earth and thermodynamically favorable [5].

The formation of phosphate diesters from monoesters may have enabled the synthesis of nucleic acids, potentially opening a pathway to the RNA world. Therefore, understanding the prebiotic steady state occurrence of simple nucleotides is essential to understanding the origin of RNA-based life. Significant progress has been made in elucidating the prebiotic phosphorylation of nucleosides to form nucleotides. High-yield syntheses of adenosine phosphates have been achieved under a variety of conditions, including the following: using Na_2_HPO_4_ or hydroxylapatite [Ca_5_(PO_4_)_3_OH] at 60–100 °C, often with additives such as NH_4_Cl, urea, or NH_4_HCO_3_ [20]; using cyclic trimetaphosphate (cyclic TMP) with Ni^2^⁺ at 70–85 °C [21]; using condensed phosphate compounds such as Graham’s salt, Na_4_P_2_O_7_, or Na_5_P_3_O_10_, under reflux at 100 °C for 4–6 h at high pH [22]; under prebiotically relevant solvents like formamide, acetamide, N-methylformamide, N-methylacetamide, and N,N-dimethylformamide using NH_4_H_2_PO_4_ as a P source [23]; using various orthophosphate minerals (Herderite: Ca(BePO_4_F), Hureaulite: Mn^2+^_5_(PO_3_(OH)_2_(PO_4_)_2_(H_2_O)_4_, Libethenite: Cu^2+^_2_(PO_4_)(OH), Pyromorphite: Pb_5_(PO_4_)_3_Cl, Turquoise: Cu^2+^Al_6_(PO_4_)_4_(OH)_8_(H2O)_4_, Fluorapatite: Ca_5_(PO_4_)_3_F, Hydroxylapatite Ca_5_(PO_4_)_3_OH, Vivianite Fe^2+^_3_(PO_4_)_2_(H_2_O)_8_, Cornetite Cu^2+^_3_(PO_4_)(OH)_3_, Pseudomalachite Cu^2+^_5_(PO_4_)_2_(OH)_4_, Reichenbachite Cu^2+^_5_(PO_4_)_2_(OH)_4_, and Ludjibaite Cu^2+^_5_(PO_4_)_2_(OH)_4_) [24], ammonium or alkali dihydrogen phosphates [25] and with different orthophosphate, hydrogen phosphate or dihydrogen phosphate salts or with different condensed phosphate salts [26] with formamide as a solvent medium [24,25,26]; using activated phosphate species, including imidazolidine-4-thione phosphates [27] and activated imidazole phosphates [28]; in the presence of borates, which may have stabilized ribose and facilitated phosphorylation [29,30,31]; using deep eutectic solvents (DESs) [32]; using meteoritic mineral schreibersite or its synthetic analog (Fe_3_P) [33]; through Fenton-type chemistry, using hypophosphite at high pH in the presence of urea [34]; using diamidophosphate (DAP) as a phosphorylation agent [35,36]; in the presence of lunar soil simulants using NaH_2_PO_4_ [37]. These diverse experimental systems suggest that early Earth chemistry likely involved a combination of multiple scenarios, each contributing to nucleoside phosphorylation and accumulation under prebiotic conditions [38].

Furthermore, to understand the origin of biopolymers—and life itself—it is crucial to investigate the stability of these compounds under early Earth conditions. The decomposition rates of adenosine phosphates (5′-AMP, ADP, and ATP) have been previously reported [39]. That study examined the effects of temperature and pH in both distilled water and synthetic seawater, the latter used to simulate a primitive ocean environment. Hydrolysis rates were calculated by monitoring the release of inorganic orthophosphate [39]. The results indicated that ATP had the highest rate of hydrolysis, followed by ADP, whereas AMP was the most stable and resistant to hydrolysis under the experimental conditions. This study particularly represented a simulated early Earth ocean.

Recently, the hydrolysis rates of ATP in aqueous solutions at pH 3 and 7, and at temperatures of 80, 100, and 120 °C, have been studied using Raman spectroscopy. The rate constants at 120 °C were found to be 4.34 × 10^−3^ s^−1^ at pH 3 and 2.91 × 10^−3^ s^−1^ at pH 7, corresponding to ATP half-lives of just a few minutes. Notably, these experiments were conducted under hydrothermal conditions [40]. Despite the different focuses of these research studies, neither specifically examined the full range of phosphorus-containing decomposition products beyond orthophosphate [39,40].

Furthermore, while mildly hot, evaporating pools have been proposed as plausible prebiotic environments and have shown great potential in abiotic synthesis [17,18,19,41,42,43,44], the decomposition rates of AMP, ADP, and ATP under such conditions have not been previously explored. Therefore, the stability and degradation pathways of adenosine phosphates under prebiotic, evaporative conditions remain an open question.

In the present study, we report the decomposition rates of AMP, ADP, and ATP under simulated early Earth conditions representing a drying, evaporating body of water, an environment often invoked for the synthesis of these compounds. Experiments were conducted at various temperature ranges—22–25 °C, 50–55 °C, 70–75 °C, and 85–90 °C—in both salt solutions and ultrapure water, over periods of 2 and 4 days, at a constant pH of 4. We observed the behavior of these adenosine phosphates and specifically analyzed the phosphorus-containing products in each sample using ^31^P-NMR spectroscopy. Additionally, to complement the decomposition studies, we report the prebiotic synthesis of these compounds under the same conditions. This two-pronged approach provides insights into both the formation rates of organophosphates and their relative steady state abundance under drying, evaporative pool scenarios on the early Earth.

## 2. Results

### 2.1. ^31^P-NMR Analyses of Adenosine Phosphates

Decomposition reactions of AMP, ADP, and ATP were studied by monitoring the production of phosphorus-containing species from the starting material using ^31^P-NMR spectroscopy (Table 1) (also see Supplementary Information, Figures S1–S52) [45,46]. ^31^P-NMR of starting compounds, e.g., AMP, ADP and ATP, did not show any additional P products as impurities (Supplementary Information, Figures S1–S3). The analysis focused exclusively on phosphorus-based decomposition products; decomposition of the nucleoside portion (e.g., into nucleobases, ribose, or other thermally derived heterocycles such as deamination) was not examined.

#### 2.1.1. AMP

**Scheme 1:**
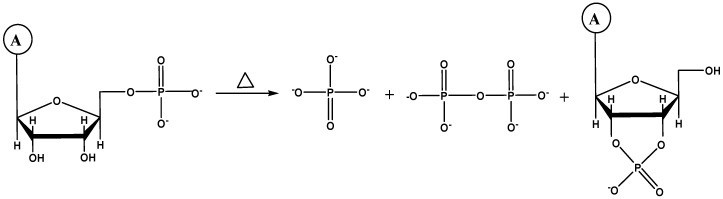
Major decomposition P products of the thermal heating reactions of AMP. The major P products (as detected by ^31^P-NMR) include orthophosphate, pyrophosphate and 2′,3′-cAMP.

For AMP, the primary decomposition products identified by ^31^P-NMR were inorganic phosphate (Pi) and pyrophosphate (PPi), as illustrated in Scheme 1. At 22–25 °C, AMP exhibited no hydrolysis in both ultrapure water and salt (Instant Ocean) solution. No phosphorus-containing degradation products were detected under these conditions, and only intact 5′-AMP was observed. This stability pattern was consistent across both 2-day and 4-day reaction durations.

At the temperature range of 50–55 °C, heating AMP in ultrapure water for 2 days resulted in no observable hydrolysis. However, when the same conditions were extended to 4 days, AMP partially decomposed to inorganic phosphate (Pi) and pyrophosphate (PPi).

In contrast, AMP heated in saltwater (Instant Ocean) at 50–55 °C for 4 days experienced more hydrolysis compared to pure water (Table 1). When AMP was heated for 4 days in the saltwater at the same temperature range (50–55 °C), the predominant decomposition product was inorganic phosphate as opposed to the formation of inorganic condensed P compounds (likely due to divalent cations present in the saltwater that usually ties up P as phosphate).

At the temperature range of 70–75 °C, AMP hydrolyzed in ultrapure (or DDI) water after 2 days of heating to both pyrophosphate (PPi) and inorganic phosphate (Pi). Continued heating for 4 days led to further decomposition to both phosphate species. In this temperature window, we also observed some (5–7%) 2′,3′-cyclic adenosine monophosphate (2′,3′-cAMP) when the reaction was heated for 4 days in DDI.

**Figure 1:**
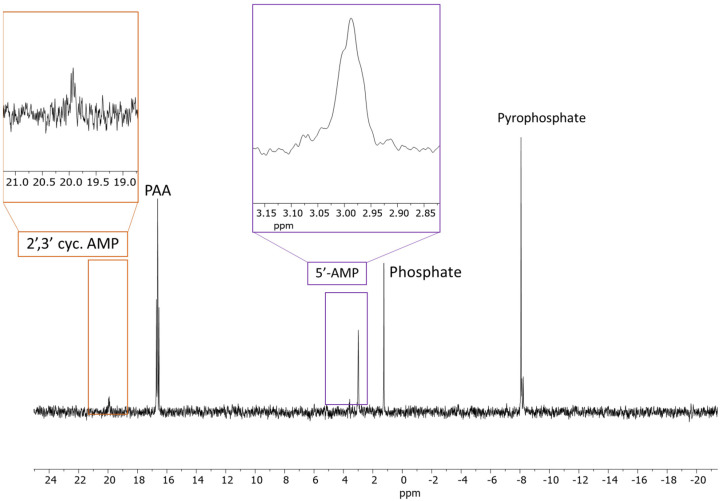
Thermal decomposition reactions of AMP at 70–75 °C for 4 days: H-coupled ^31^P-NMR spectrum of products formed by heating an unsealed aqueous solution (pH = 4) (ultrapure water) of AMP at 70–75 °C for 4 days until dry (Reaction Sample AD-7). The peaks appear, from right to left, in the following order: pyrophosphate, phosphate, AMP, PAA (phosphonoacetic acid, internal standard), and 2′,3′-cAMP. Internal standard was used as a reference standard and for quantification purposes.

**Figure 2:**
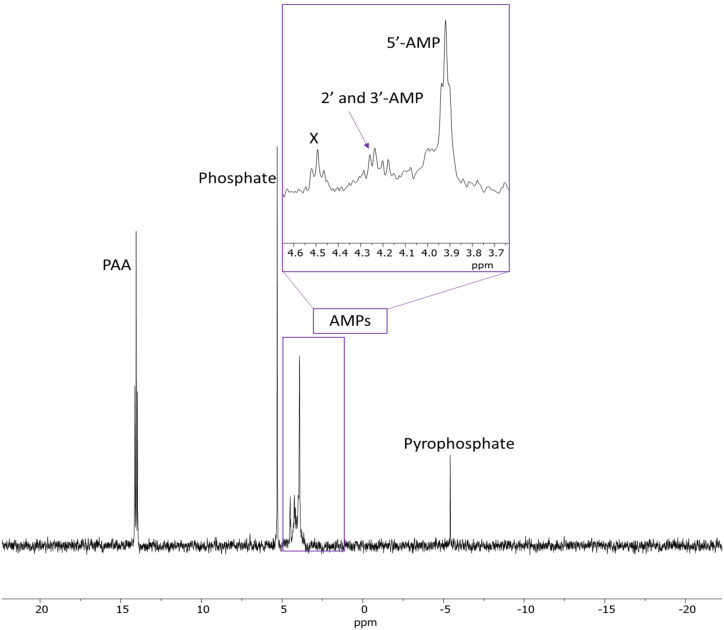
Thermal decomposition reactions of AMP at 70–75 °C for 2 days: H-coupled ^31^P-NMR spectrum of phosphorus-containing decomposition products formed by heating an unsealed aqueous solution (pH = 4) (in saltwater) of AMP at 70–75 °C for 2 days until dry (Reaction Sample AD-11). The peaks appear, from right to left, in the following order: pyrophosphate, phosphate, AMP, 2, and 3′-AMP and an unknown triplet (X) at 4.5 ppm, PAA (phosphonoacetic acid, internal standard). However, only in Sample No AD-10, AD-11 and AD-15, some unknown peaks around 3.5–4.5 ppm were observed, quite possibly be the 2′ and 3′-AMP or AMP diphosphate (not pyrophosphate) species.

In the reaction sample containing saltwater solution (at 70–75 °C, 2 days), various adenosine diphosphate species (not as pyrophosphates but individual phosphate groups attached to two of the 2′, 3′ and/or 5′ positions, respectively). Overall, the formation of the 2′,3′-cAMP was observed only in samples heated at the temperature range of 70–75 °C. The formation of this cyclic adenosine phosphate appeared to be promoted by prolonged heating and the presence of saltwater (Figure 1 and Figure 2).

In saltwater, the extent of hydrolysis continued to increase with increasing temperature and increasing heating time spans. Furthermore, across all solution environments, a greater accumulation of pyrophosphate was consistently observed in samples heated for 4 days compared to those heated for 2 days.

Interestingly, the formation of 2′,3′-cAMP was also observed, indicating intramolecular cyclization of AMP. Although no clear correlation was found between the formation of this cyclic compound and a specific temperature window, it was detected only in samples heated for 4 days. This suggests that prolonged heating favors cyclization, potentially due to extended exposure to thermal energy facilitating intramolecular condensation.

#### 2.1.2. ADP

**Scheme 2:**
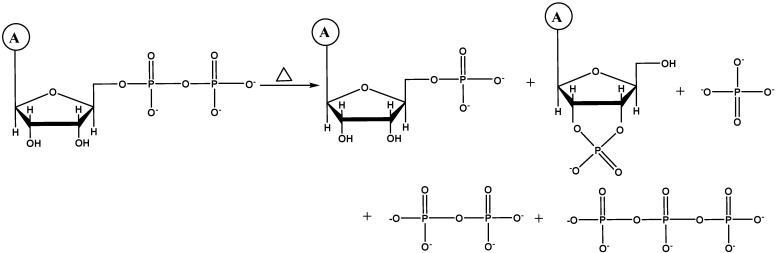
Major decomposition P products of the thermal heating reactions of ADP. The major P products (as detected by ^31^P-NMR) include orthophosphate, pyrophosphate, AMP, triphosphate and 2′,3′-cAMP.

In the case of ADP, the major decomposition products were inorganic phosphate (Pi), pyrophosphate (PPi), 5′-AMP, 2′,3′-cAMP, and inorganic triphosphate (Scheme 2). At the temperature range of 22–25 °C, ADP was stable. At temperatures of 50–55 °C and above, ADP was no longer detected in any of the reactions. This indicates that ADP is unstable at higher temperatures and rapidly hydrolyzes even after a few days of heating to dryness at moderately elevated temperatures (Table 2, Scheme 2).

#### 2.1.3. ATP

**Scheme 3:**
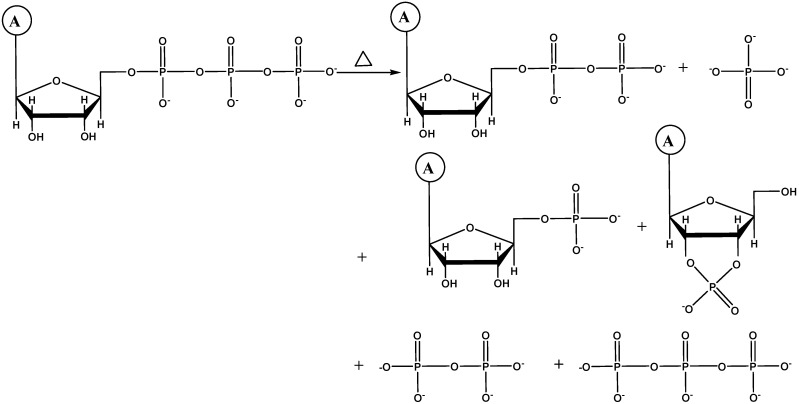
Major decomposition P products of the thermal heating reactions of ATP. The major P products (as detected by ^31^P-NMR) include orthophosphate, pyrophosphate, ADP, AMP, triphosphate and 2′,3′-cAMP.

In the case of ATP, the primary decomposition products identified by ^31^P-NMR were inorganic phosphate (Pi), pyrophosphate (PPi), 5′-AMP, ADP, 2′,3′-cAMP, and inorganic triphosphate (Scheme 3). Interestingly, ATP remained detectable in all reaction mixtures heated in ultrapure or DDI water for 2 days across the temperature range of 22–55 °C. At the higher temperature range (70–90 °C), it was no longer observed, indicating significant hydrolysis. In contrast, when ATP was heated under saltwater conditions, it mostly hydrolyzed at all temperatures greater than 22–25 °C. This suggests that ATP is considerably less stable in saltwater environments, even at moderately elevated temperatures (Table 3, Scheme 3).

**Figure 3:**
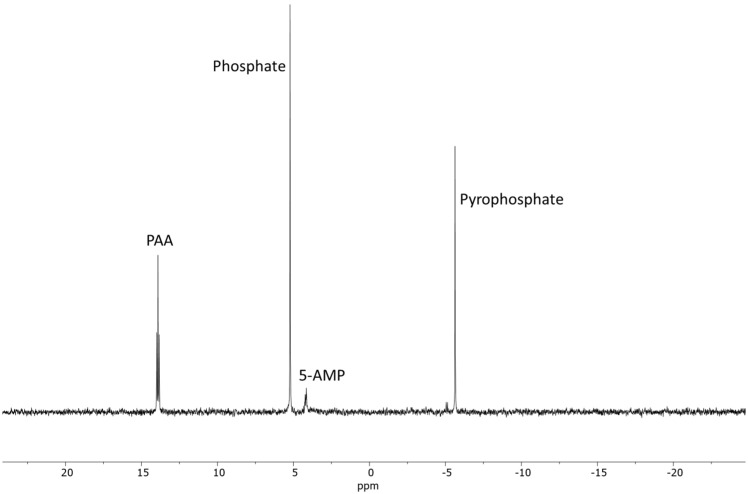
Thermal decomposition reactions of ADP at 50–55 °C for 4 days (in DDI): H-coupled ^31^P-NMR spectrum of phosphorus-containing decomposition products formed by heating an unsealed aqueous solution (pH = 4) (DDI water) of ADP at 50–55 °C for 4 days until dry (Reaction Sample AD-22). The peaks appear, from right to left, in the following order: pyrophosphate, phosphate, AMP, 2′, and 3′-AMP, PAA (phosphonoacetic acid, internal standard).

**Figure 4:**
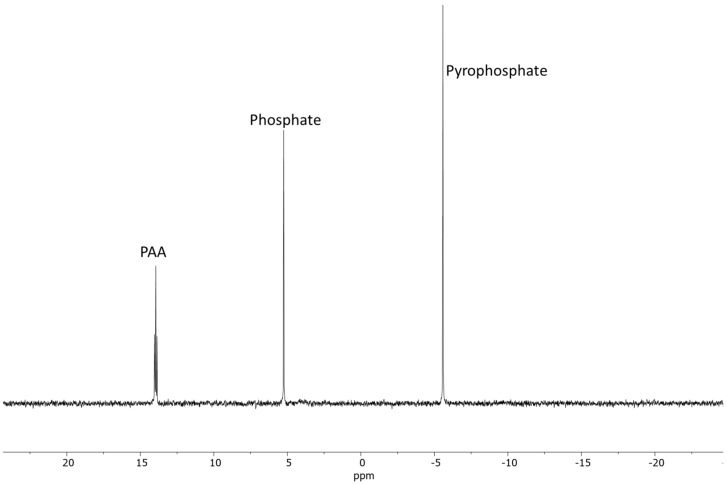
Thermal decomposition reactions of ADP at 50–55 °C for 4 days (in saltwater): H-coupled ^31^P-NMR spectrum of phosphorus-containing decomposition products formed by heating an unsealed aqueous solution (pH = 4) (saltwater) of ADP at 50–55 °C for 4 days until dry (Reaction Sample AD-30). The peaks appear, from right to left, in the following order: pyrophosphate, phosphate and PAA (phosphonoacetic acid, internal standard).

**Figure 5:**
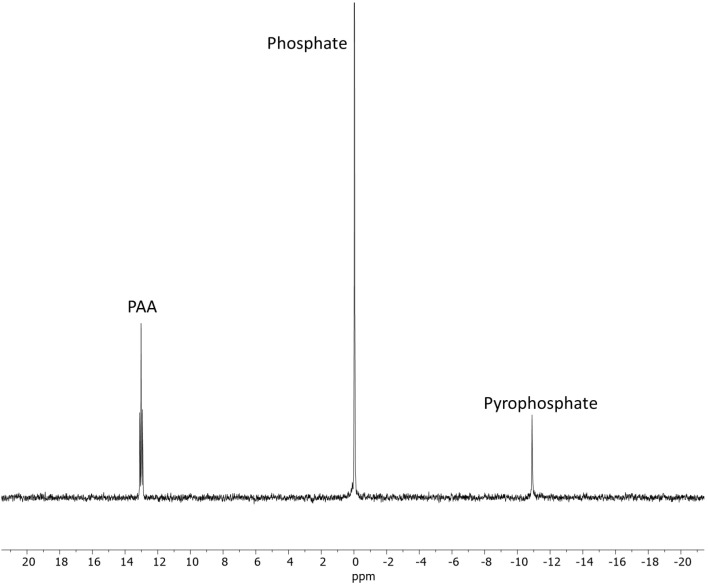
Thermal decomposition reactions of ATP at 85–90 °C (in DDI) for 2 days: H-coupled ^31^P-NMR spectrum of phosphorus-containing decomposition products formed by heating an unsealed aqueous solution (pH = 4) (DDI water) of ATP at 85–90 °C for 2 days until dry (Reaction Sample AD-36). The peaks appear, from right to left, in the following order: pyrophosphate, phosphate and PAA (phosphonoacetic acid, internal standard).

**Figure 6:**
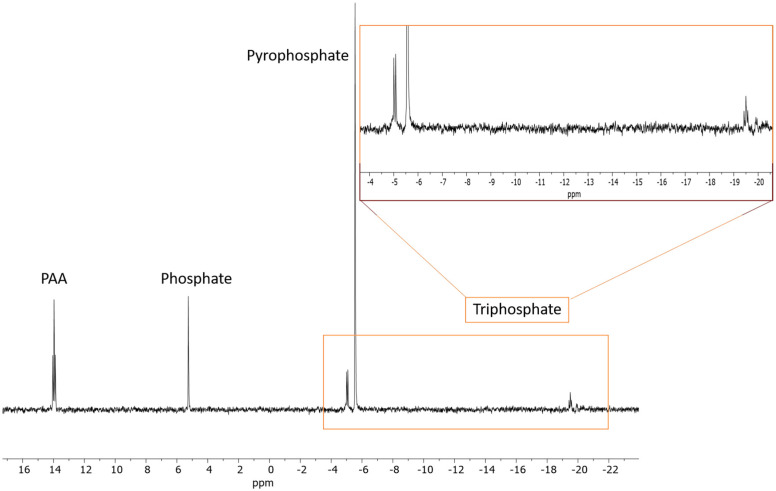
Thermal decomposition reactions of ATP at 85–90 °C (in saltwater) for 2 days: H-coupled ^31^P-NMR spectrum of phosphorus-containing decomposition products formed by heating an unsealed aqueous solution (pH = 4) (saltwater) of ATP at 85–90 °C for 2 days until dry (Reaction Sample AD-44). The peaks appear, from right to left, in the following order: triphosphate, pyrophosphate, phosphate and PAA (phosphonoacetic acid, internal standard).

In our experiments, both ADP and ATP underwent rapid hydrolysis under most conditions. For ADP, detectable amounts were observed only in samples stirred at 22–25 °C, while it was absent from the majority of other reaction samples (Figure 3 and Figure 4), highlighting its pronounced thermal lability. By contrast, ATP exhibited greater stability in ultrapure (DDI) water when heated at 22–55 °C for up to two days (Figure 5 and Figure 6). However, the presence of salts appeared to slightly decrease its stability. As expected, higher temperatures and extended heating times were generally detrimental to the integrity of all adenosine phosphates.

#### 2.1.4. Comparison of Various P Products in Decomposition Reactions of AMP, ADP and ATP

**Figure 7:**
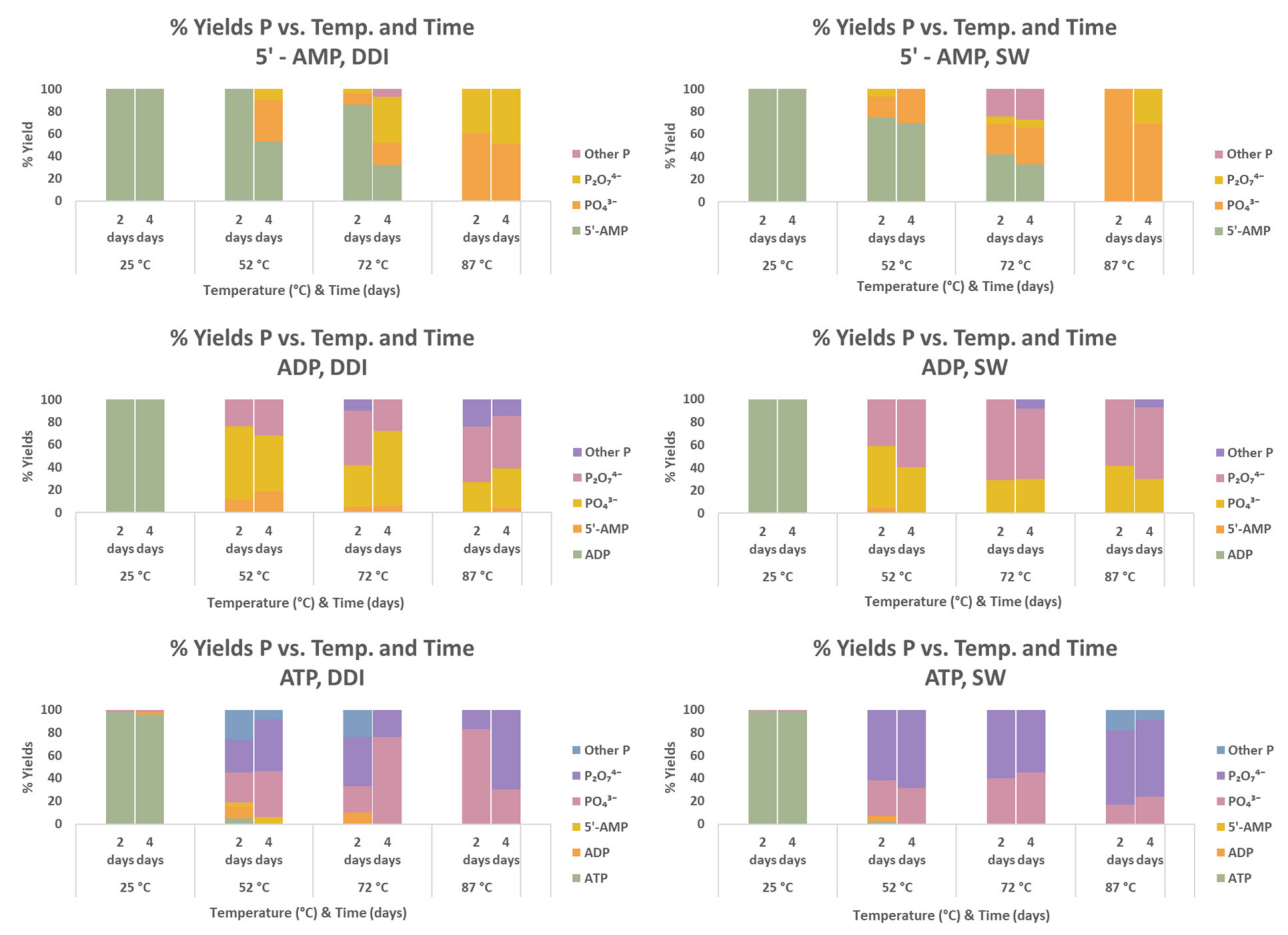
Comparison of yields (%) of various adenosine phosphates and inorganic phosphorus (P) species across different temperature ranges (22 °C–90 °C) over 2- and 4-day periods at pH = 4. The reactions were conducted in both ultrapure (doubly deionized, DDI) water and saltwater (SW). Phosphorus-containing species were identified using ^31^P-NMR spectroscopy, based on their chemical shift values and by comparison of proton-coupled and decoupled spectra. Here, DDI refers to doubly deionized (ultrapure) water, and SW refers to saltwater. The category of “other P species” includes the following: AMP reactions: primarily 2′,3′-cAMP, and in some cases, 2′-AMP, 3′-AMP, and diphosphate derivatives (not pyrophosphate), in which two separate orthophosphate groups are linked to the 2′ or 3′ positions as well as the 5′ position of the nucleoside, for ADP and ATP, primarily inorganic triphosphate.

We observed different P compounds both organophosphorus and inorganic compounds in our hydrolysis studies (Figure 1, Figure 2, Figure 3, Figure 4, Figure 5, Figure 6 and Figure 7). In case of AMP, phosphate and pyrophosphate were the major inorganic P species, while 2′,3′-cAMP, 2′ and 3′, as well as adenosine diphosphates (see prior note) were also identified. In case of ADP hydrolysis, inorganic P species included phosphate, pyrophosphate and triphosphate, and AMP was the major organophosphorus compound observed in these sets of experiments. For ATP, the major inorganic P products of hydrolysis were similar to that of ADP, while organophosphorus compounds included both ADP and AMP (see Supplementary Information, Figure S52a–l).

### 2.2. Prebiotic Synthesis Reactions of Adenosine Phosphates

In addition to studying the decomposition rates of adenosine phosphates at elevated temperatures, we were also interested in evaluating their formation under similar conditions. Based on previously reported optimal conditions [15,18,19,36,46], we selected a temperature range of 65–70 °C for our phosphorylation experiments (Supplementary Information, Figures S53 and S54).

**Figure 8:**
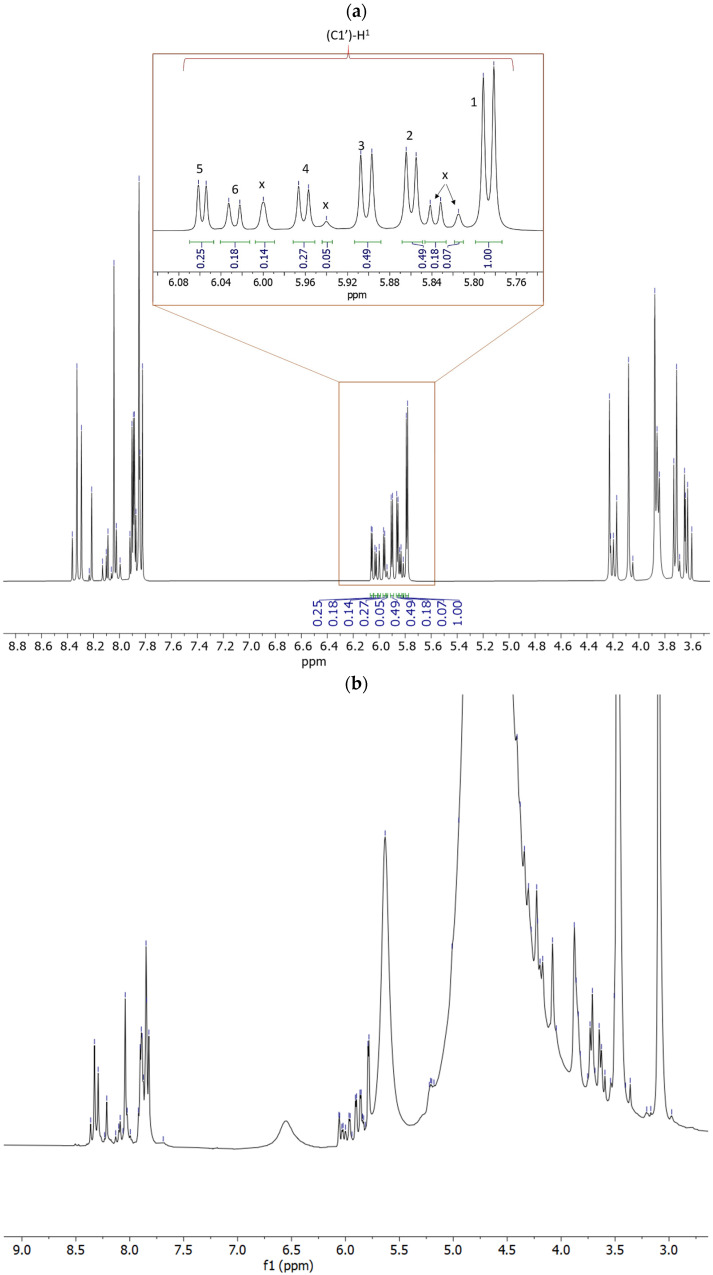

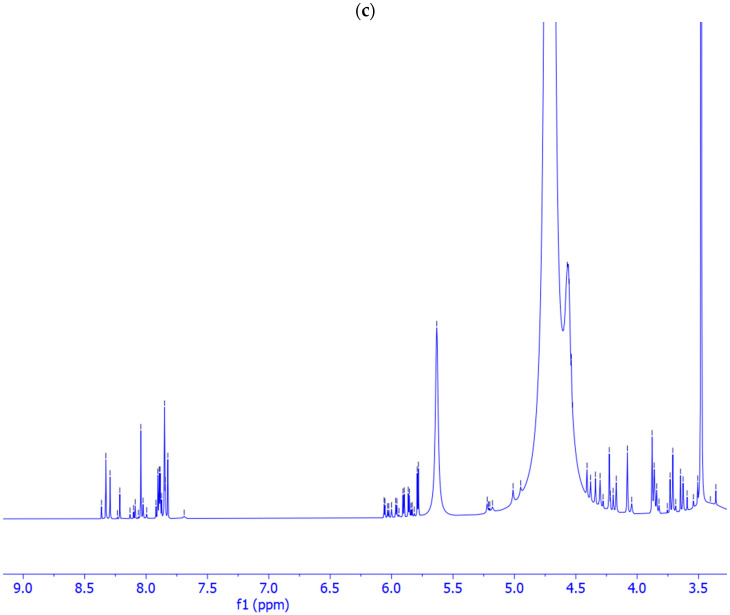
^1^H-NMR conversion (%) of adenosine to adenosine phosphates. (**a**) Zoomed part of ^1^H-NMR spectrum after deconvolution process; (**b**,**c**) Comparison of the original ^1^H- NMR spectrum with the corresponding deconvolution spectrum of phosphorylation reaction of adenosine using urea as additive in water, under evaporative conditions at 65–70 °C. In Figure (**b**) the black spectrum is before deconvolution while (**c**) blue spectrum is after the deconvolution process as discussed previously [46].

**Figure 9:**
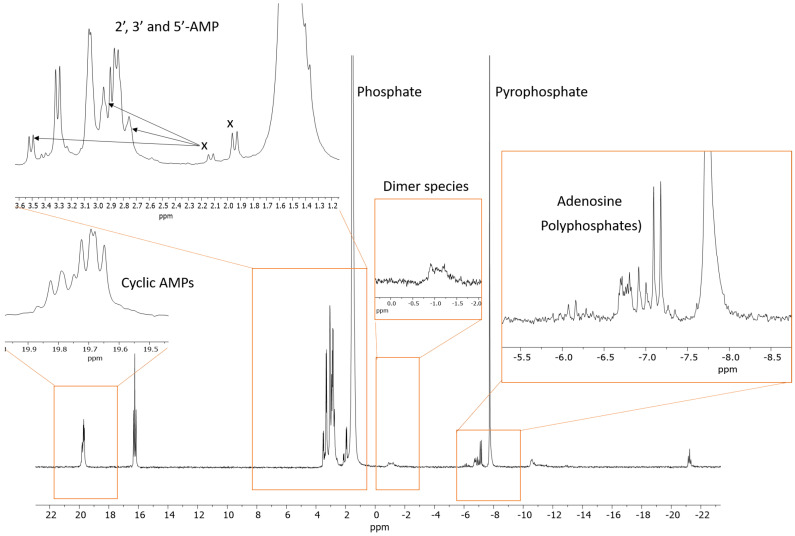
^31^P-NMR, H-coupled spectrum of the reaction sample containing a solution of adenosine, ammonium phosphate and urea (Sample AD-Urea), heated at 65–70 ^°^C, under unsealed (evaporative) conditions for 2 days. Various P products from right to left are as follows: linear triphosphate (and/or ATP), pyrophosphate, ADP and other adenosine polyphosphates, dimer of adenosine-phosphate-adenosine, 2′,3′ and 5′ monophosphates, and adenosine diphosphates (individual phosphate attached to at least 2 carbons of the ribose sugar and labeled as species ‘x’), PAA (internal standard), and cyclic AMPs.

**Figure 10:**
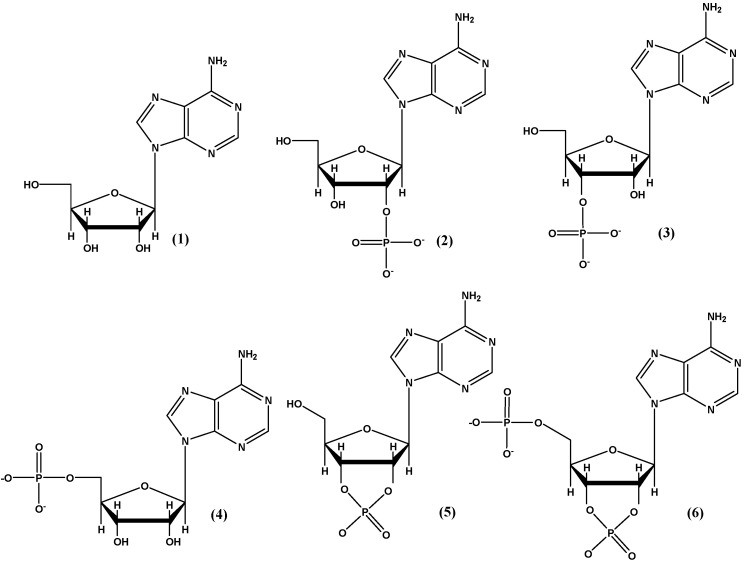
Structures of selected (and major) adenosine phosphates that were identified and detected based on both ^1^H-NMR and ^31^P-NMR (H-coupled and decoupled). Note that structures X shown in ^1^H-NMR are not included here.

**Figure 11:**
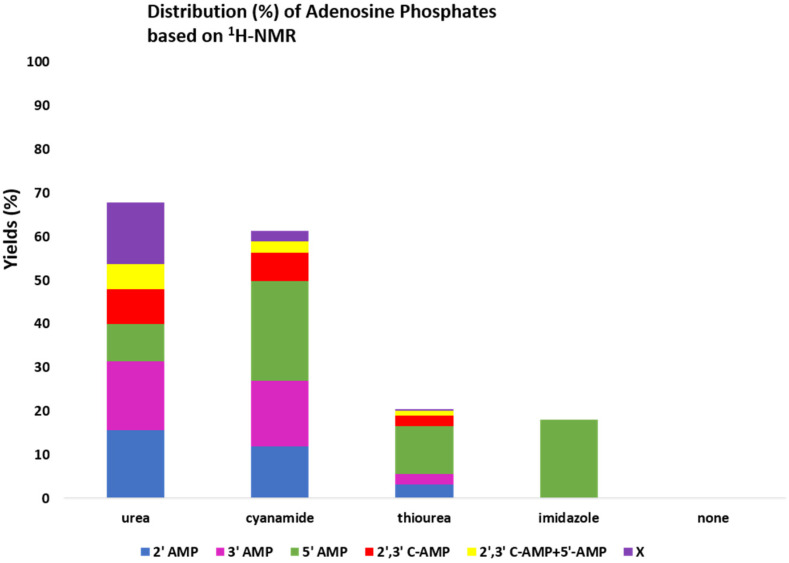
Comparison of various adenosine P species obtained in the presence of various additives. Conversions (%) of adenosine to adenosine phosphates based on Table 4.

To explore prebiotically relevant pathways, we tested a set of four nitrogen-containing additives that have shown significant potential in facilitating phosphorylation reactions under prebiotic conditions [36]. While these additives have demonstrated activity in past studies, they had not yet been systematically compared under conditions where orthophosphate serves as the phosphorylating agent at moderately elevated temperatures (65–70 °C), as opposed to the higher temperature regimes explored in our earlier work [46]. These comparative studies were conducted to assess the efficiency and plausibility of prebiotic phosphorylation pathways at more geochemically accessible temperature windows (Figure 8, Figure 9, Figure 10 and Figure 11, Table 4).

The phosphorus-containing products were confirmed using ^31^P-NMR (both proton-coupled and decoupled modes), ^1^H-NMR (Figure 8), and mass spectrometry (MS) targeting specific molecular weights. Major adenosine phosphate species were further verified by spiking samples with authentic standards. Among these techniques, ^31^P-NMR in the proton-coupled mode proved particularly effective for distinguishing various phosphorus species in the samples (Table 4, Figure 9, Figure 10 and Figure 11).

The identified ^31^P-NMR signals included inorganic phosphate as a singlet near 2–3 ppm, depending on the solution pH; pyrophosphate as a signal between −6 and −8 ppm; linear triphosphate presenting as a triplet and a doublet around −21 ppm and −6 to −8 ppm, respectively; dimeric adenosine-phosphate-adenosine species around –1 ppm; 2′- and 3′-AMP as doublets near 3–4 ppm; and 5′-AMP as a triplet at approximately 3–3.5 ppm, with some peak overlap among these species.

Additional smaller doublets and triplets, labeled as ‘x’ in Table 4 and Figure 9, were observed between 2.5 and 4 ppm. These signals likely correspond to diphosphate species in which two phosphate groups are attached separately to 2′ and 5′, 2′ and 3′, or 3′ and 5′ positions, rather than as adenosine pyrophosphate (or even all three). A multiplet at 19–20 ppm corresponded to 2′,3′-cAMP. Notably, two sets of peaks were present around 19–20 ppm: one assigned to 2′,3′-cAMP and another likely representing 2′,3′-cyclic, 5′-monophosphate), consistent with our previous findings [34]. We did not detect any 3′,5′-cAMP, which typically appears near 0 to −2 ppm [34].

As mentioned previously, phosphorylation reactions were conducted using four nitrogen-containing additives—urea, cyanamide, imidazole, and thiourea—alongside a control without any additives. Among these, urea yielded the highest conversion (~68%), followed by cyanamide. Thiourea and imidazole were less effective, achieving only 18–20% conversion (Figure 11). Under our evaporative heating conditions (65–70 °C), no adenosine phosphate products were detected in the absence of these condensing agents, highlighting their critical role in facilitating phosphorylation. Small amounts (~2%) of ADP were detected in samples containing urea and cyanamide, but no ATP formation was observed in any of the synthesis reactions.

### 2.3. Numerical Modeling of Degradation and Rate of Production

Our experimental data determined that ATP and ADP demonstrated the highest susceptibility to hydrolysis, initiating degradation at temperatures as low as 22–25 °C in doubly deionized water (DDI). Furthermore, although ATP retained about 98% of its integrity over four days at room temperature, its stability rapidly declined with increasing temperature. At 50–55 °C, ATP was nearly fully degraded within 48 h, corresponding to a half-life (Table 5) of approximately 0.5 days. Above 55 °C, degradation accelerated further, suggesting a threshold beyond which ATP is unstable even after a few hours.

Under saltwater conditions, ATP exhibited an extended half-life at room temperature (Table 5). In contrast, ADP was the least stable nucleotide across all tested conditions, irrespective of solvent (saltwater or ultrapure water). AMP displayed greater thermal stability in saline solutions compared to DDI water; however, at 70–75 °C, AMP was more stable in DDI water than under equivalent saline conditions (Table 5).

**Figure 12:**
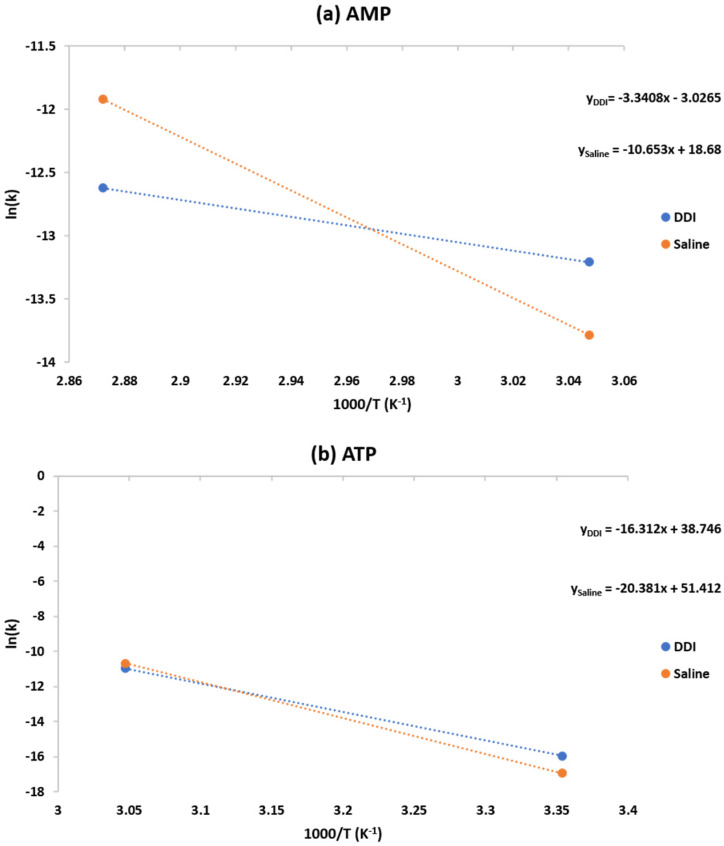
Arrhenius plots of AMP (**a**) and ATP (**b**).

Additionally, an Arrhenius analysis of degradation kinetics showed difference in activation energy profiles for AMP and ATP with respect to medium. We note here that since the total number of points for the compounds that show incomplete hydrolysis is limited to, at best, two temperatures, making wholesale claims as to their activation energies should be performed with this limitation in mind. That said, ATP exhibited significantly higher activation energies—135.6 kJ/mol in DDI and 169.4 kJ/mol in saltwater—compared to AMP, which required only 27.8 kJ/mol in DDI and 88.6 kJ/mol in saltwater. The elevated activation energies in saline conditions could imply ionic shielding effects that stabilize the phosphate but raise the energy threshold for reaction initiation. See Table 6 for activation energies for AMP and ATP in both DDI and saltwater, along with Figure 12 for Arrhenius plots for the compounds. A more detailed temperature–time profile of these compounds could provide better constraints on their hydrolytic stability as a function of temperature.

Steady state calculations incorporating adenosine, phosphate, and various prebiotically relevant additives were performed for a solution of 0.935 mmol of adenosine, 2.65 mmol of phosphate (as ammonium dibasic salt), and of additives (4.75 mmol of cyanamide, 2.93 mmol of imidazole, 2.62 mmol of thiourea, and 3.33 mmol of urea) in 5 mL of DDI. Results show that formation of AMP, regardless of additive, increases in concentration and then reaches steady state in less than a day. Results are consistent with NMR analysis from Section 2.2 such that among the tested compounds, urea significantly outperformed others as an additive, yielding AMP at a rate of 0.073 g/day.

**Figure 13:**
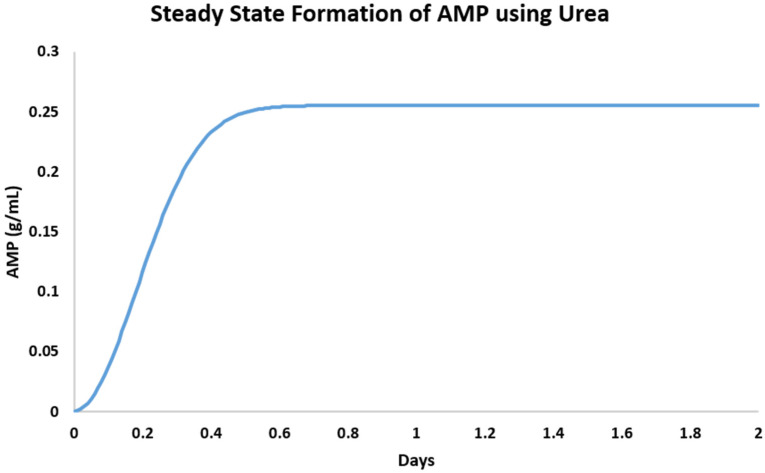
Steady state formation of AMP using urea as an additive. Calculations were performed for solutions of 5 mL.

**Figure 14:**
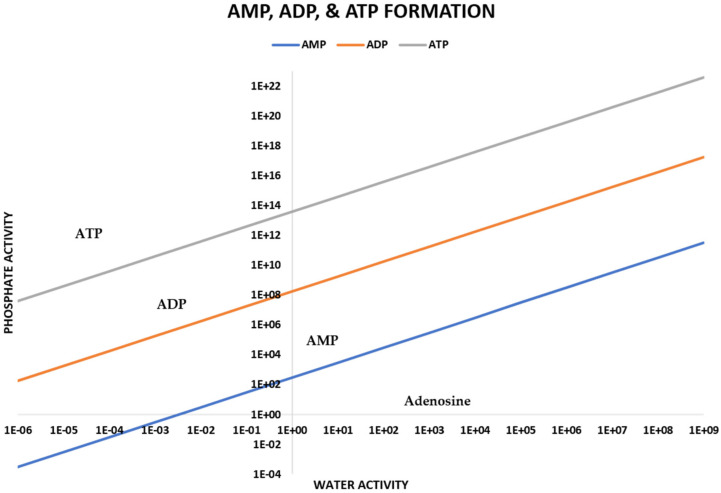
AMP, ADP, and ATP from Adenosine and Phosphate at 298 K.

Cyanamide followed at 0.065 g/day, while thiourea and imidazole produced lower yields (0.022 and 0.019 g/day, respectively). See Figure 13 for steady-state plots of AMP formation and to Table 7 for the corresponding rates of synthesis as a function of additive. Furthermore, we calculate here the ability to generate AMP, ADP, and ATP from adenosine and phosphate at 298K as a function of phosphate and water activity (Figure 14) using thermodynamic data from Pasek (2019) [15]. As can be expected, the generation of ATP requires much lower water activities or higher phosphate activities for its production at equilibrium than ADP and AMP.

## 3. Discussion

We report the decomposition rates of AMP, ADP, and ATP at temperature windows of 22–25 °C, 50–55 °C, 70–75 °C, and 85–90 °C, over heating periods of 2 and 4 days, in both simulated saltwater conditions and ultrapure water. Overall, ADP and ATP are readily hydrolyzed at temperatures above 50 °C. The major decomposition products identified include inorganic phosphate, pyrophosphate, triphosphate, and 2′,3′-cAMP. The formation of 2′,3′-cAMP in some AMP decomposition samples was somewhat unusual and suggests an equilibrium between AMP and cAMP under anhydrous conditions.

Longer heating durations generally promoted condensation reactions and the formation of condensed phosphorus compounds, particularly in AMP samples. These observations complement previous findings where 3′,5′-cyclic nucleotides were synthesized in formamide at elevated temperatures through intramolecular cyclization of 5′-phosphorylated nucleosides, especially in the presence of additives such as carbodiimides [47].

The thermal and chemical stability of adenosine phosphates—AMP, ADP, and ATP—varies significantly with temperature and solution conditions with experimental data show that these compounds degrade rapidly under heat and dehydration, particularly above 22–25 °C. ATP, for example, is highly unstable at elevated temperatures. In deionized water, its half-life drops from 54 days at 22–25 °C to just 0.5 days at 50–55 °C. In saltwater, ATP is more stable at lower temperatures, with a half-life of 183 days at 22–25 °C, but still degrades rapidly at higher temperatures. Therefore, close to room temperature, it could be regarded as being stable, but the evidence of slight degradation may point to other factors that may play a role in degradation such as acidity (pH) or reaction time durations would merit future studies.

On the other hand, AMP is comparatively more stable. At 50–55 °C, its half-life is 2.43 days in DDI and 1.2 days in saltwater, while at lower temperatures, it was unaffected by hydrolysis. ADP, however, stands out for its extreme sensitivity to temperature. It was found to be stable only at 22–25 °C and completely hydrolyzed at higher temperatures under both DDI and saltwater conditions. This is particularly significant because ADP is a central intermediate in the phosphorylation cycle, linking the conversion of AMP to ATP and vice versa. Its rapid degradation at elevated temperatures would disrupt this cycle, making it difficult to sustain energy transfer processes essential for early biochemical systems during the Early Earth. This instability presents a paradox: while the synthesis of adenosine phosphates is thermodynamically favored at higher temperatures, their decomposition rates increase sharply, especially for ADP and ATP. To this end, results suggest that for these molecules to accumulate and persist in prebiotic environments, protective mechanisms or microenvironments—such as mineral surfaces, lipid compartments, or cyclic hydration-dehydration systems—must have existed to stabilize them or facilitate their continuous regeneration.

These findings highlight the balance between synthesis and degradation that existed under early Earth conditions. The short half-lives of these compounds at prebiotically relevant temperatures imply that a continuous source or recycling mechanism would have been essential to maintain sufficient concentrations of these molecules to support prebiotic metabolic networks. This would also require an environment with dynamic temperature gradients, wet–dry cycles, or catalytic surfaces that could potentially act as a buffer against rapid molecular decay. In addition, because results highlight temperature as a key factor in rate of degradation it is important to understand the influence Earth’s past climate had on these compounds. During the Hadean Eon, Earth’s surface experienced intense heat due to accretion, radioactive decay, and frequent impacts. However, as suggested by Kadoya and colleagues [48], average surface temperatures could have declined to as low as −14 °C during the late Hadean, partially due to atmospheric CO_2_ reduction via weathering of impact ejecta. Furthermore, Trail and colleagues have supported this hypothesis through studies of ~4.3 Ga zircon crystals from Jack Hills [49], which indicate crustal formation temperatures near 700 °C; this evidence implies Earth’s surface cooled sufficiently to permit the development of solid crust despite episodic molten conditions. Consequently, temperature fluctuations during this period were significant for prebiotic chemistry, with phases too extreme to allow compound stability—any molecules formed under cooler conditions may have been rapidly degraded. Conversely, if colder intervals reached −14 °C, such temperatures could have hindered prebiotic formation altogether.

Analyses of oxygen isotope ratios in cherts in the Archean suggest ocean temperatures ranged between 55 °C and 85 °C, although it remains debated whether these values represent global averages or more localized environments [50]. Surface temperatures may have varied from 0 °C to 40 °C, moderated by atmospheric CO_2_ levels and volcanic activity, supporting a stable hydrosphere potentially favorable to early microbial life [51]. This scenario may have represented a region where the rates of degradation of AMP, ADP, and ATP were sufficient to facilitate the emergence of other molecular species, bolstering prebiotic processes in warm pond environments.

Based on the ^31^P-NMR studies, we observed that under our evaporative pool models, in general, higher temperatures led to faster decomposition rate. For example, most organophosphorus species were completely degraded at the highest temperature window of 85–90 °C. Among the compounds studied, AMP was the most stable species. Thermodynamics provides a valuable framework for interpreting the stability of organic phosphorus compounds (and compounds in general) [15]. Specifically, the free energies of hydrolysis (ΔG) of various adenosine phosphates can be used to assess their relative stabilities and to better understand the decomposition trends observed in our experiments. The standard hydrolysis reactions for these three compounds at pH 7 are as follows [15,52]:

(i) ATP → ADP + Pi: ΔG′ = −30.5 kJ/mol

(ii) ADP → AMP + Pi: ΔG′ = −32.8 kJ/mol

(iii) AMP → Adenosine + Pi: ΔG′ = −14.2 kJ/mol

Note that the last reaction (iii) is rarely considered physiologically relevant but can still be used for comparison. When comparing the stabilities of ATP, ADP, and AMP using their standard free energies of hydrolysis (ΔG′), it is important to remember that a more negative ΔG′ indicates a molecule is less stable and more prone to hydrolysis, whereas a less negative ΔG′ suggests greater stability and resistance to hydrolysis. Based on this, AMP is the most thermodynamically stable of the three, ADP is the least stable (with its hydrolysis being the most energetically favorable), and ATP is much less stable than AMP but still slightly more stable than ADP. This observation aligns well with our experimental results shown in Table 2 and Table 3, particularly for reactions in ultrapure water.

Although it might seem counterintuitive, given that ATP is known as a “high-energy” molecule, this term refers to the amount of energy released upon hydrolysis, not the inherent stability of the molecule itself. Interestingly, ATP’s slightly greater thermal stability compared to ADP may help explain why ATP serves as the primary energy currency in biological systems—it resists decomposition better, enabling more efficient energy storage and transfer. To this end, kinetically, these compounds are formed at a relatively rapid rate, as observed when studying the formation of AMP with additives, as well as the production of ATP, ADP, and AMP in relation to water activity. Additionally, environmental factors play a critical role in their kinetics, with activation energies increasing in saline conditions. These considerations highlight the necessity of maintaining rigorous temperature controls during the generation of these compounds, since elevated temperatures accelerate their degradation.

Modern terrestrial environments that resemble the prebiotic pool scenarios suggested in our study include Yellowstone National Park (USA) [53], the Dallol hydrothermal system (Ethiopia) [54], Tatio Geysers (Chile) [55], the Kamchatka Peninsula (Russia) [56], and Rotorua (New Zealand) [57]. These sites are characterized by shallow pools or streams with temperatures ranging from mildly hot (~30–50 °C) to boiling. The pH typically ranges between 2 and 6, with some areas exhibiting values around 4 to 5. Additionally, these environments are often rich in dissolved minerals, sulfur, and various metals, making them plausible analogues for early Earth prebiotic niches.

We utilized nitrogen-containing additives in the synthesis of adenosine phosphates. Using urea, we observed high yields of various adenosine phosphates (~68%), consistent with previous reports [32,33,34,46]. Urea is a well-known additive that facilitates condensation reactions under prebiotic conditions [19,33,34,36,46,58,59]. Similarly, cyanamide and imidazole also enhance phosphorylation rates, especially when high-energy P–N species such as diamidophosphates (DAPs) are employed [36]. The role of thiourea in potentially facilitating prebiotic phosphorylation requires further investigation, although it is recognized as a prebiotically relevant compound [59,60,61]. Furthermore, these N-containing additives seem to play two significant roles; 1) these seem to enhance the dehydration reactions, and 2) these can also potentially represent small organic molecules present in ‘prebiotic messy environments’ [20,36,59,60,61,62]. Overall, yields ranging from 18% to 68% were obtained, supporting the notion that warm evaporative environments promote these reactions.

However, the relatively short half-lives and instability of adenosine phosphates—particularly ADP and ATP—suggest that warm to mildly hot evaporating pools may not favor the long-term accumulation of these organophosphorus compounds due to harsh thermal conditions. The steady-state concentrations of these compounds may have been limited. It is possible that Darwin’s proposed prebiotic pools [43] enabled formation but not the prolonged accumulation or polymerization of such molecules. Once synthesized, these compounds might have been transferred to interconnected pools with more moderate conditions (e.g., temperatures below 50 °C and near-neutral pH), where they could accumulate and participate in further reactions.

The idea that all abiotic reactions took place within a single pond or environment on the early Earth may be overly simplistic. Instead, the concept of multiple, interconnected pools or ponds, linked via streams or rainfall to enable mixing and multi-step prebiotic chemistry, has been previously suggested by Clarke and Kolb [63], Sutherland [64], Damer and Deamer [17,41], as well as supported by our own phosphorylation studies [34].

## 4. Materials and Methods

The chemicals required for the experiments included the following: 5′- AMP (adenosine monophosphate), 5′-ADP (adenosine diphosphate), 5′-ATP (adenosine triphosphate), Adenosine-2′,3′-cyclic monophosphate or 2′,3′-cAMP (all as sodium salts) from Sigma Aldrich (Burlington, MA, USA), EDTA (Ethylenediaminetetraacetic Acid), sodium hydroxide and Ammonium Phosphate Dibasic from Fisher Scientific (Waltham, MA, USA), and deuterium oxide, urea, thiourea, imidazole, cyanamide from Acros Organic (Burlington, MA, USA).

Instant Ocean was obtained from Pentair Aquatic Eco Systems Inc. (Apopka, FL, USA). The product, Instant Ocean Sea Salt, consists primarily of NaCl (about 58%). Typically, Instant Ocean contains NaCl, MgCl_2_, MgSO_4_, CaCl_2_, and KCl/K_2_SO_4_. It is formulated in a way that dissolving 35 g of the mixture in pure (or doubly deionized) water yields a solution with the following ionic concentrations: 0.55 M Cl^−^, 0.47 M Na^+^, 0.05 M Mg^2+^, 0.028 M SO_4_^2+^, 0.01 M Ca^2+^, and 0.01 M K^+^. The composition is designed to approximate that of modern seawater. Importantly, the mixture does not contain any phosphate salts, as confirmed by our ^31^P-NMR analysis, which showed no detectable phosphate.

Doubly deionized water (DDI, hereafter) was obtained in-house by using a Barnstead NANO pure^®^ Diamond Analytical combined reverse osmosis–deionization system. Na_4_EDTA solution was prepared as reported previously [44].

The pH of various solutions was measured using Fisherbrand™ Hydrion pH paper (Fisher Scientific, Waltham, MA, USA). The initial pH of the aqueous solutions containing the standard compounds AMP, ADP, and ATP was 4, possibly due to the presence of free acids alongside their sodium salts. In all decomposition study sets, the starting pH was 4. After the reactions were completed, the pH remained constant for all reaction samples studied at 22–25 °C. However, for reaction samples heated at 50 °C and above, the pH of the solutions had decreased. Upon rehydration of the dried reaction samples (for pH testing), the final pH was 3.

(a) Decomposition reactions of AMP, ADP and ATP

^31^P-NMR of AMP, ADP and ATP prior to reactions did not show any P impurities or decomposition P products (Supplementary Information, Figures S1–S3).

**Set AMP-DDI:** Decomposition reactions of 5′-AMP were investigated under controlled conditions. In Set No (AMP-DDI)-2days, 0.055 mmol (solid) of 5′-AMP was added to a clean 20 mL glass vial, followed by the addition of 4 mL of ultrapure or doubly deionized (DDI) water. The reaction mixtures were stirred continuously at four different temperature ranges: 22–25 °C, 50–55 °C, 70–75 °C, and 85–90 °C for a duration of 2 days. In Set No (AMP-DDI)-4days, all experimental conditions were identical to those in Set No (AMP-DDI)-2days, except that the reactions were allowed to proceed for 4 days instead of 2 days.

**Set AMP-SW**: Decomposition reactions of 5′-AMP were studied in the presence of an Instant Ocean saltwater mixture. In Set No (AMP-SW)-2days, for each reaction, 0.055 mmol of 5′-AMP and 0.1 g of Instant Ocean salt mixture were added to a clean 20 mL glass vial, followed by the addition of 4 mL of ultrapure or doubly deionized (DDI) water. The mixtures were stirred continuously at one of the following temperature ranges: 22–25 °C, 50–55 °C, 70–75 °C, or 85–90 °C for a duration of 2 days. In Set No (AMP-SW)-4days, all conditions were identical to those in Set No (AMP-SW)-2days, except that the reactions were allowed to proceed for 4 days instead of 2 days.

**Set ADP-DDI**: Decomposition reactions of ADP were studied under controlled conditions. In Set No (ADP-DDI)-2days, for each reaction, 0.045 mmol of ADP was added to a clean 20 mL glass vial, followed by the addition of 4 mL of ultrapure or doubly deionized (DDI) water. The reaction mixtures were stirred continuously at one of the following temperature ranges: 22–25 °C, 50–55 °C, 70–75 °C, or 85–90 °C for a duration of 2 days. In Set No (ADP-DDI)-4days, all conditions were identical to those in Set No (ADP-DDI)-2days, except that the reactions were allowed to proceed for 4 days instead of 2 days.

**Set ADP-SW**: Decomposition reactions of ADP were studied in the presence of Instant Ocean salt mixture. In Set No (ADP-SW)-2days, for each reaction, 0.045 mmol of 5′-ADP and 0.1 g of Instant Ocean salt mixture were added to a clean 20 mL glass vial, followed by the addition of 4 mL of ultrapure or doubly deionized (DDI) water. The mixtures were stirred continuously at one of the following temperature ranges: 22–25 °C, 50–55 °C, 70–75 °C, or 85–90 °C for 2 days. In Set No (ADP-SW)-4days, all conditions were identical to those in Set No (ADP-SW)-2days, except that the reactions were allowed to proceed for 4 days instead of 2 days.

**Set ATP-DDI**: Decomposition reactions of ATP were studied under controlled conditions. In Set No (ATP-DDI)-2days, for each reaction, 0.038 mmol of ATP was added to a clean 20 mL glass vial, followed by the addition of 4 mL of ultrapure or doubly deionized (DDI) water. The reaction mixtures were stirred continuously at one of the following temperature ranges: 22–25 °C, 50–55 °C, 70–75 °C, or 85–90 °C for 2 days. In Set No (ATP-DDI)-4days, all conditions were identical to those in Set No (ATP-DDI)-2days, except that the reactions were allowed to proceed for 4 days instead of 2 days.

**Set ATP-SW**: Decomposition reactions of ATP were studied in the presence of Instant Ocean salt mixture. In Set No (ATP-SW)-2days, for each reaction, 0.038 mmol of 5′-ATP and 0.1 g of Instant Ocean salt mixture were added to a clean 20 mL glass vial, followed by the addition of 4 mL of ultrapure or doubly deionized (DDI) water. The mixtures were stirred continuously at one of the following temperature ranges: 22–25 °C, 50–55 °C, 70–75 °C, or 85–90 °C for 2 days. In Set No (ATP-SW)-4days, all conditions were identical to those in Set No (ATP-SW)-2days, except that the reactions were allowed to proceed for 4 days instead of 2 days.

For all reaction sets, to mimic drying or evaporating pool conditions, the reaction vials were kept unsealed during the experiments. The reaction vials (in each sub-set) studied at temperature window of 22–25 °C remained as a solution due to very low evaporation rates of water under ambient conditions.

(b) Condensation agents mediated prebiotic syntheses of adenosine phosphates

In a typical phosphorylation study, adenosine (0.935 mmol), phosphate as ammonium dibasic salt (NH_4_)_2_HPO_4_) (2.65 mmol) and (4.16–5.95 mmol) of an additive (if any) were added to 5 mL DDI water in a 20 mL capacity clean glass vials. One reaction sample was run without any additive. The starting pH of each solution was 8.5. The reaction samples were stirred with the help of a magnetic stirrer and were left unsealed. The reaction samples were heated at 65–70 °C to mimic a dry-evaporating pool. To compare the effectiveness of the additives, each one was added to a separate reaction vial containing phosphate and adenosine. Various additives included urea, thiourea, cyanamide and imidazole. The reaction vials were allowed to heat, unsealed (under evaporative conditions) for 2 days.

(c) NMR studies of organophosphates

Reaction samples that were heated unsealed at elevated temperatures (>22–25 °C) were allowed to cool to room temperature upon completion of the reaction. Each sample was then prepared for analysis as follows: To each cooled reaction mixture, 1 mL of aqueous Na_4_EDTA solution was added and stirred at room temperature (for 20 min) using a magnetic stirrer. From this mixture, approximately 0.25 mL was transferred to an Eppendorf tube, and D_2_O was added to bring the total volume to 1 mL. The sample was then centrifuged for 10–15 min, or until insoluble particulates settled at the bottom.

For NMR analysis, 450 µL of the supernatant was transferred to a clean NMR tube, followed by the addition of 15 µL of Na_4_EDTA and 20 µL of 0.1 M phosphonoacetic acid to serve as an NMR standard (PAA) as well as a reference peak. Na_4_EDTA was added twice to ensure the removal of any residual metal impurities from the starting materials and to improve the quality of peak splitting in the ^31^P-NMR spectrum. The NMR tube was capped, shaken thoroughly to ensure homogeneity, and then analyzed.

For each reaction sample studied at the temperature range of 22–25 °C, 0.25 mL of the solution was transferred to an Eppendorf tube, and 0.75 mL of D_2_O was added to bring the total volume to 1 mL. The sample was then centrifuged for 10–15 min to allow any insoluble material to settle.

As described above, 450 µL of the supernatant was transferred to a clean NMR tube, followed by the addition of 15 µL of Na_4_EDTA and 20 µL of 0.1 M phosphonoacetic acid (PAA). The tube was capped, mixed thoroughly, and prepared for NMR analysis.

All NMR measurements were performed using the Champlain–Bruker WB 600 MHz spectrometer. To quantify 5′-AMP, ADP, ATP, and various other phosphorus-containing products in the reaction mixtures, ^31^P-NMR spectroscopy was employed. The quantification was carried out by replicating the ^31^P-NMR acquisition parameters as described previously [45,46], with the following settings: number of scans = 64, data points = 256 K, relaxation delay (D1) = 20 s, and sample temperature = 298 K [45].

Samples were analyzed in both proton-coupled and proton-decoupled ^31^P-NMR modes. The primary modification from the previously reported method [45] was the use of phosphonoacetic acid (PAA) as an internal standard instead of sodium 3-(trimethylsilyl)propionate-2,2,3,3-d_4_ (TMSP) or anhydrous disodium hydrogen phosphate (Na_2_HPO_4_). This substitution was made in part because orthophosphate, a common byproduct of decomposition, can overlap with internal standards. In contrast, PAA appears as a well-resolved triplet in the proton-coupled ^31^P-NMR spectrum at 14–15 ppm, with minimal risk of overlapping with signals from the targeted phosphorus products.

Phosphorus-containing products were identified by comparing chemical shifts and coupling constants to those of known standards and previously reported values [46].

Additionally, phosphorylation reaction studies in subset (b) were analyzed using both ^31^P-NMR and ^1^H-NMR. Conversion percentages were determined from ^1^H-NMR spectra following methods described previously [36,46].

(d) Mass Spectrometry of organophosphates

Mass spectra were obtained using a Q Exactive Orbitrap mass spectrometer (Thermo Fisher Scientific) coupled to an electrospray ionization source. Spectral resolution was set at 70,000 and the data were collected in the negative ionization mode. 10 microliters of the original solution were diluted in 1 mL of DI water (1/100 dilution) and injected into the mass spectrometer to obtain mass spectral data.

(e) Theoretical Modeling of AMP, ADP and ATP decomposition and formation rates.

Coupling both empirical analyses obtained from NMR analysis with numerical modeling, we calculate the rate of decomposition and thereby the half-life of AMP, ADP and ATP. This allows us to better constrain the environmental factors that stymie the buildup of organic compounds that could potentially be utilized in prebiotic chemical process. Assuming a first-order reaction, we solve Equations (1)–(4) [65]. The rate constant, k, is obtained by the following equation:

(1) $$ln(\frac{{[A}_{0}]}{\left[A_{nmr}\right]})=kt$$

where [*A_nmr_*] is the percentage obtained from NMR analysis, [*A*_0_] is the initial amount of AMP, ADP, or ATP (100%), *k* is the rate constant, and *t* is time in days. Using this data, we set up a numerical model that anticipated concentration levels:

(2) $${\left[A\right]=[A}_{0}]*e^{-kt}$$

Furthermore, the half-life for the compounds were calculated utilizing the standard half-life equation below:

(3) $$t_{1/2}=\ln\left(2\right)/k$$

This model assumes a constant rate of decay derived from final concentration levels after four days to estimate concentrations at time points (one and three days).

In addition to modeling decomposition rates of AMP, ADP, and ATP, steady state production of compounds was also investigated in this study using the following equations:

(4) $$F_{x}=k[x]$$

where *F_x_* is the generation rate of compound (*x*).

Lastly, calculations to generate AMP, ADP, and ATP from adenosine and phosphate at 298 K as a function of phosphate and water activity was performed using thermodynamic data from [15].

## Figures and Tables

**Table 1:** Yields^[1]^ (%) of various P species detected in the reaction samples of AMP at a pH of 4.

Sample	Reaction Set No	Time	Temp.	AMP	PO_4_^3−^	P_2_O_7_^4−^	* Other P
		(Days)	°C				
AD-1	AMP-DDI	2	22–25	100	** BDL	BDL	BDL
AD-2	AMP-DDI	2	50–55	100	BDL	BDL	BDL
AD-3	AMP-DDI	2	70–75	86	10	4	BDL
AD-4	AMP-DDI	2	85–90	BDL	61	39	BDL
AD-5	AMP-DDI	4	22–25	100	BDL	BDL	BDL
AD-6	AMP-DDI	4	50–55	53	37	10	BDL
AD-7	AMP-DDI	4	70–75	32	20	41	7
AD-8	AMP-DDI	4	85–90	BDL	51	49	BDL
AD-9	AMP-SW	2	22–25	100	BDL	BDL	BDL
AD-10	AMP-SW	2	50–55	75	18	7	BDL
AD-11	AMP-SW	2	70–75	42	26.5	7	24.5
AD-12	AMP-SW	2	85–90	BDL	100	BDL	BDL
AD-13	AMP-SW	4	22–25	100	BDL	BDL	BDL
AD-14	AMP-SW	4	50–55	70	30	BDL	BDL
AD-15	AMP-SW	4	70–75	11	51	38	BDL
AD-16	AMP-SW	4	85–90	BDL	69.5	30.5	BDL

Yields^[1]^ (%) of various P species present in the reaction samples. The yields (%) were calculated using the peak integration method of ^31^P-NMR [19,46]. * Other P species: any other P compounds that were not frequently observed in the samples and in this case, we mainly observed 2′,3′-cAMP (besides sample AD-11, in which we observed AMP-diphosphate species, not as pyrophosphates but individual phosphate group attached to two of the 2′, 3′ and/or 5′ positions). ** BDL means ‘below detection limit’, DDI means doubly deionized water, and SW stands for salt water.

**Table 2:** Yields^[1]^ (%) of various P species detected in the reaction samples of ADP at a pH of 4.

Sample	Reaction Set No	Time	Temp.	ADP	AMP	PO_4_^3−^	P_2_O_7_^4−^	* Other P
		(Days)	°C					
AD-17	ADP-DDI	2	22–25	100	** BDL	BDL	BDL	BDL
AD-18	ADP-DDI	2	50–55	BDL	11.5	65	23.5	BDL
AD-19	ADP-DDI	2	70–75	BDL	5	37	48	10
AD-20	ADP-DDI	2	85–90	BDL	BDL	27	49	24
AD-21	ADP-DDI	4	22–25	100	BDL	BDL	BDL	BDL
AD-22	ADP-DDI	4	50–55	BDL	19	49	32	BDL
AD-23	ADP-DDI	4	70–75	BDL	6	66	28	BDL
AD-24	ADP-DDI	4	85–90	BDL	4	35	46.5	14.5
AD-25	ADP-SW	2	22–25	100	BDL	BDL	BDL	BDL
AD-26	ADP-SW	2	50–55	BDL	5	54	41	BDL
AD-27	ADP-SW	2	70–75	BDL	BDL	29	71	BDL
AD-28	ADP-SW	2	85–90	BDL	BDL	42	58	BDL
AD-29	ADP-SW	4	22–25	100	BDL	0	BDL	BDL
AD-30	ADP-SW	4	50–55	BDL	BDL	40.5	59.5	BDL
AD-31	ADP-SW	4	70–75	BDL	BDL	29	61	10
AD-32	ADP-SW	4	85–90	BDL	BDL	30	63	7

Yields^[1]^ (%) of various P species present in the reaction samples. The yields (%) were calculated using the peak integration method of ^31^P-NMR [19,46]. * Other P species mean any other P compounds that were not frequently observed in the samples and in this case, we mainly observed inorganic triphosphate. ** BDL means ‘below detection limit’, DDI means doubly deionized water, and SW stands for salt water.

**Table 3:** Yields^[1]^ (%) of various P species detected in the reaction samples of ATP at a pH of 4.

Sample	Reaction Set No	Time	Temp.	ATP	ADP	AMP	PO_4_^3−^	P_2_O_7_^4−^	* Other P
		(Days)	°C						
AD-33	ATP-DDI	2	22–25	98	** BDL	BDL	2	BDL	BDL
AD-34	ATP-DDI	2	50–55	5	10	4	26	29	26
AD-35	ATP-DDI	2	70–75	BDL	10	BDL	23	43	24
AD-36	ATP-DDI	2	85–90	BDL	BDL	BDL	83	17	BDL
AD-37	ATP-DDI	4	22–25	96	2	BDL	2	BDL	BDL
AD-38	ATP-DDI	4	50–55	BDL	BDL	6	40.5	45.5	8
AD-39	ATP-DDI	4	70–75	BDL	BDL	BDL	76	24	BDL
AD-40	ATP-DDI	4	85–90	BDL	BDL	BDL	30	70	BDL
AD-41	ATP-SW	2	22–25	99	BDL	BDL	1	BDL	BDL
AD-42	ATP-SW	2	50–55	2	5	BDL	31	62	BDL
AD-43	ATP-SW	2	70–75	BDL	BDL	BDL	40	60	BDL
AD-44	ATP-SW	2	85–90	BDL	BDL	BDL	17	65	18
AD-45	ATP-SW	4	22–25	98.5	BDL	BDL	1.5	BDL	BDL
AD-46	ATP-SW	4	50–55	BDL	BDL	BDL	31.5	68.50	BDL
AD-47	ATP-SW	4	70–75	BDL	BDL	BDL	45	55	BDL
AD-48	ATP-SW	4	85–90	BDL	BDL	BDL	24	67	9

Yields^[1]^ (%) of various P species present in the reaction samples. The yields (%) were calculated using the peak integration method of ^31^P-NMR [19,46]. * Other P species mean any other P compounds that were not frequently observed in the samples and in this case, we mainly observed inorganic triphosphate. ** Where BDL means ‘below detection limits’, DDI means doubly deionized water, and SW stands for salt water.

**Table 4:** ^1^H-NMR conversions of adenosine to adenosine phosphates in the presence of various N-containing additives.

Additive	2′-AMP	3′-AMP	5′-AMP	2′,3′-AMP	2′,3′+5′-AMP	^c^ X	^b^ Total Yields
^1^H-NMR based ^a^ yields (%) of various adenosine P compounds							
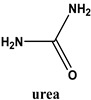 **(3.33 mmoles)**	15.70	15.70	8.65	8	5.7	14	68
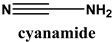 **(4.75 mmoles)**	12	15	22.77	6.5	2.7	2.31	61.2
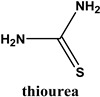 **2.62 mmoles**	3.17	2.38	11.11	2.38	1	0.5	20.54
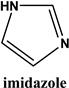 **2.93 mmoles**	BDL	BDL	18	BDL	BDL	BDL	18
**None**	BDL	BDL	BDL	BDL	BDL	BDL	BDL

Phosphorylation of adenosine using N-containing additives. ^a^ Percent (%) conversion yields were calculated by ^1^H-NMR (obtained after deconvolution of the spectrum). ^b^ Total yields were the sum of 2′-AMP, 3′-AMP, 5′-AMP, 2′,3′-cAMP, and other adenosine P species labeled as X. ^c^ Other adenosine P species (X) refer to various miscellaneous (adenosine diphosphate) species in which phosphate was attached to more than one carbon, e.g., one phosphate attached to 2′ and the other phosphate attached to 5′ positions, respectively. BDL means ‘below detection limits’.

**Table 5:** Calculated half-lives of compounds that were not fully stable or were not below detection level. DDI means doubly deionized water and SW stands for salt water.

Compound	Conditions	Half-Life (Days)
AMP	50–55 °C, DDI	4.37
AMP	70–75 °C, DDI	2.43
AMP	50–55 °C, SW	7.78
AMP	70–75 °C, SW	1.2
ATP	22–25 °C, DDI	54
ATP	50–55 °C, DDI	0.5
ATP	22–25 °C SW	183

**Table 6:** Activation energies utilizing data from Arrhenius plots for AMP and ATP.

Medium	AMP Activation Energy (J/mol)	ATP Activation Energy (J/mol)
DDI	27.8	135.6
Saline	88.6	169.4

**Table 7:** Rate of AMP synthesis with respect to additive. Note higher yields per day when using urea as additive.

Additive	Rate of Synthesis (g/days)	Steady State Maxima (g)	Concentration (g/mL)
Urea	0.073	0.26	0.051
Cyanamide	0.065	0.23	0.046
Thiourea	0.022	0.08	0.015
Imidazole	0.019	0.07	0.013

## Data Availability

Data are contained within the article or Supplementary Material. The original contributions presented in this study are included in the article Supplementary Material. Further inquiries can be directed to the corresponding authors.

## Supplementary Materials

The following supporting information can be downloaded at: https://www.mdpi.com/article/10.3390/molecules30173587/s1. Reference [66] is cited in the supplementary materials.
