# Supplementary material for: Thermal Decomposition and Prebiotic Formation of Adenosine Phosphates in Simulated Early-Earth Evaporative Settings
Source: Molecules. 2025 Sep 2;30(17):3587. doi: 10.3390/molecules30173587 (PMC12430388; doi:10.3390/molecules30173587)
Supplement: Supplementary file 1 [file molecules-30-03587-s001.zip › molecules-3832118-supplementary.pdf]

Supplementary Information

Thermal Decomposition and Prebiotic Formation of Adenosine Phosphates in Simulated Early-Earth Evaporative Settings

Maheen Gull[1,2,*] Christopher Mehta[3], Maria Jesus Herrero Perez[1,2], Annika Seeley[1,2], Karyn L. Rogers[1,2] and Matthew A. Pasek[1,2,*]

1. Department of Earth and Environmental Sciences, Rensselaer Polytechnic Institute, Troy, NY, USA
2. Rensselaer Astrobiology Research and Education Center (RARE), Rensselaer Polytechnic Institute, Troy, NY, USA
3. Howard University Plasma Lab (HUPL), Howard University, Washington, DC, USA

Correspondence email: pasekm@rpi.edu; gullm@rpi.edu

Figure S1. ^{1}H-Coupled ^{31}P-NMR spectrum of pure 5′-AMP.

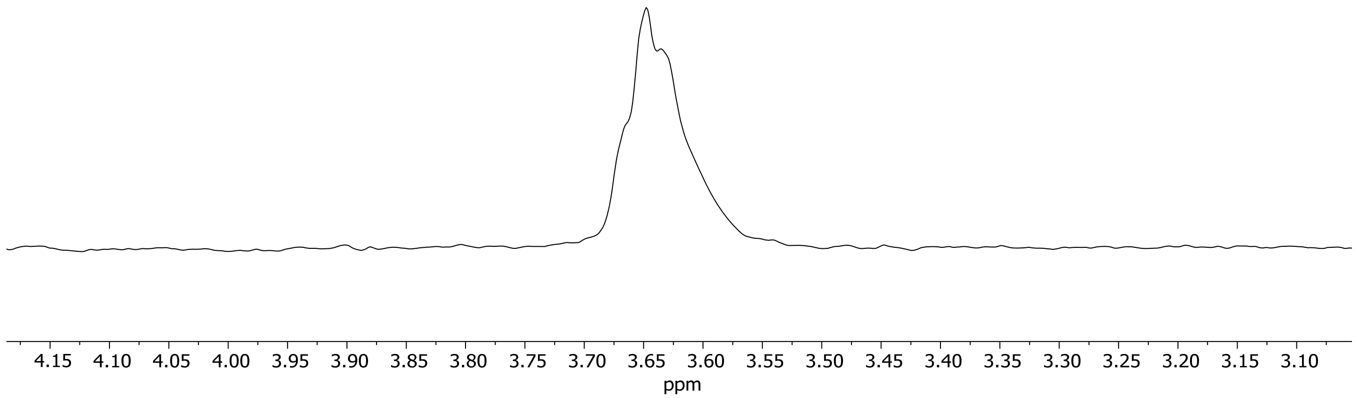

Fig. S1. In ^{1}H-coupled mode of ^{31}P-NMR, 5′-AMP appears to be a triplet around 3.50-3.60 ppm. The peaks were referenced by using PAA (phosphonoacetic acid). Also, each sample was extracted with Na_4EDTA to remove the metallic impurities from pure adenosine phosphates [1].

Figure S2. ^{31}P-NMR spectra of pure 5′-ADP.

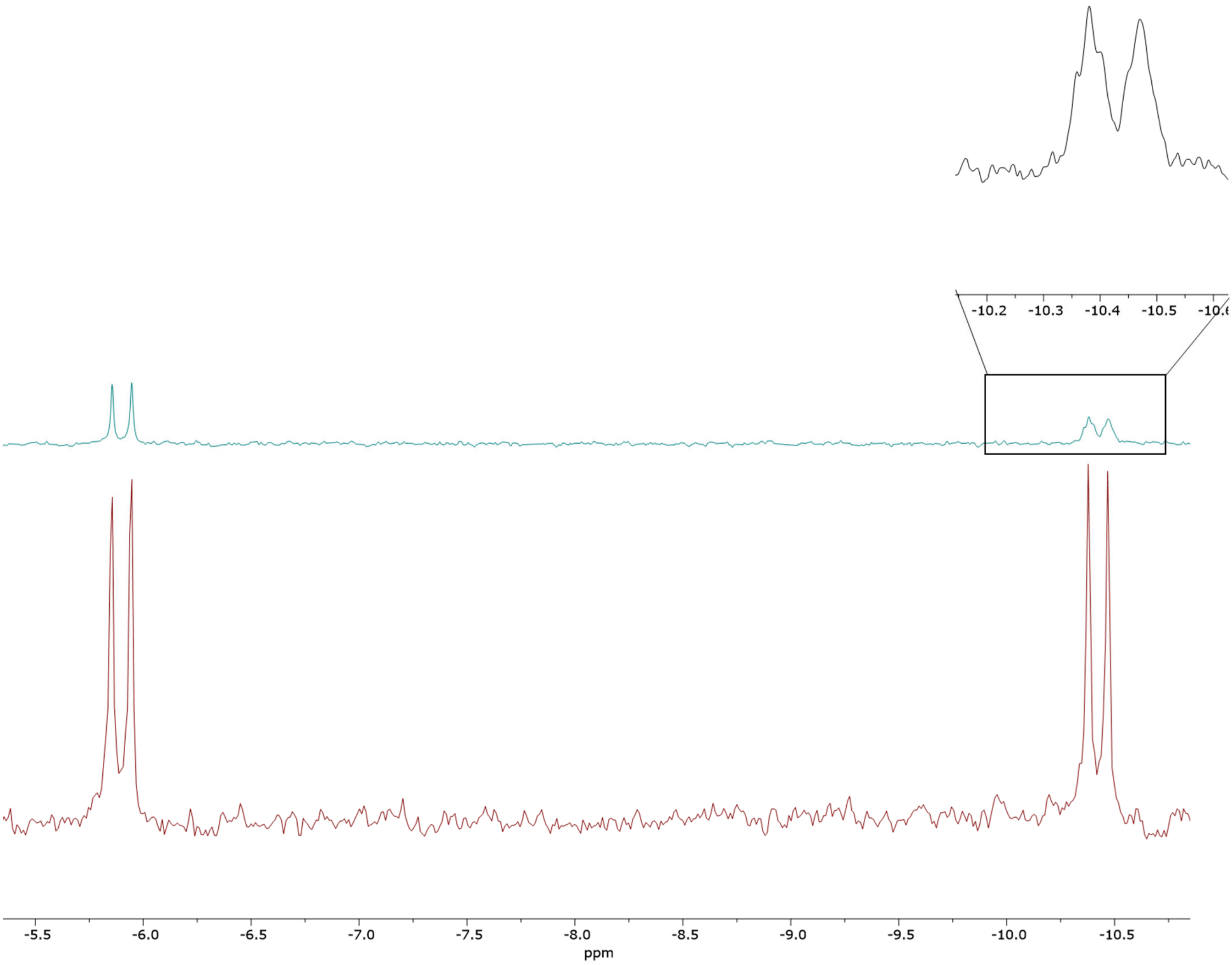

Figure S2. ^{31}P-NMR ^{1}H-decoupled spectrum (bottom) and ^{1}H-coupled spectrum (top). In ^{31}P-NMR (^{1}H-decoupled), there are two wide doublets while in case of H-coupled ^{31}P-NMR, the doublet around -10ppm splits into a doublet of triplets.

Figure S3. ^{31}P-NMR spectra of pure 5′-ATP.

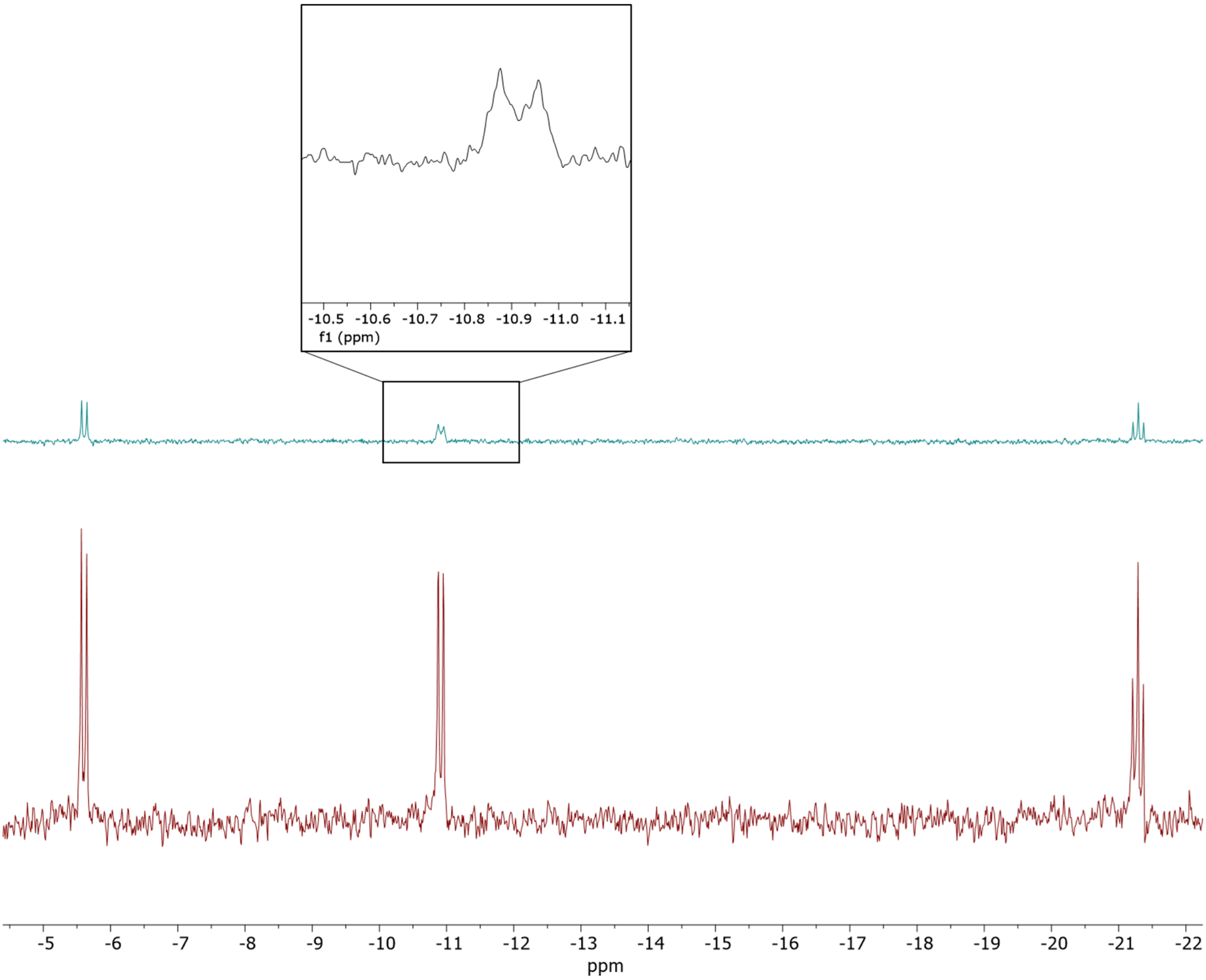

Figure S3. ^{31}P-NMR ^{1}H-decoupled spectrum (bottom) and ^{1}H-coupled spectrum (top). In ^{31}P-NMR ^{1}H-decoupled, there are two wide doublets and a triplet around -21 to -22 ppm while in case of ^{1}H-coupled ^{31}P-NMR, the doublet around -11 ppm splits into a doublet of triplets.

Assessment of 5′-ADP and ATP ^{31}P-NMR spectra.

The reaction samples containing both ATP and ADP were assessed and interpreted based on the various peak locations. Usually, the multiplets at (chemical shift values) -6 and –11 ppm comprise of the terminal and α signals of both ADP and ATP. Therefore, the terminal resonance at –6 ppm was observed and chosen for the quantitative analyses because the α resonance is affected by NOE. It should be noted that the signal at/around -6 ppm is given by both ADP and ATP. However, the β resonance at –21 ppm comes

specifically from ATP. Therefore, comparing the integral at –6 ppm and relating it to the signal at –21 ppm helped in the assessment and differentiation of ATP from ADP. This method of the assessment of ADP and ATP has been discussed previously [2].

Figure S4. ^{31}P-NMR (^{1}H-coupled) spectrum of Sample AD-1.

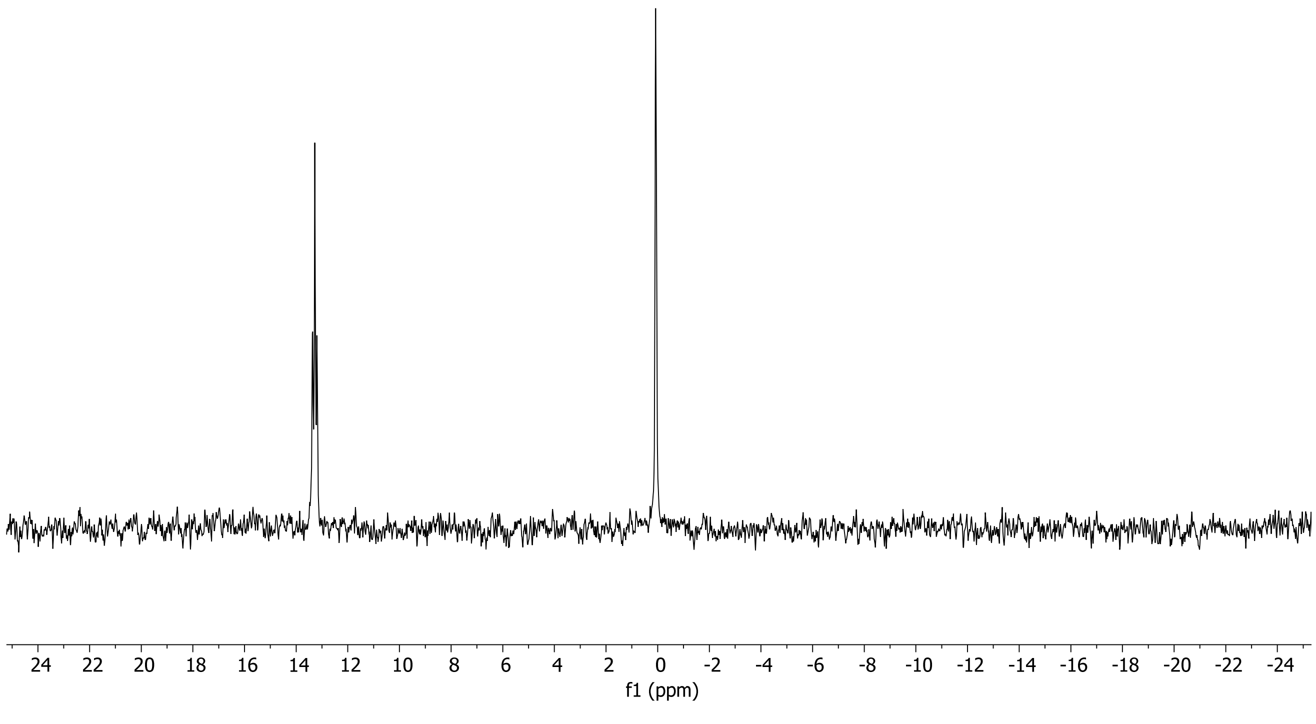

Figure S5. ^{31}P-NMR (^{1}H-coupled) spectrum of sample AD-2.

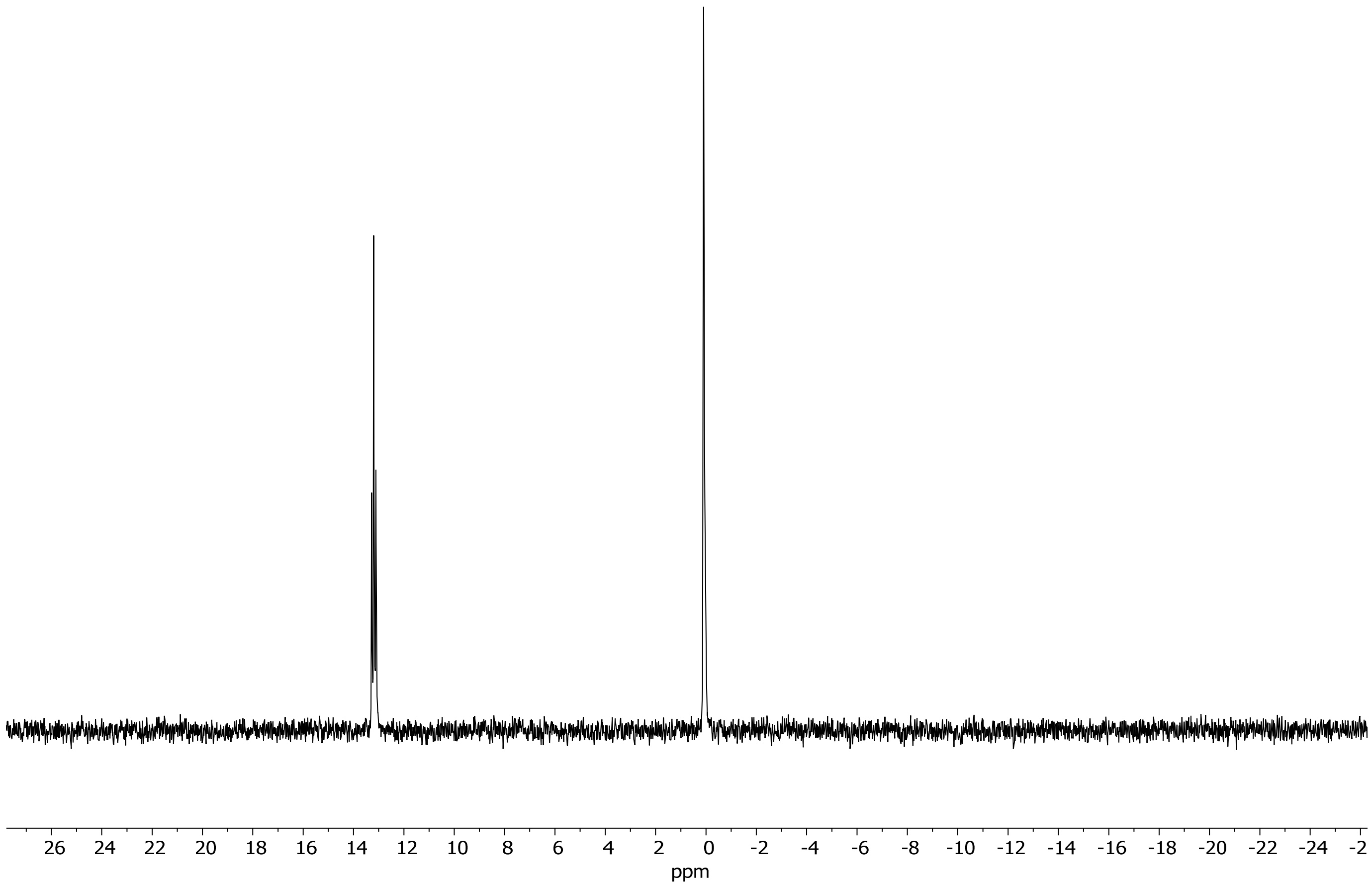

Figure S6. ^{31}P-NMR (^{1}H-coupled) spectrum of Sample AD-3.

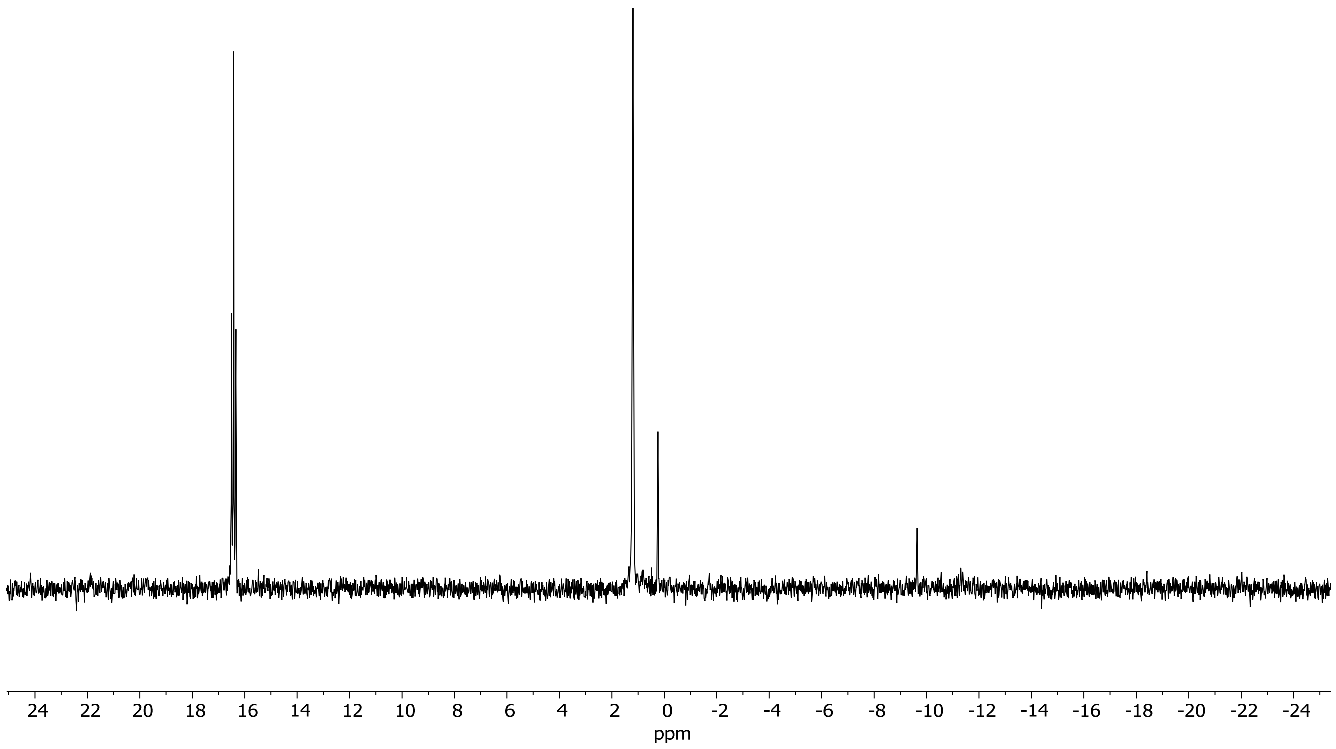

Figure S7. ^{31}P-NMR (^{1}H-coupled) spectrum of Sample AD-4.

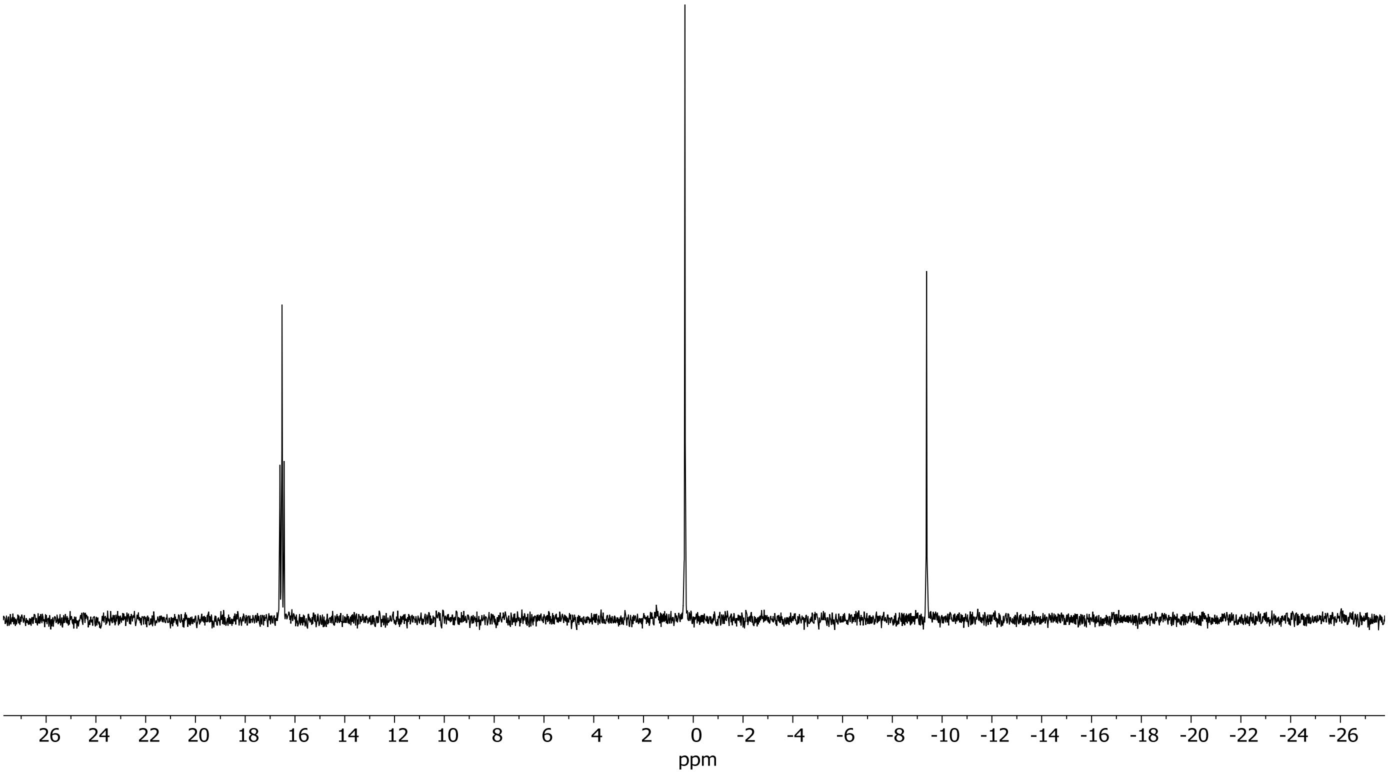

Figure S8. ^{31}P-NMR (^{1}H-coupled) spectrum of Sample AD-5.

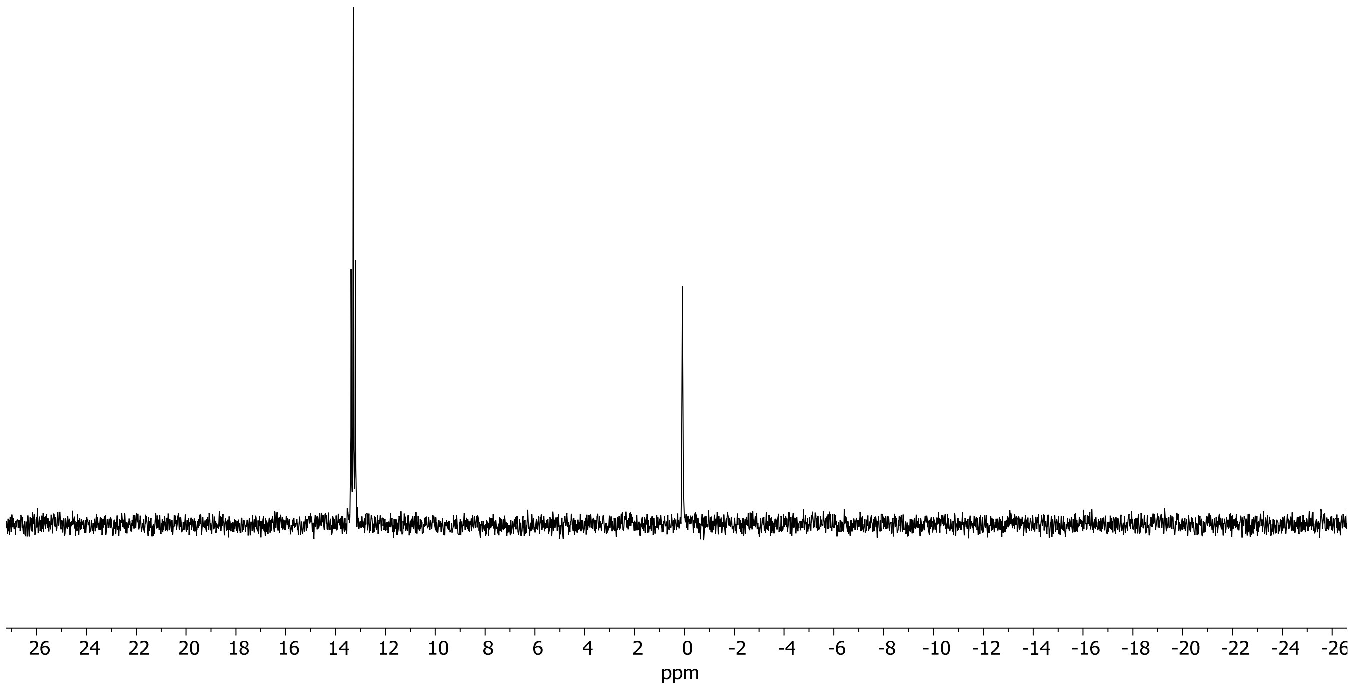

Figure S9. ^{31}P-NMR (^{1}H-coupled) spectrum of Sample AD-6.

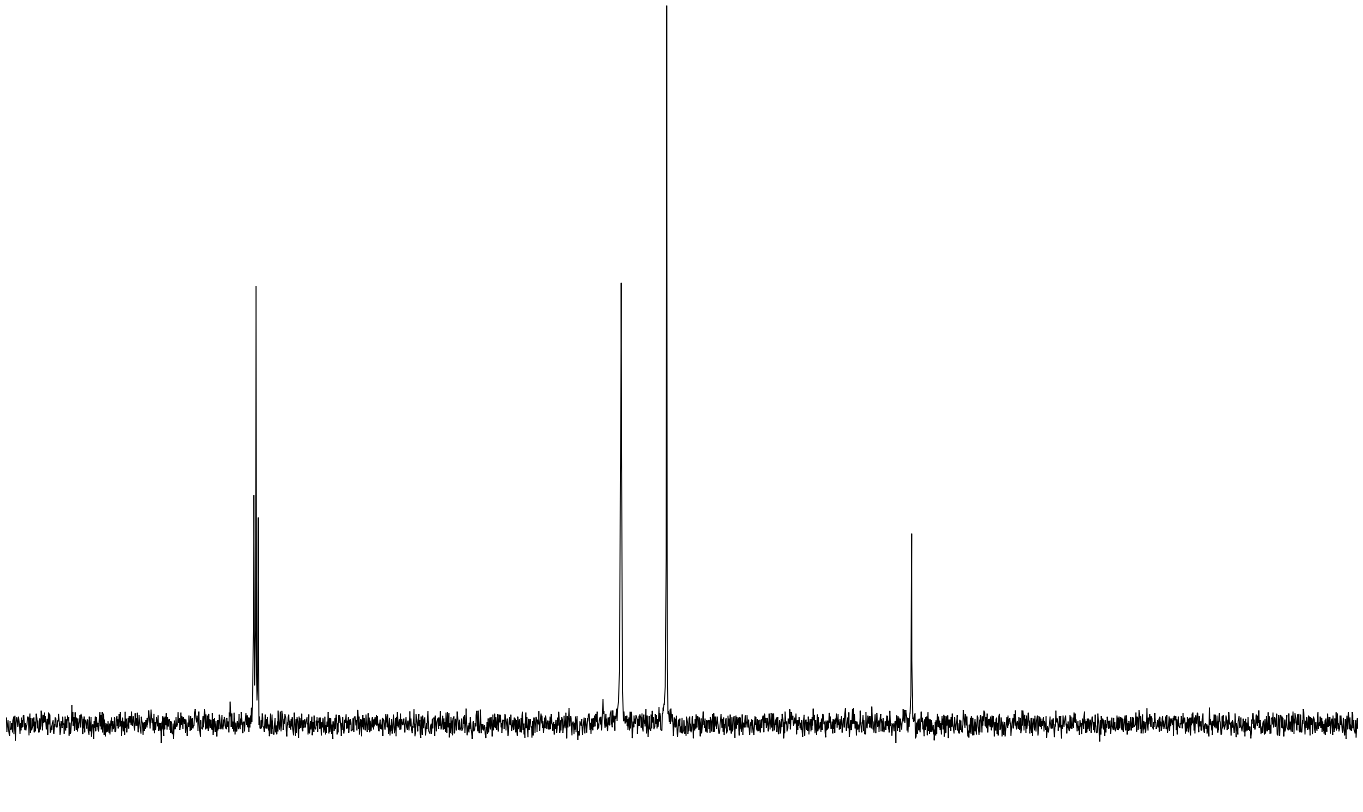

Figure S10. ^{31}P-NMR (^{1}H-coupled) spectrum of Sample AD-7.

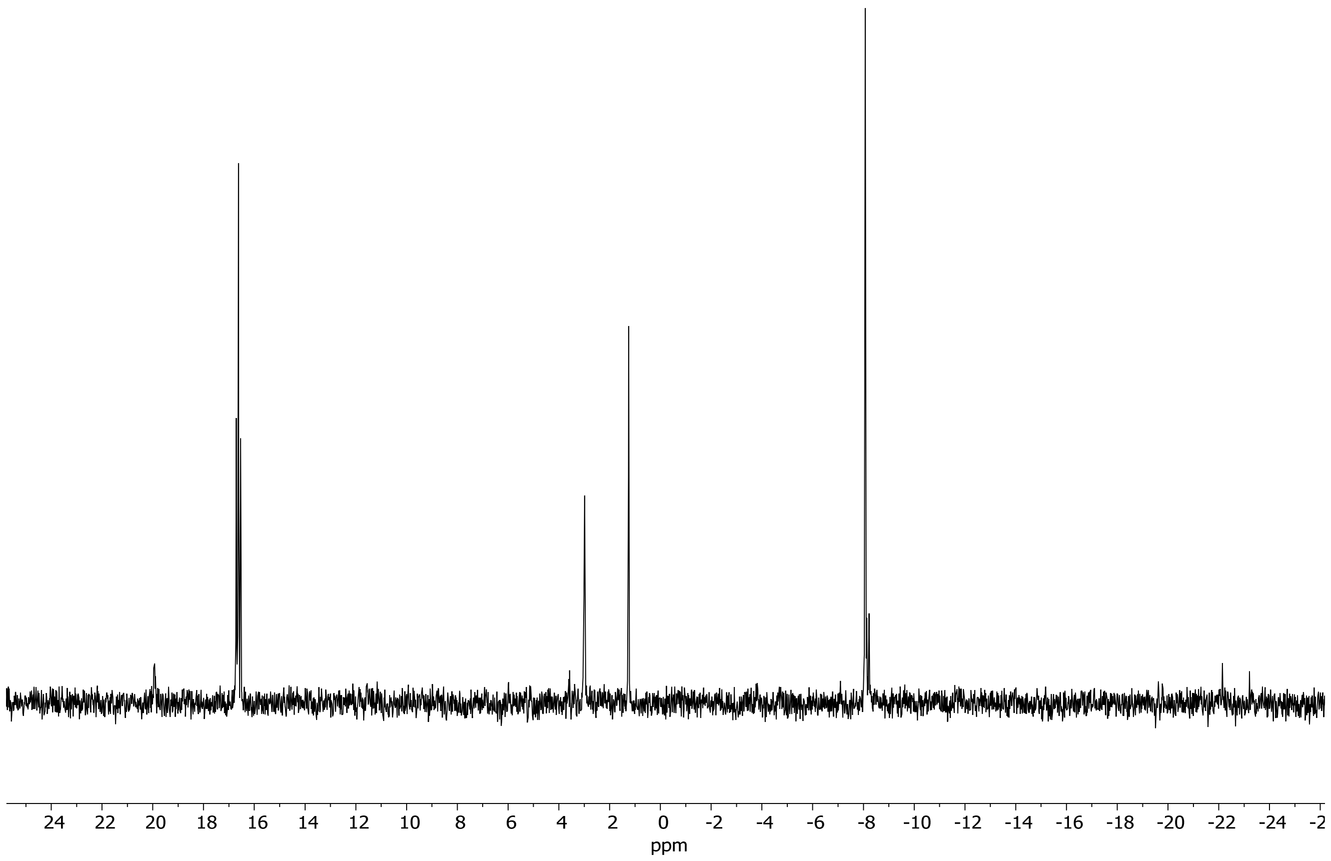

Figure S11. ^{31}P-NMR (^{1}H-coupled) spectrum of Sample AD-8.

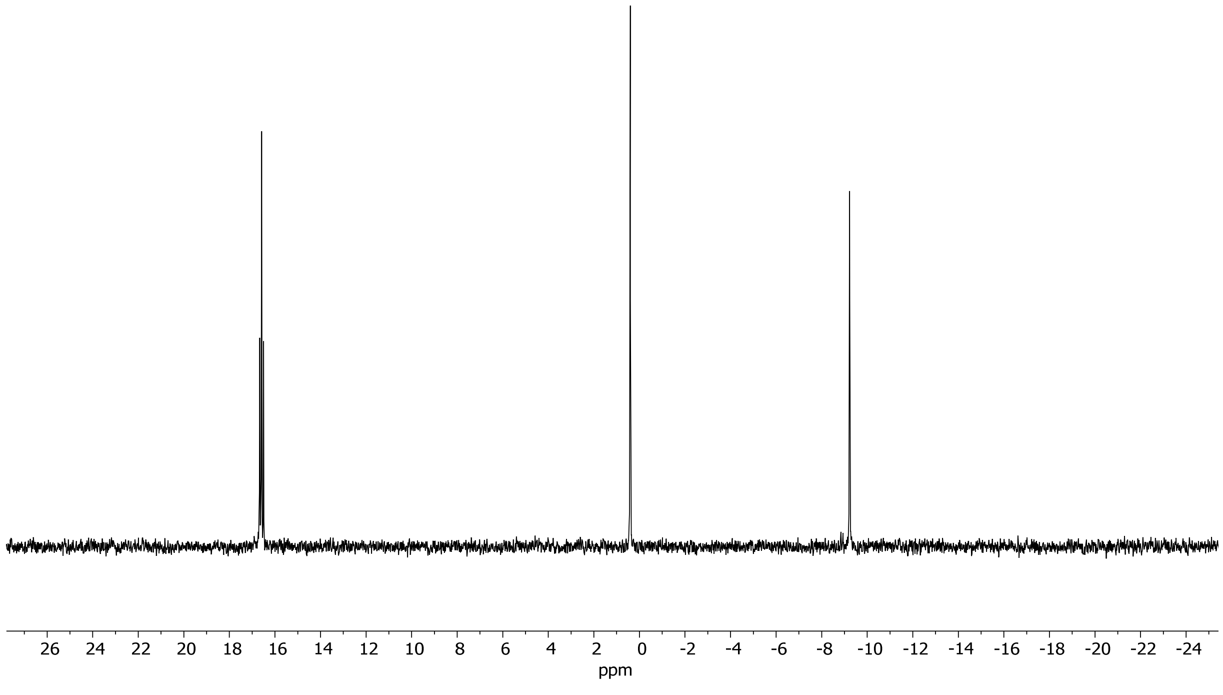

Figure S12. ^{31}P-NMR (^{1}H-coupled) spectrum of Sample AD-9.

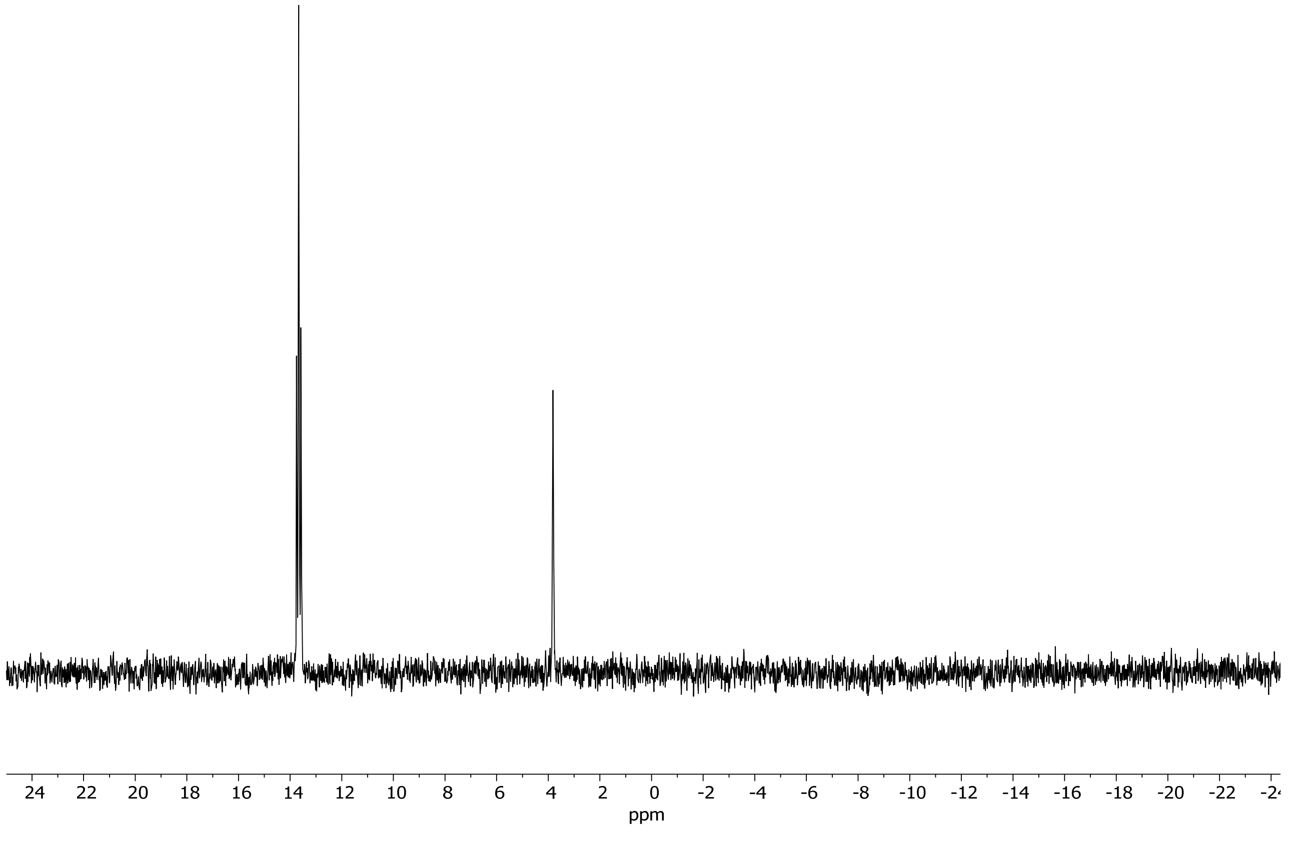

Figure S13. ^{31}P-NMR (^{1}H-coupled) spectrum of Sample AD-10.

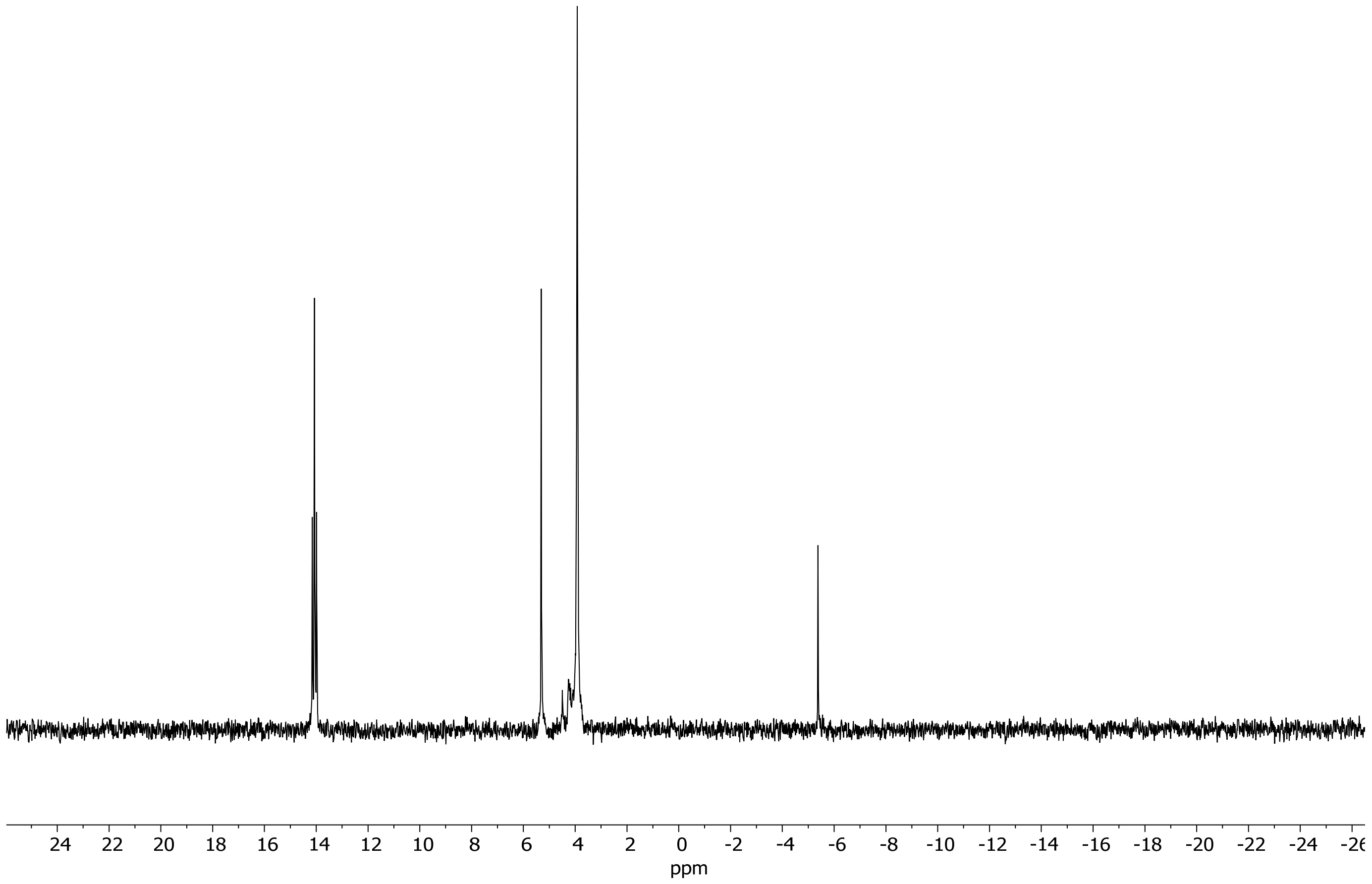

Figure S 14. ^{31}P-NMR (^{1}H-coupled) spectrum of Sample AD-11.

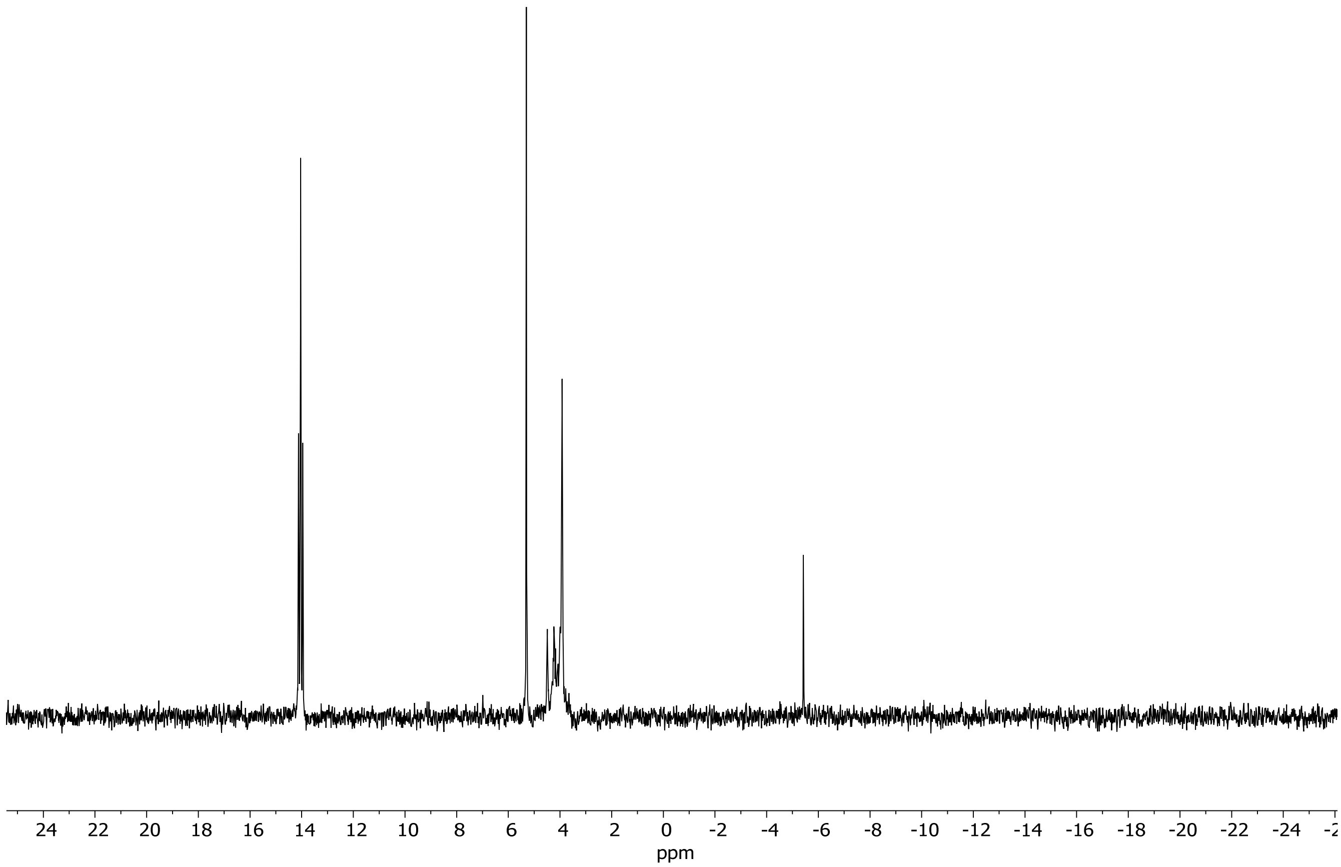

Figure S 15. ^{31}P-NMR (^{1}H-coupled) spectrum of Sample AD-12.

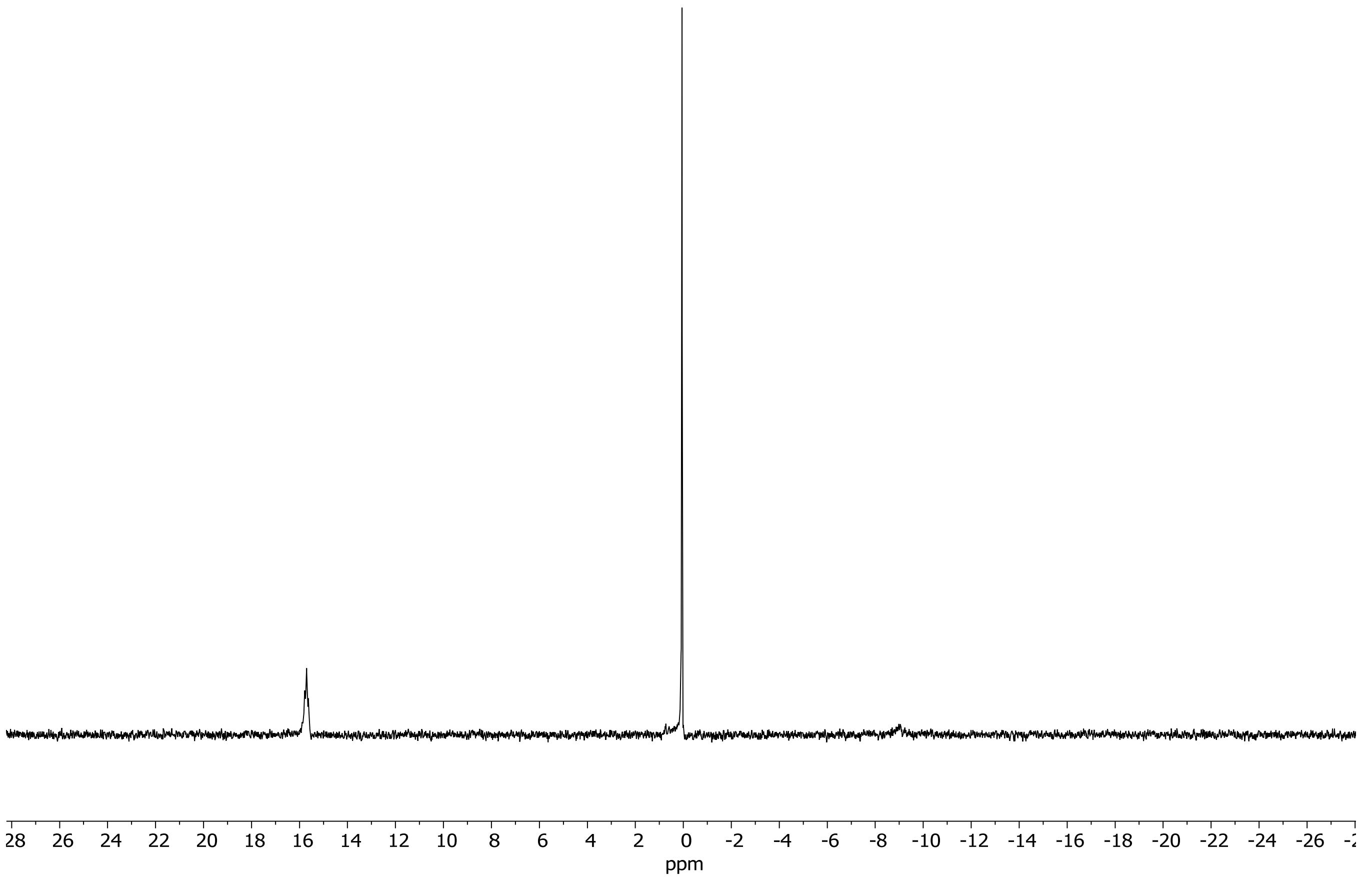
28 26 24 22 20 18 16 14 12 10 8 6 4 2 0 -2 -4 -6 -8 -10 -12 -14 -16 -18 -20 -22 -24 -26
ppm

Figure S 16. ^{31}P-NMR (^{1}H-coupled) spectrum of Sample AD-13.

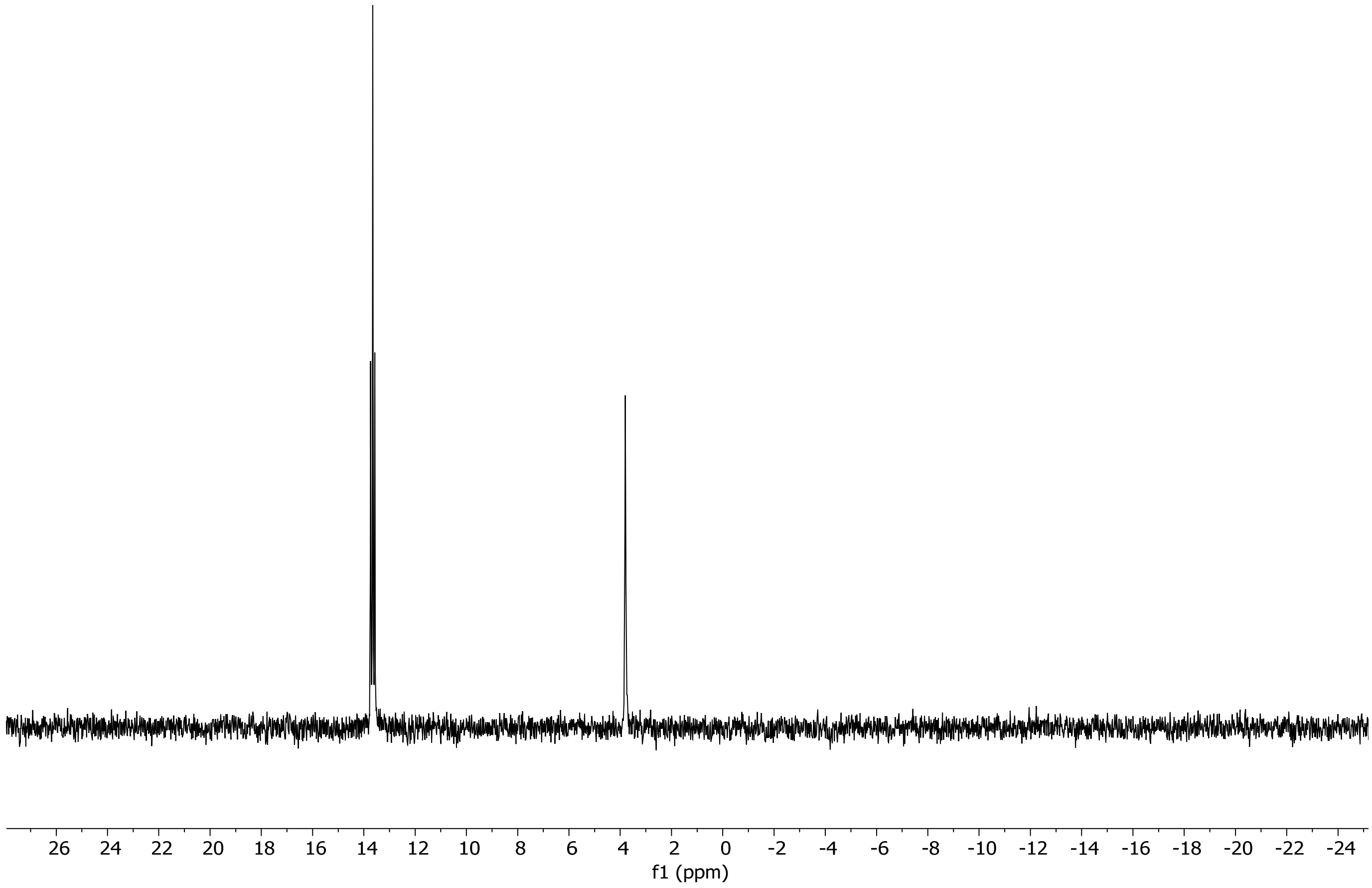

Figure S 17. ^{31}P-NMR (^{1}H-coupled) spectrum of Sample AD-14.

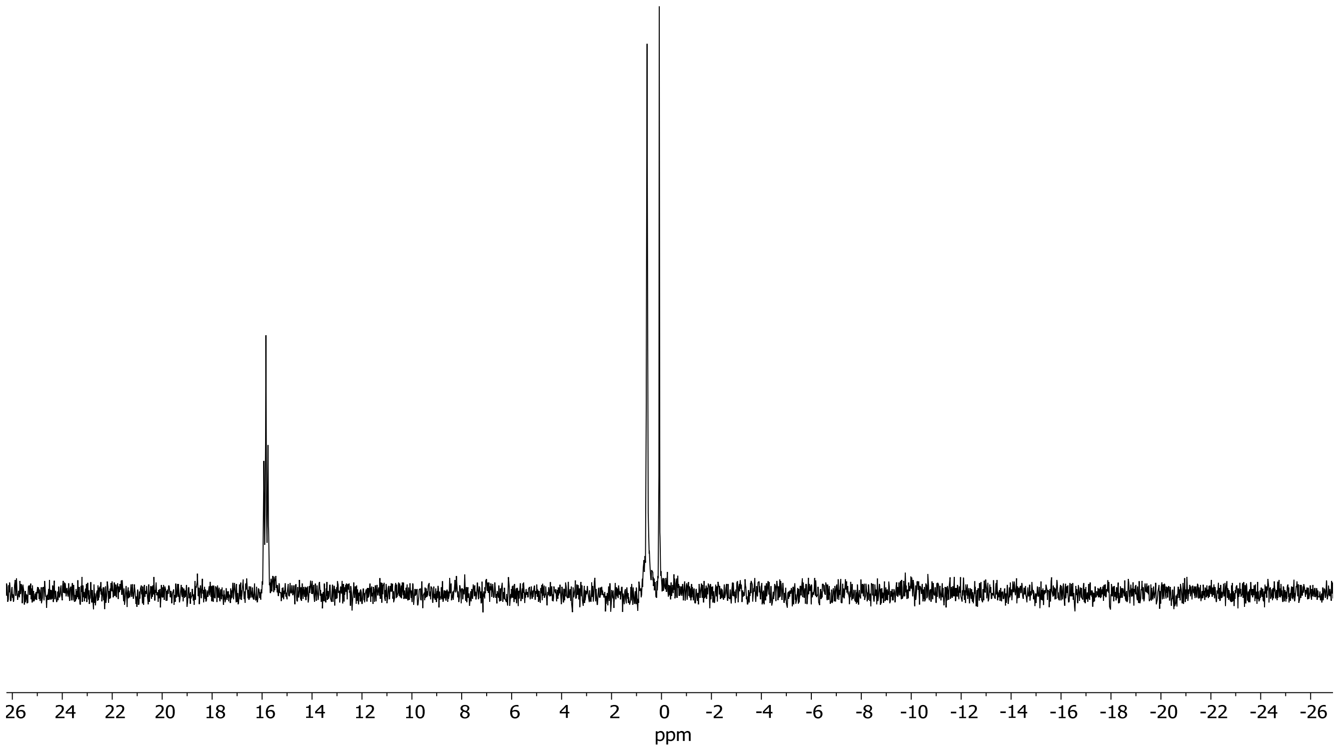

Figure S 18. ^{31}P-NMR (^{1}H-coupled) spectrum of Sample AD-15.

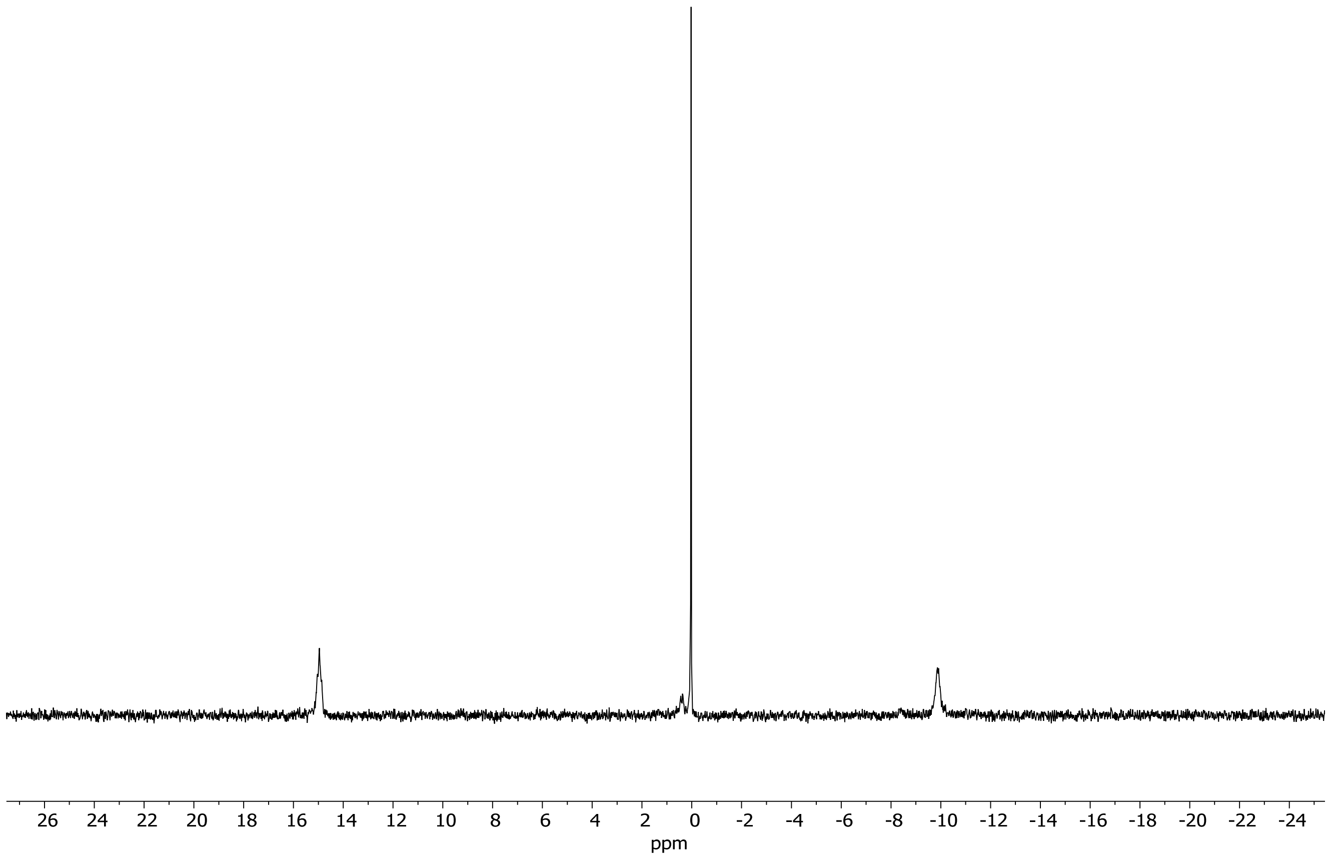

Figure S 19. ^{31}P-NMR (^{1}H-coupled) spectrum of Sample AD-16.

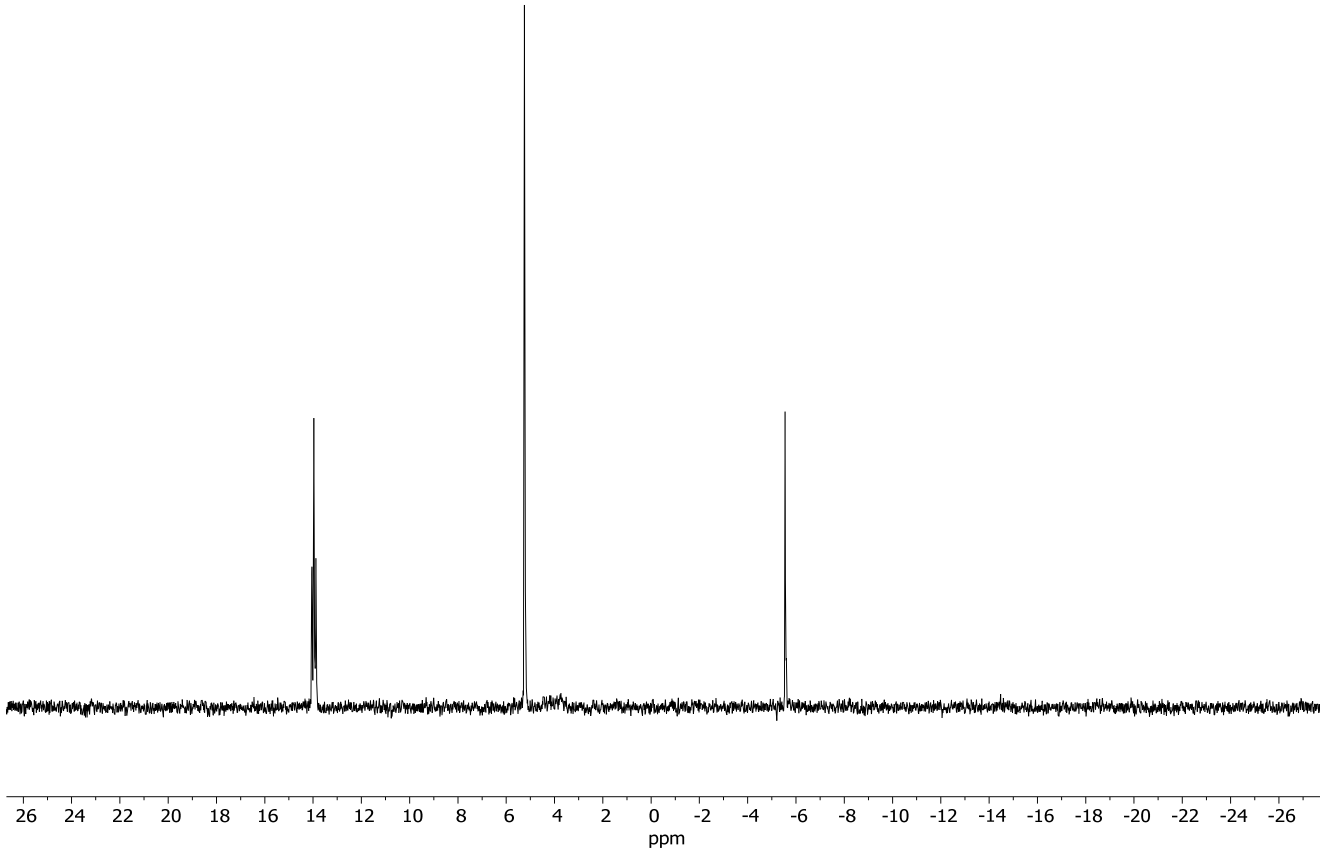

Figure S 20. ^{31}P-NMR (^{1}H-coupled) spectrum of Sample AD-17.

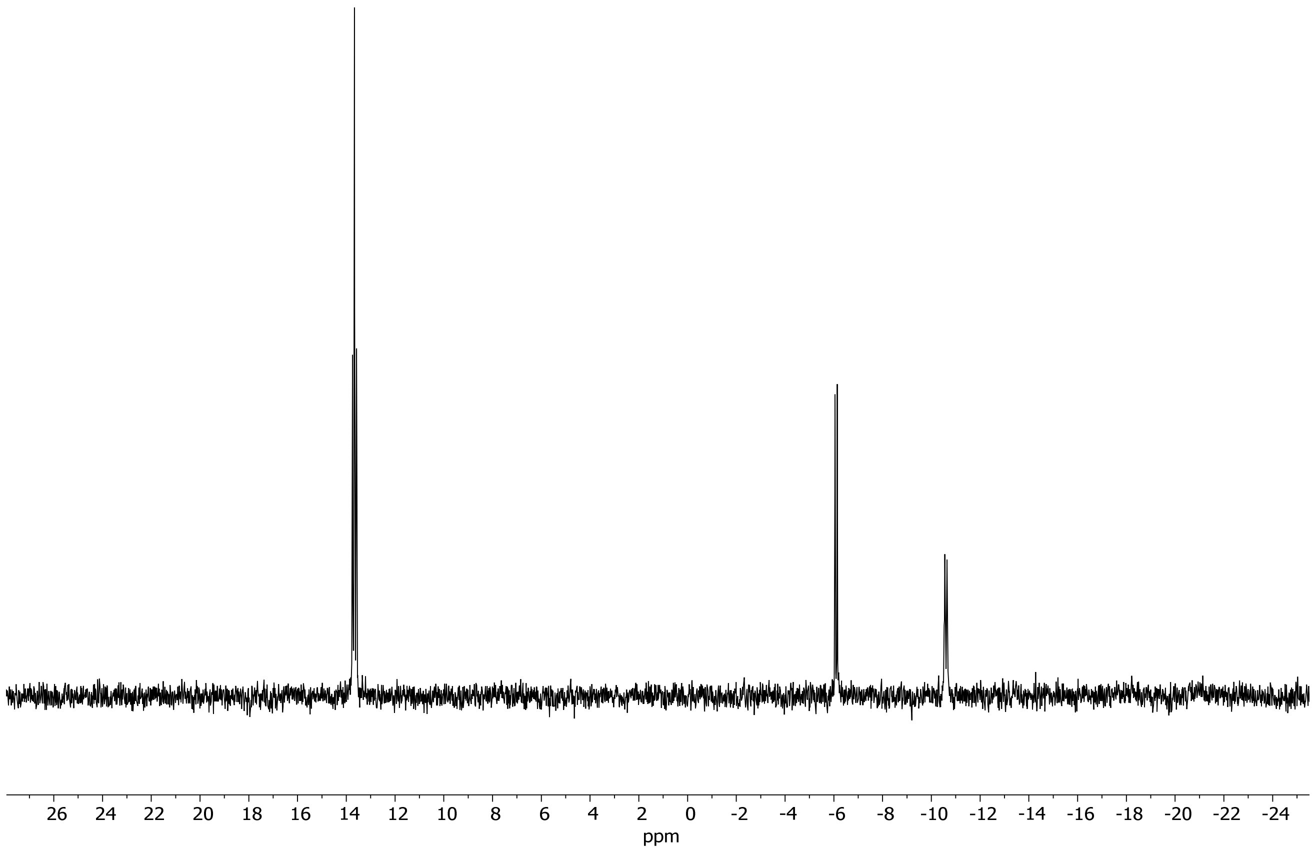

Figure S 21. ^{31}P-NMR (^{1}H-coupled) spectrum of Sample AD-18.

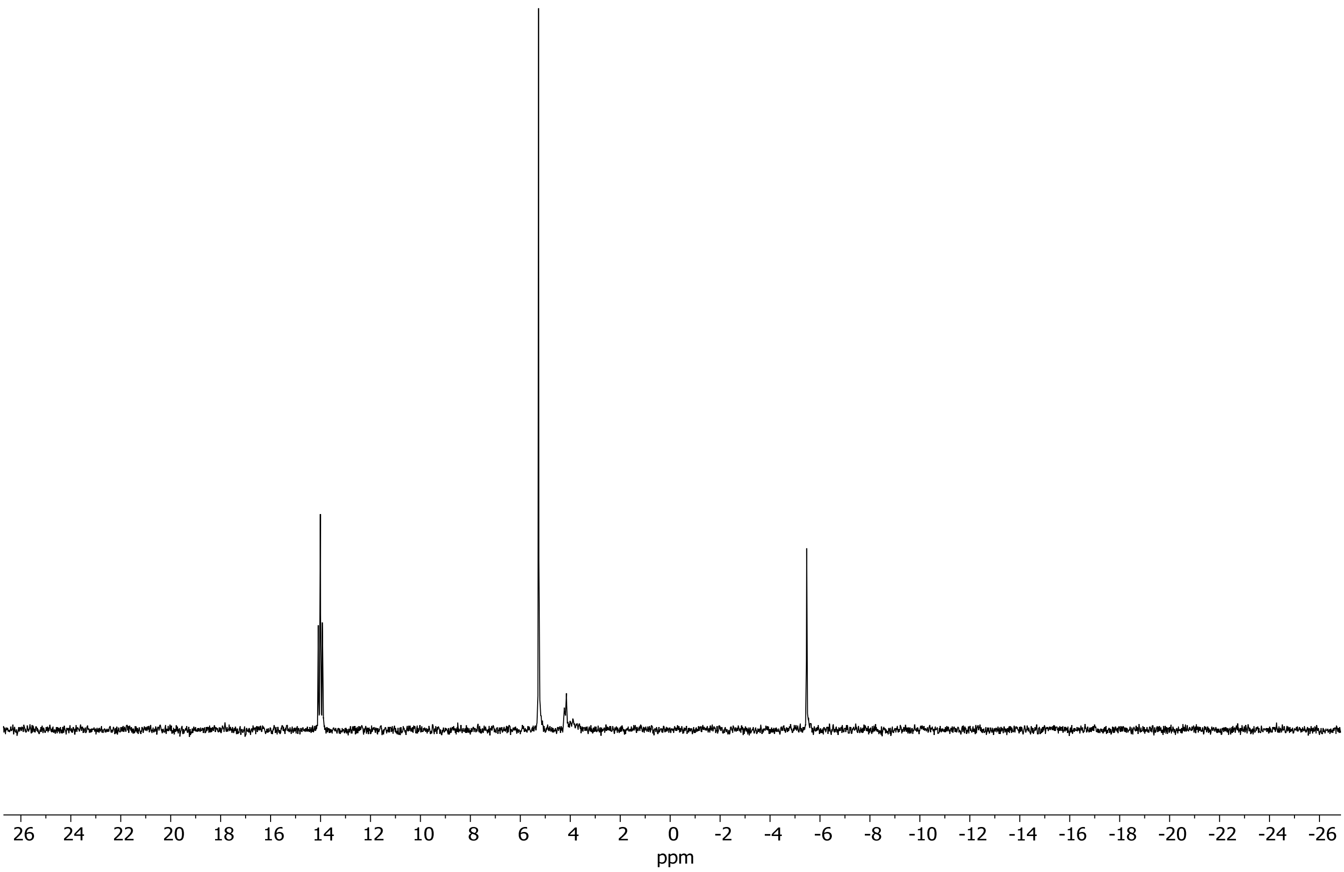

Figure S 22. ^{31}P-NMR (^{1}H-coupled) spectrum of Sample AD-19.

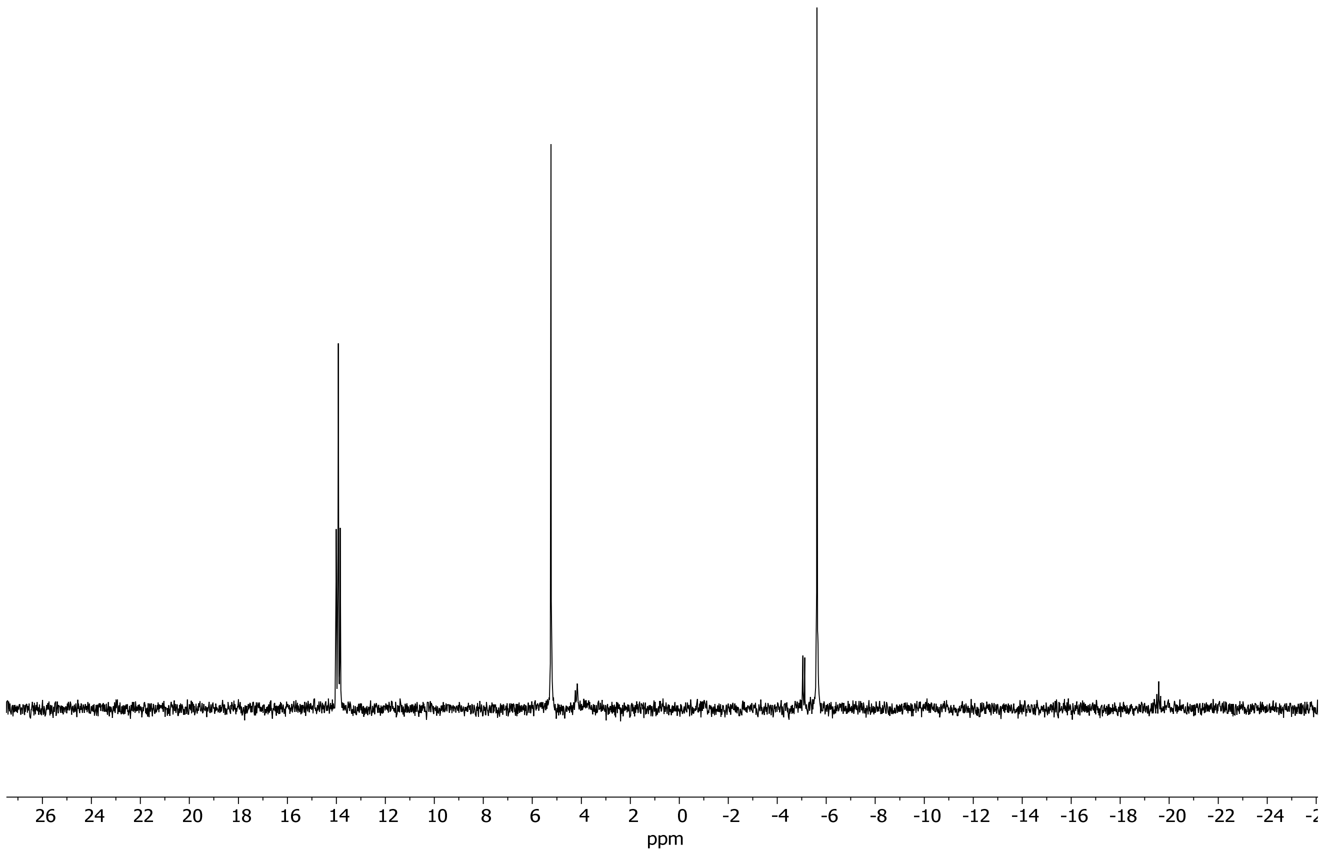

Figure S 23. ^{31}P-NMR (^{1}H-coupled) spectrum of Sample AD-20.

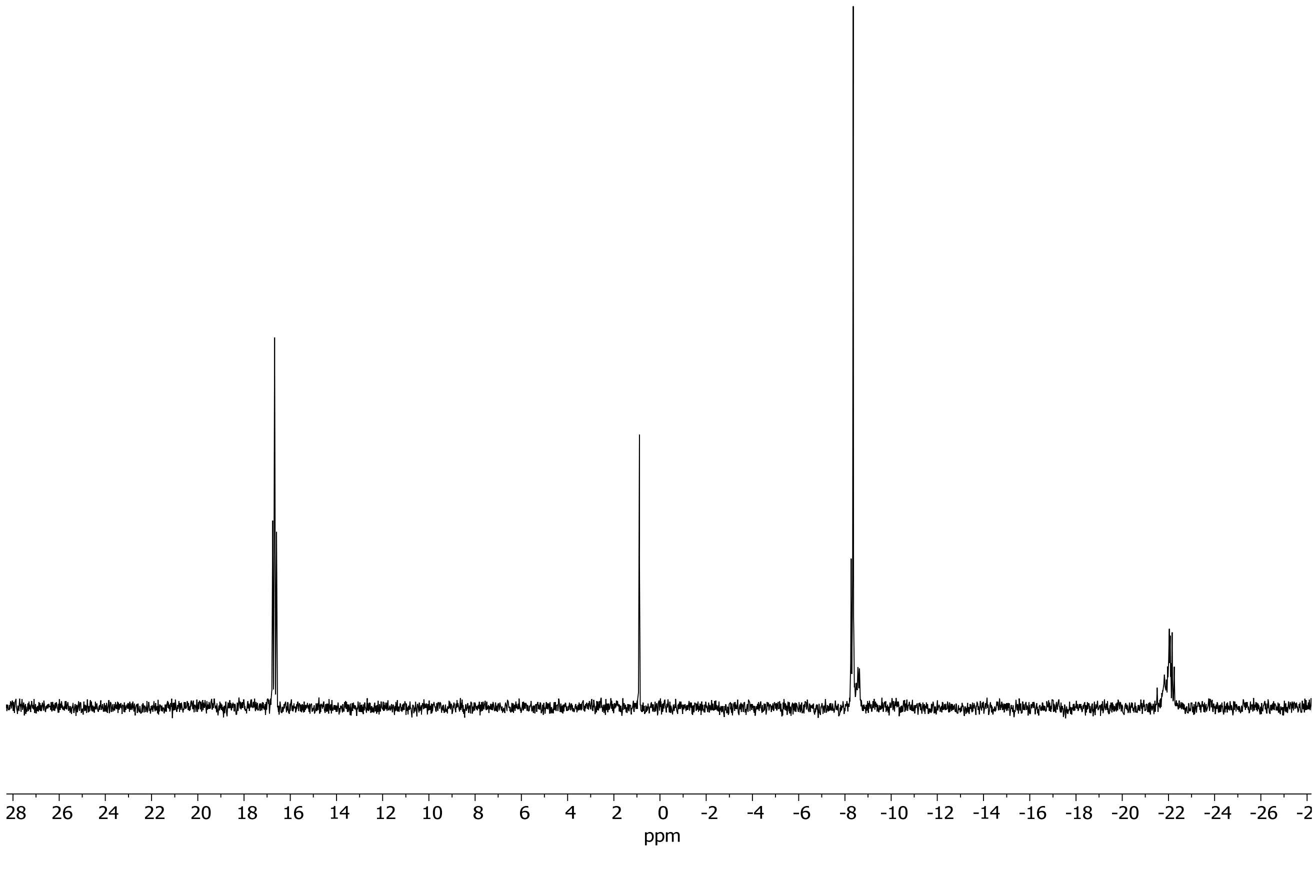

Figure S 24. ^{31}P-NMR (^{1}H-coupled) spectrum of Sample AD-21.

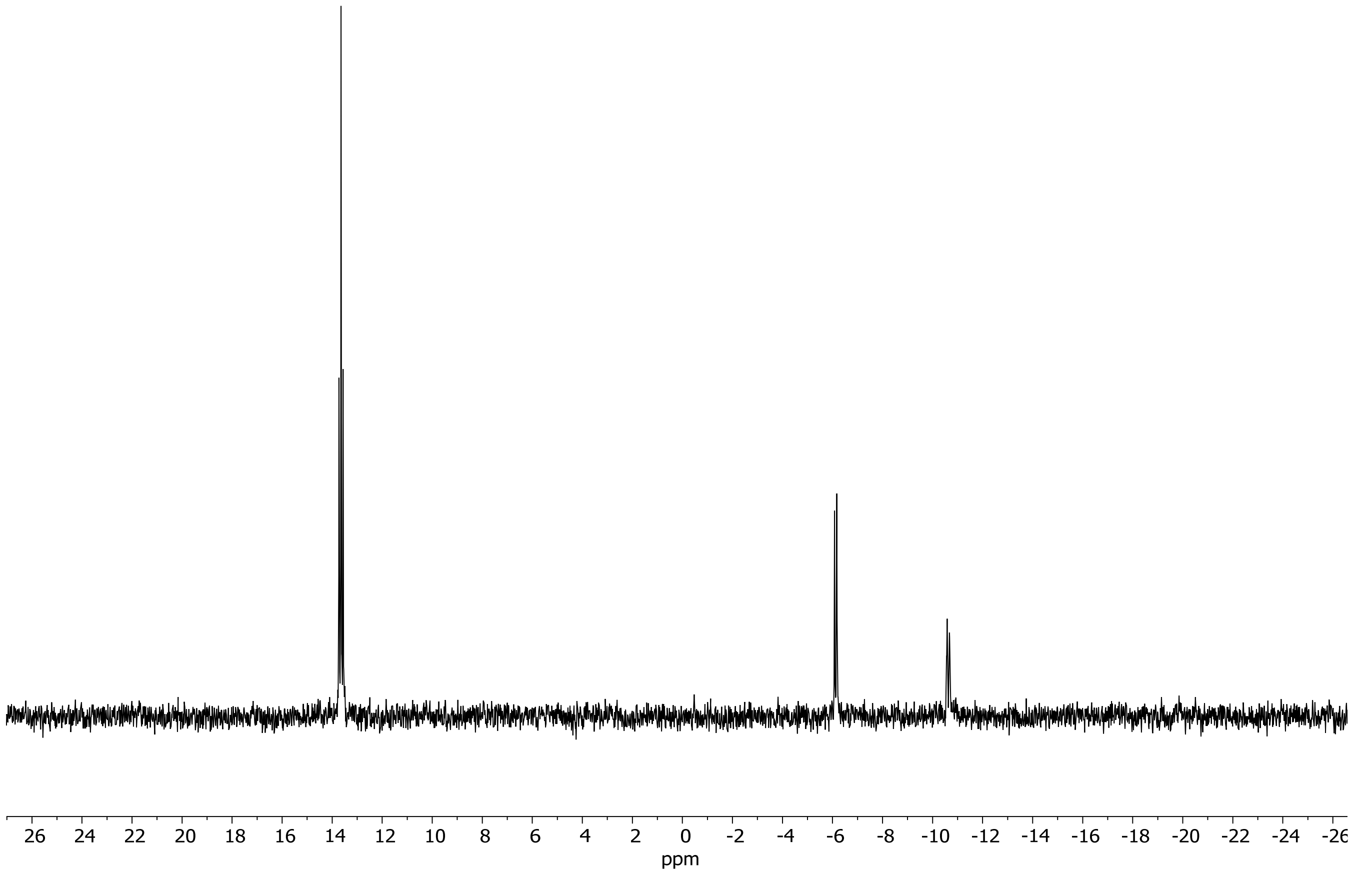

Figure S 25. ^{31}P-NMR (^{1}H-coupled) spectrum of Sample AD-22.

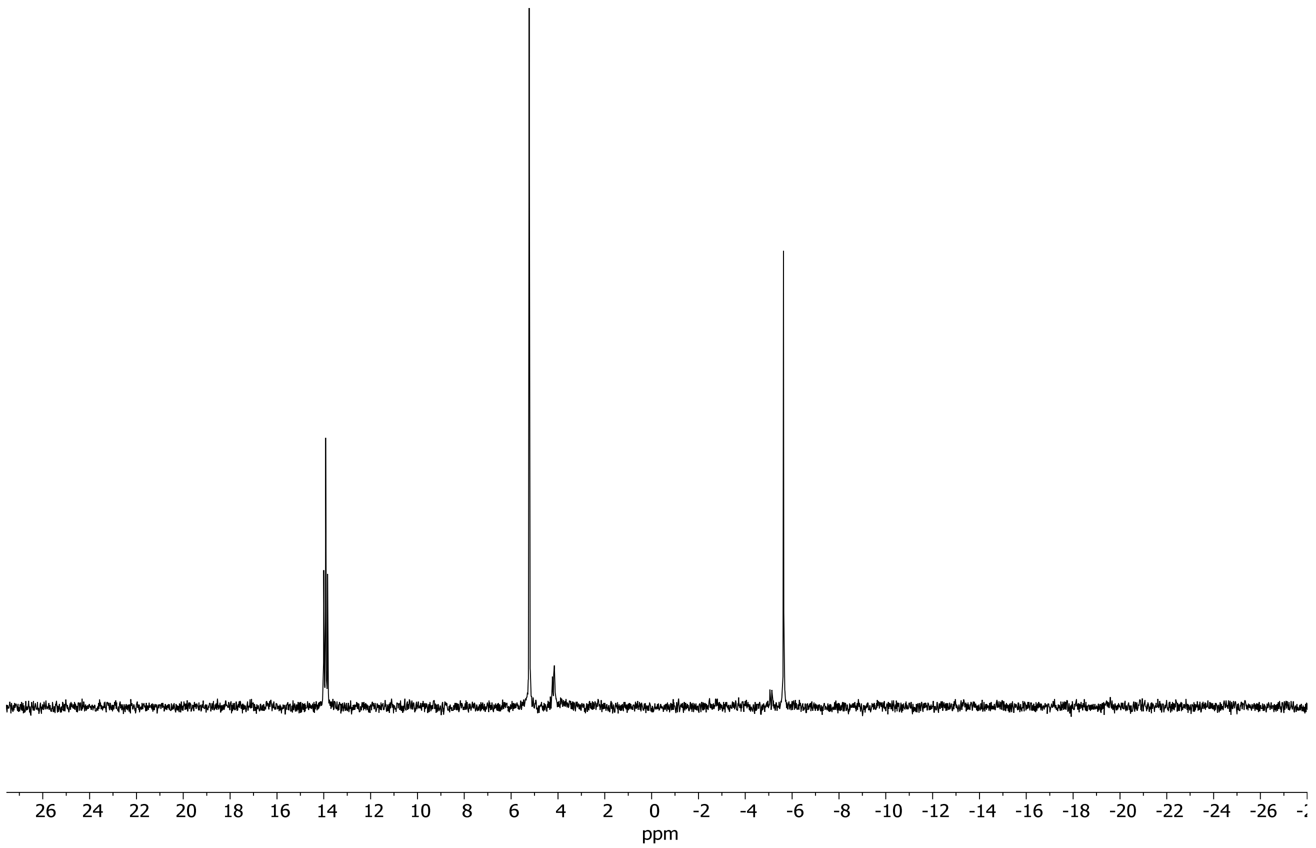

Figure S 26. ^{31}P-NMR (^{1}H-coupled) spectrum of Sample AD-23.

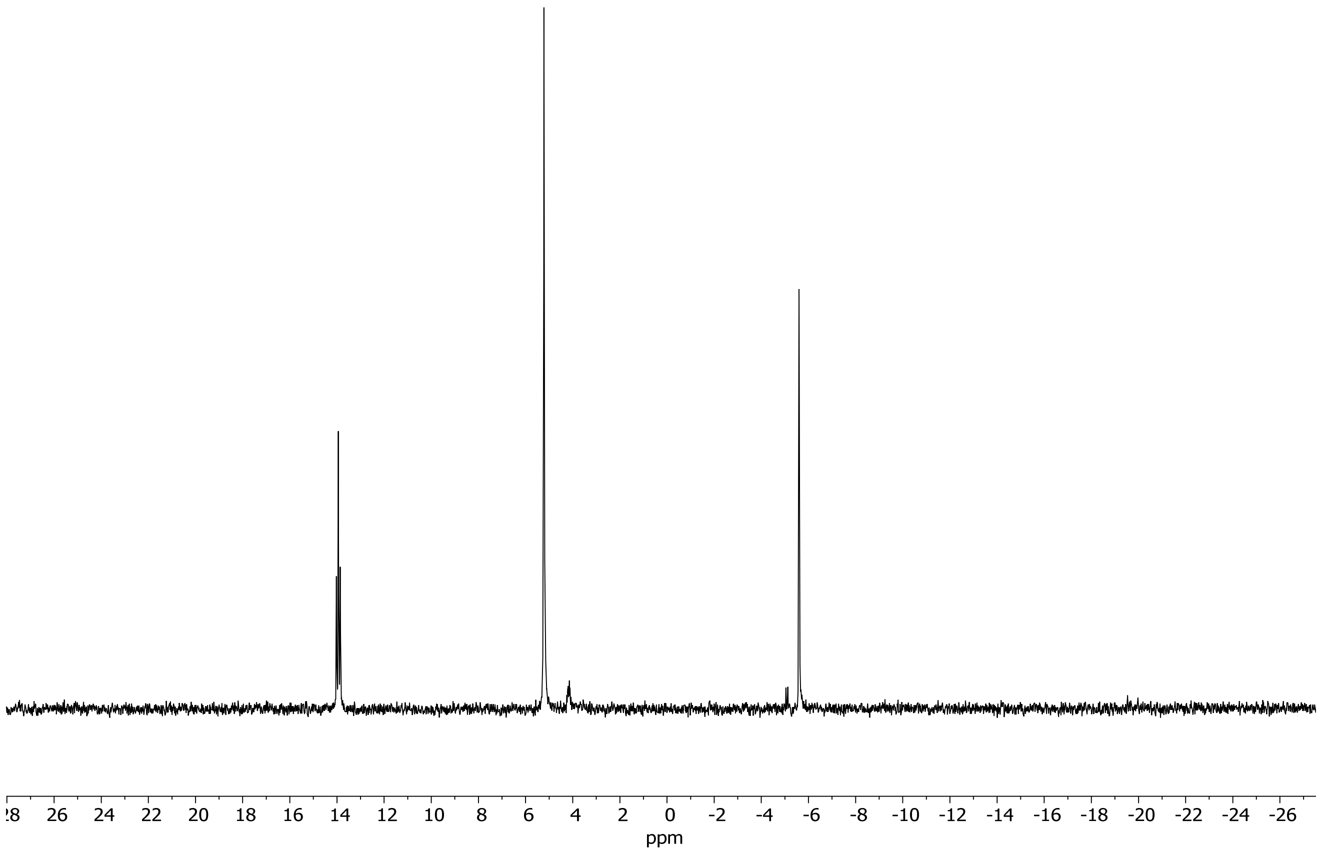

Figure S 27. ^{31}P-NMR (^{1}H-coupled) spectrum of Sample AD-24.

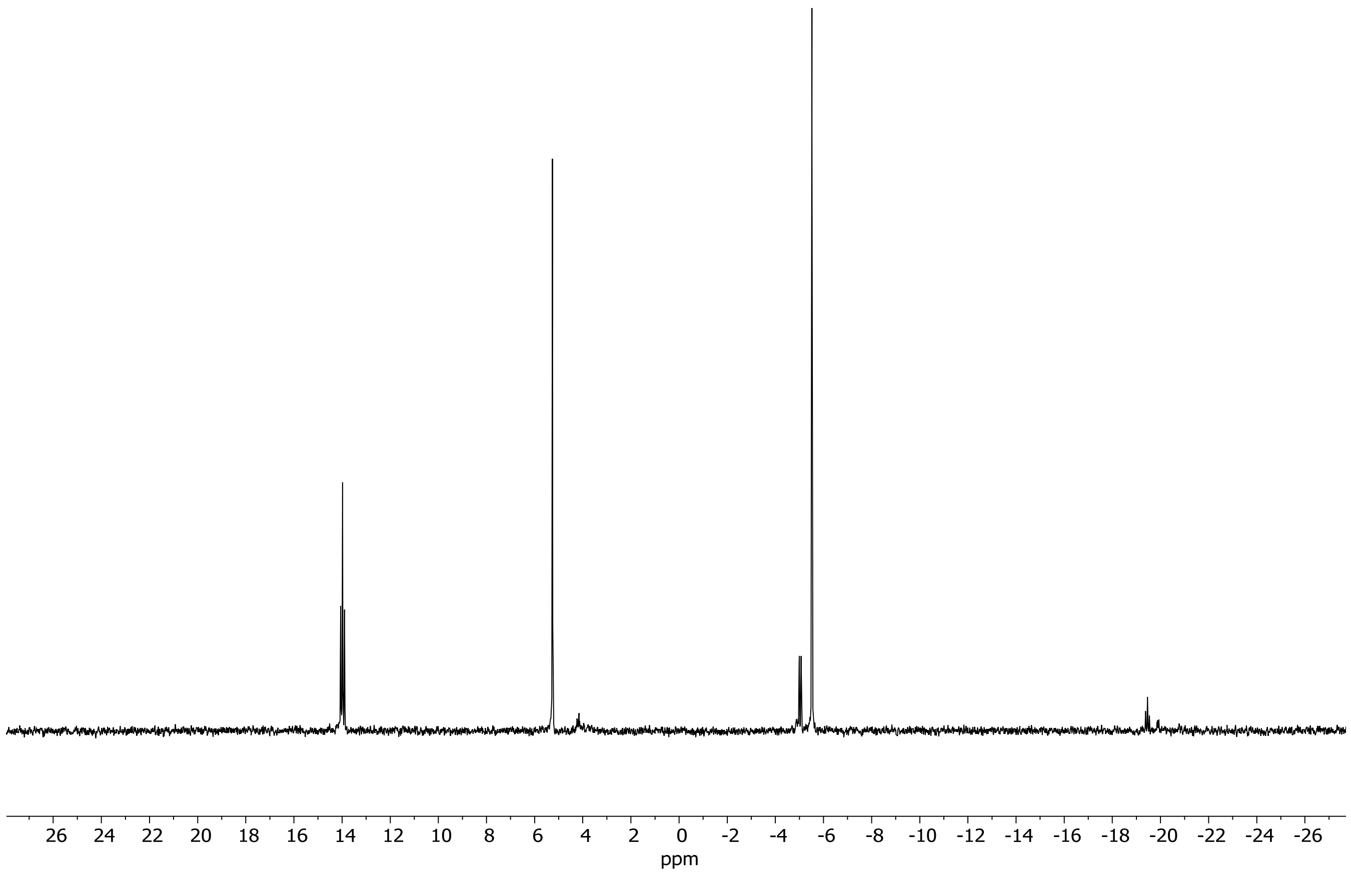

Figure S 28. ^{31}P-NMR (^{1}H-coupled) spectrum of Sample AD-25.

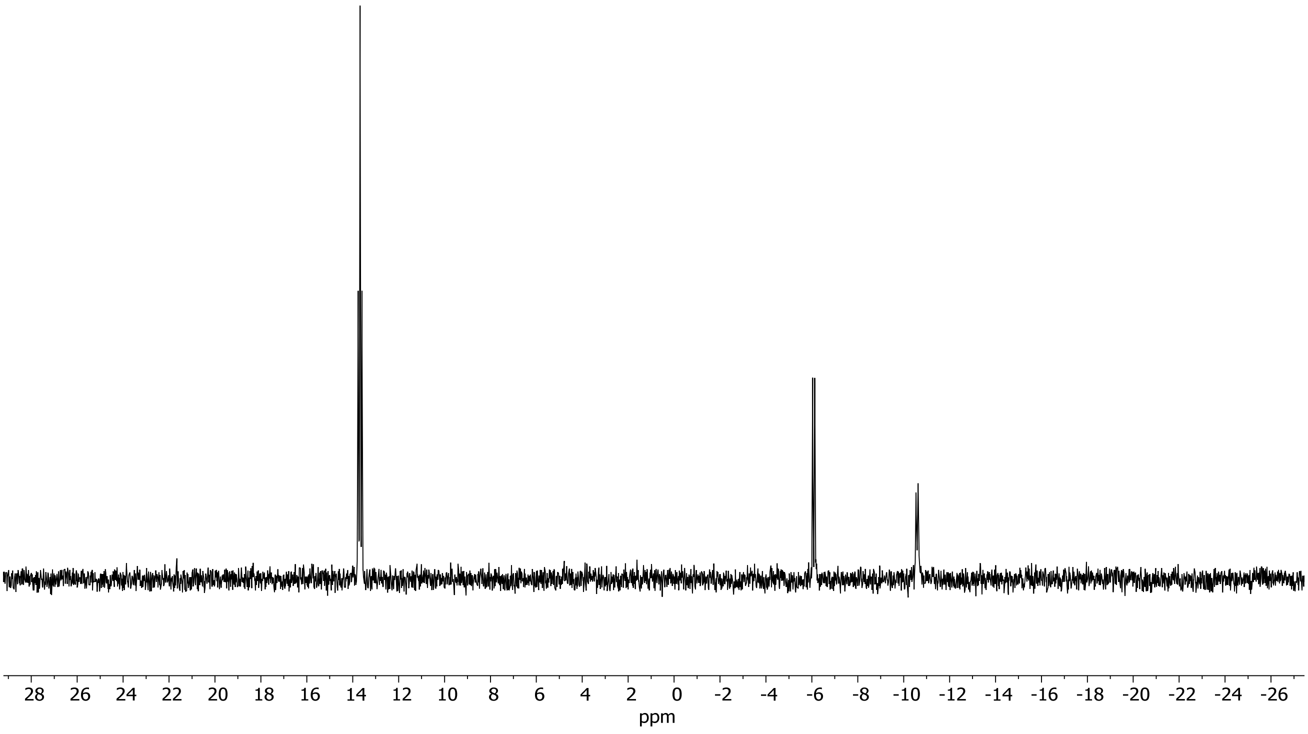

Figure S 29. ^{31}P-NMR (^{1}H-coupled) spectrum of Sample AD-26.

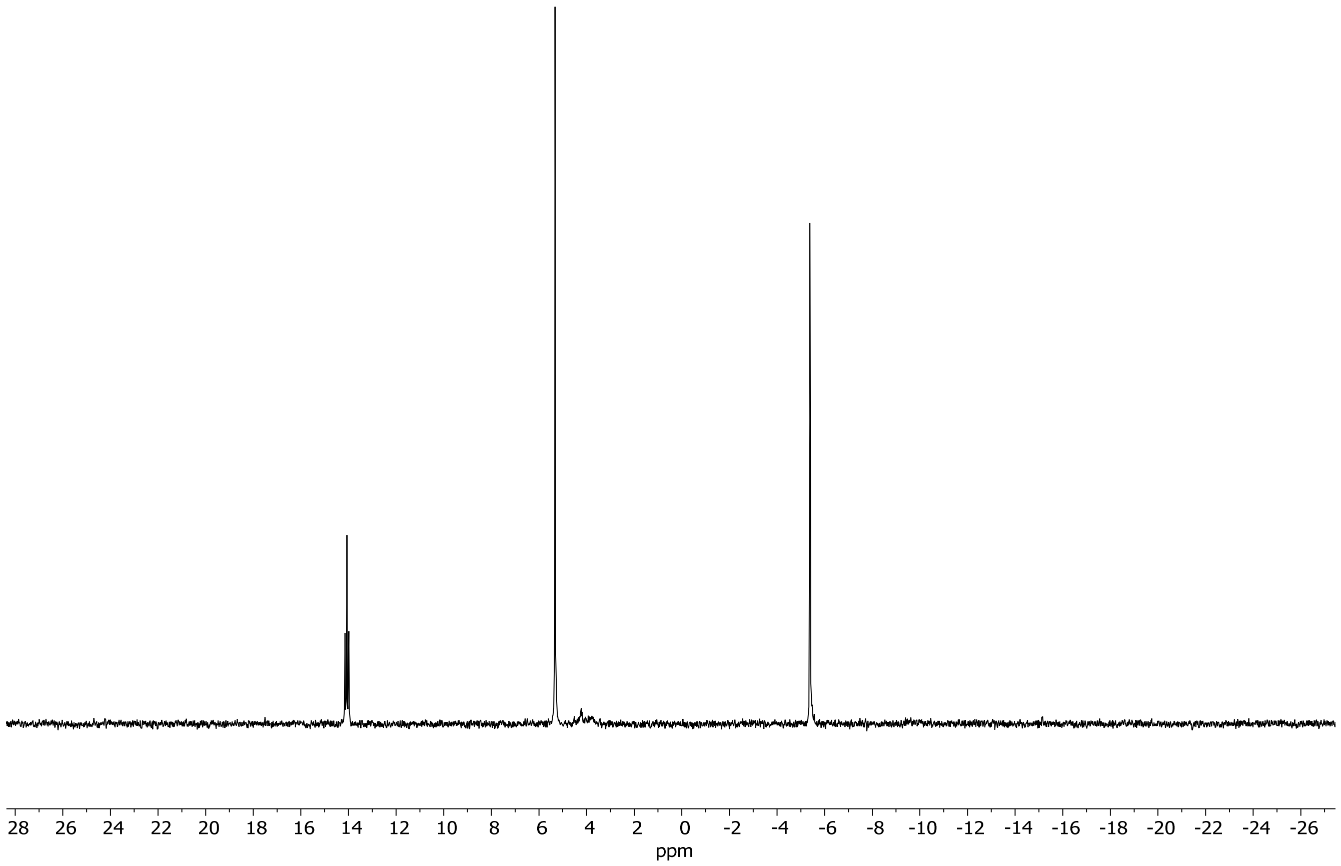

Figure S 30. ^{31}P-NMR (^{1}H-coupled) spectrum of Sample AD-27.

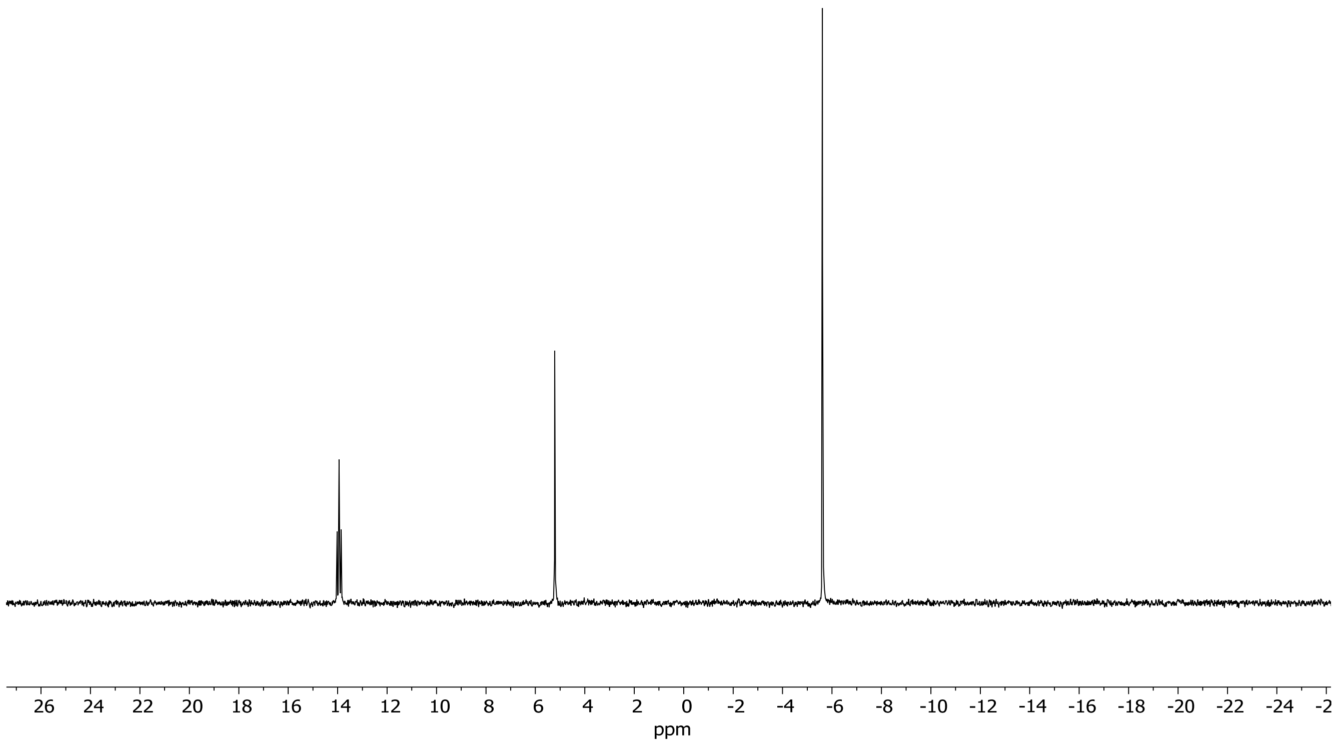
26 24 22 20 18 16 14 12 10 8 6 4 2 0 -2 -4 -6 -8 -10 -12 -14 -16 -18 -20 -22 -24 -2
ppm

Figure S 31. ^{31}P-NMR (^{1}H-coupled) 3spectrum of Sample AD-28.

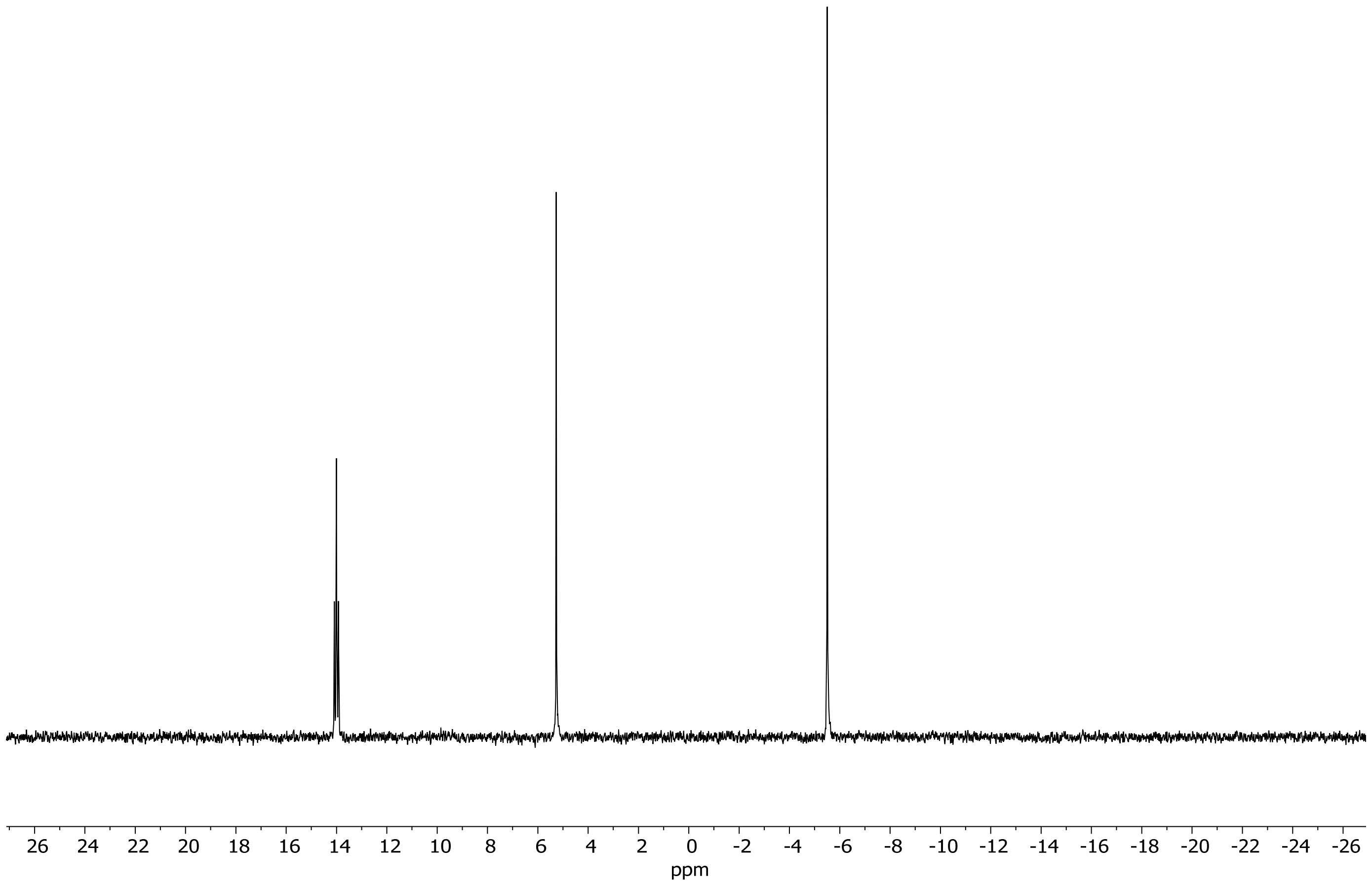

Figure S 32. ^{31}P-NMR (^{1}H-coupled) spectrum of Sample AD-29.

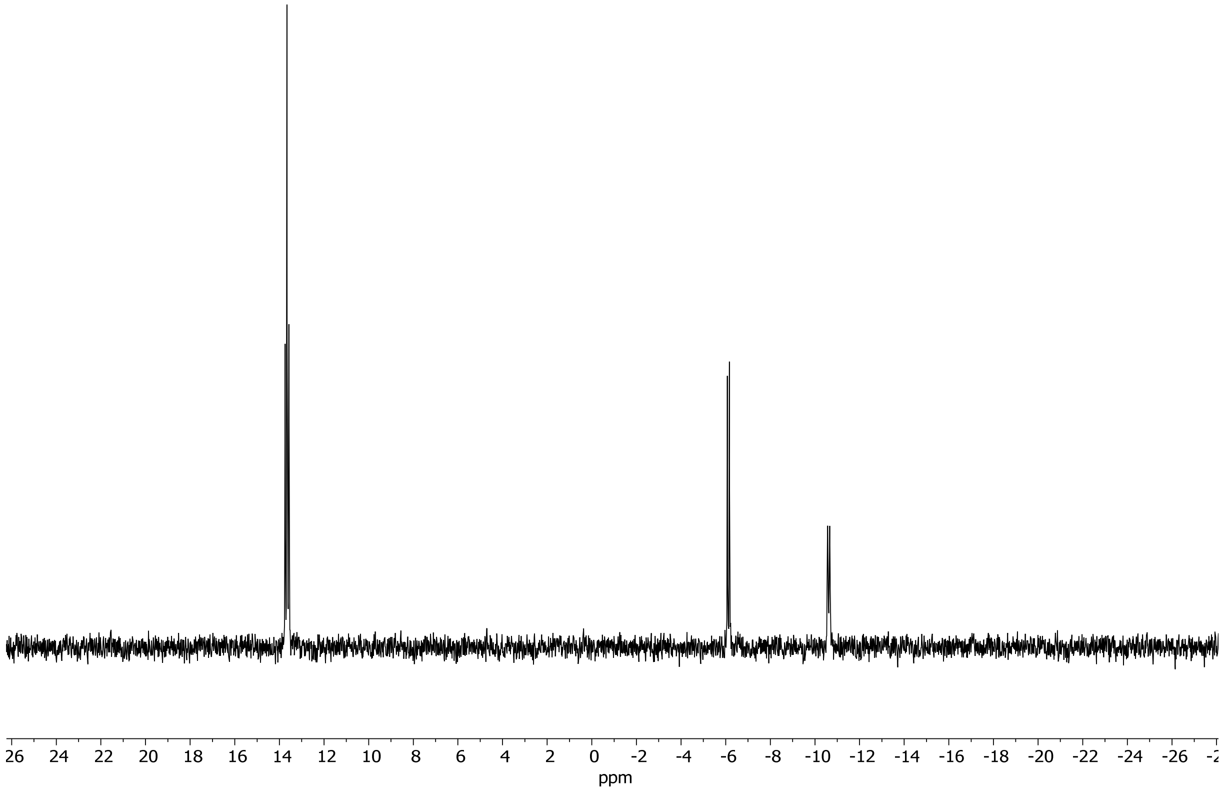

Figure S 33. ^{31}P-NMR (^{1}H-coupled) spectrum of Sample AD-30.

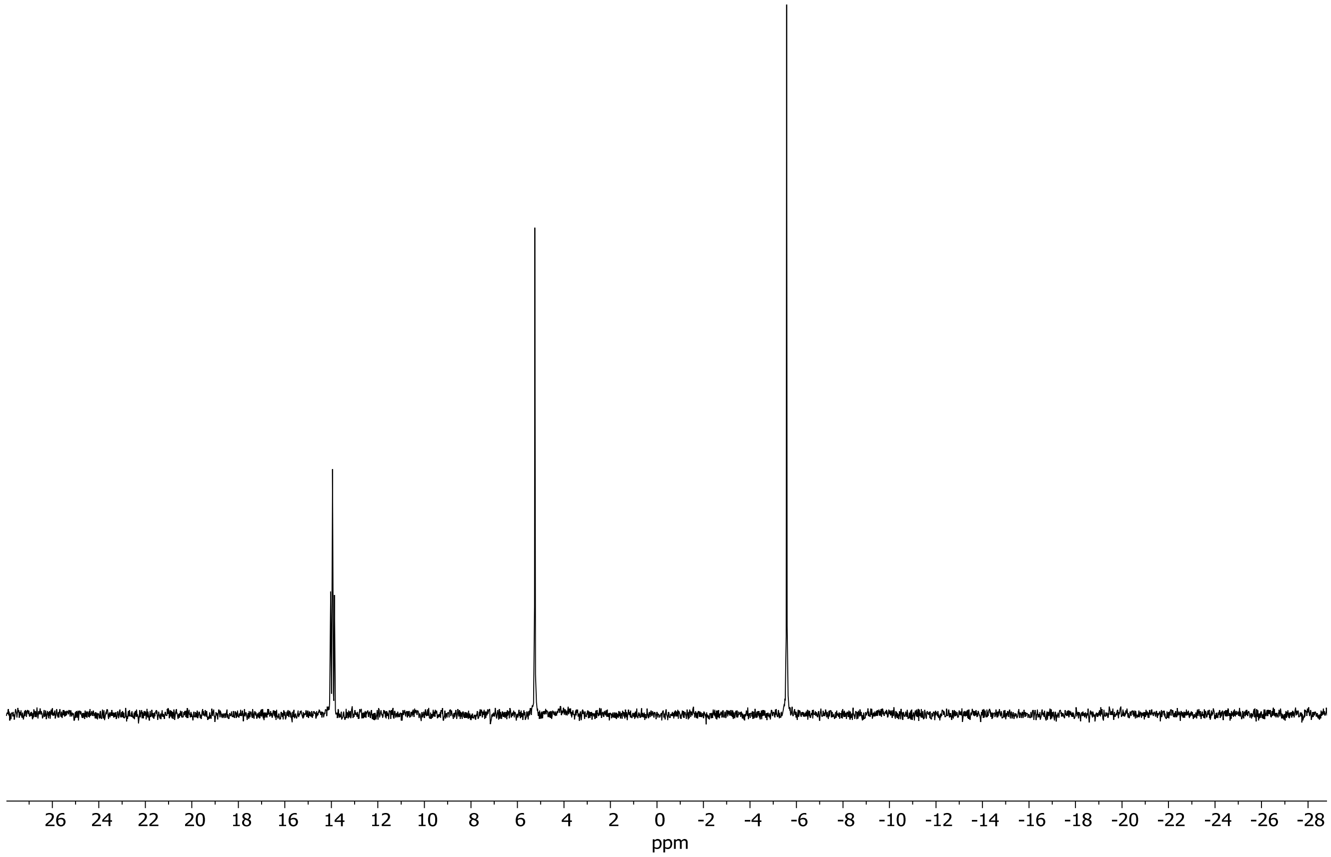

Figure S 34. ^{31}P-NMR (^{1}H-coupled) spectrum of Sample AD-31.

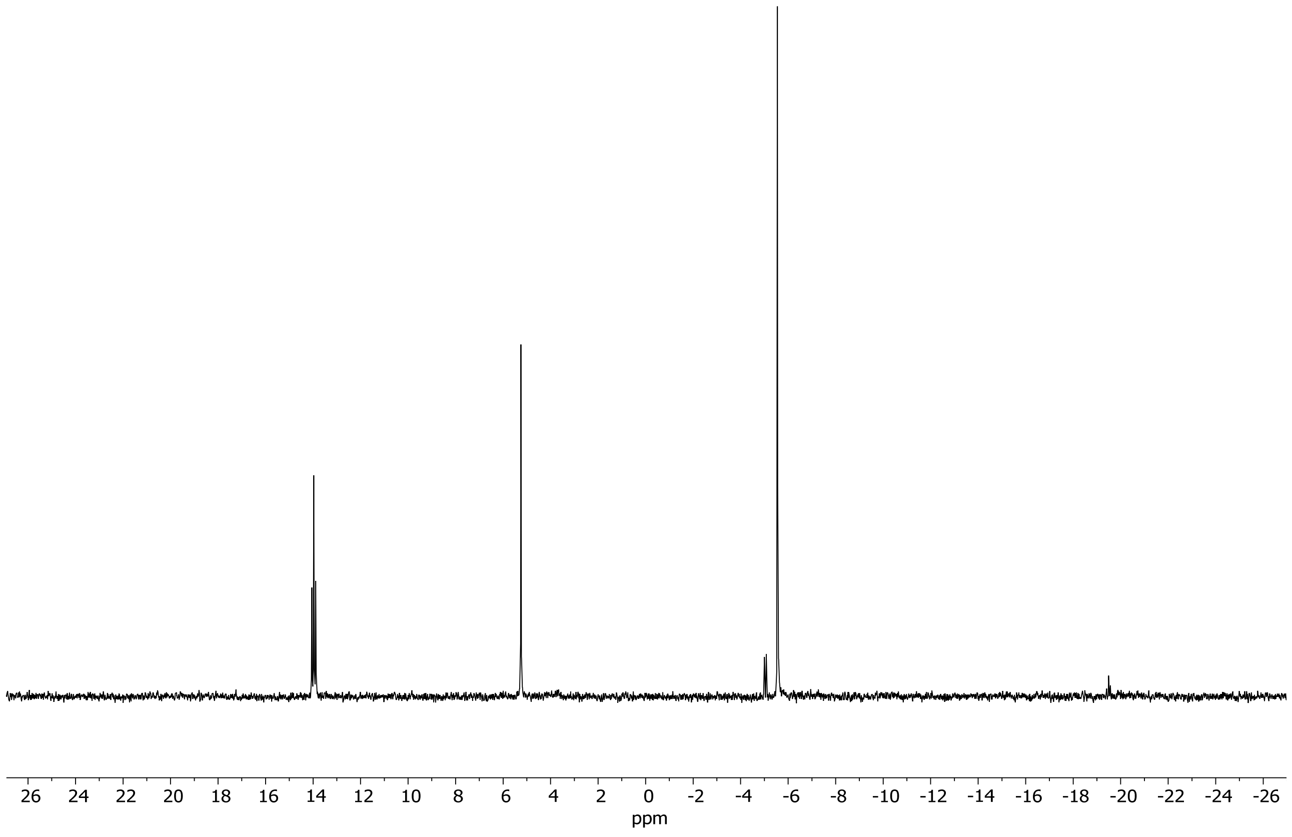

Figure S 35. ^{31}P-NMR (^{1}H-coupled) spectrum of Sample AD-32.

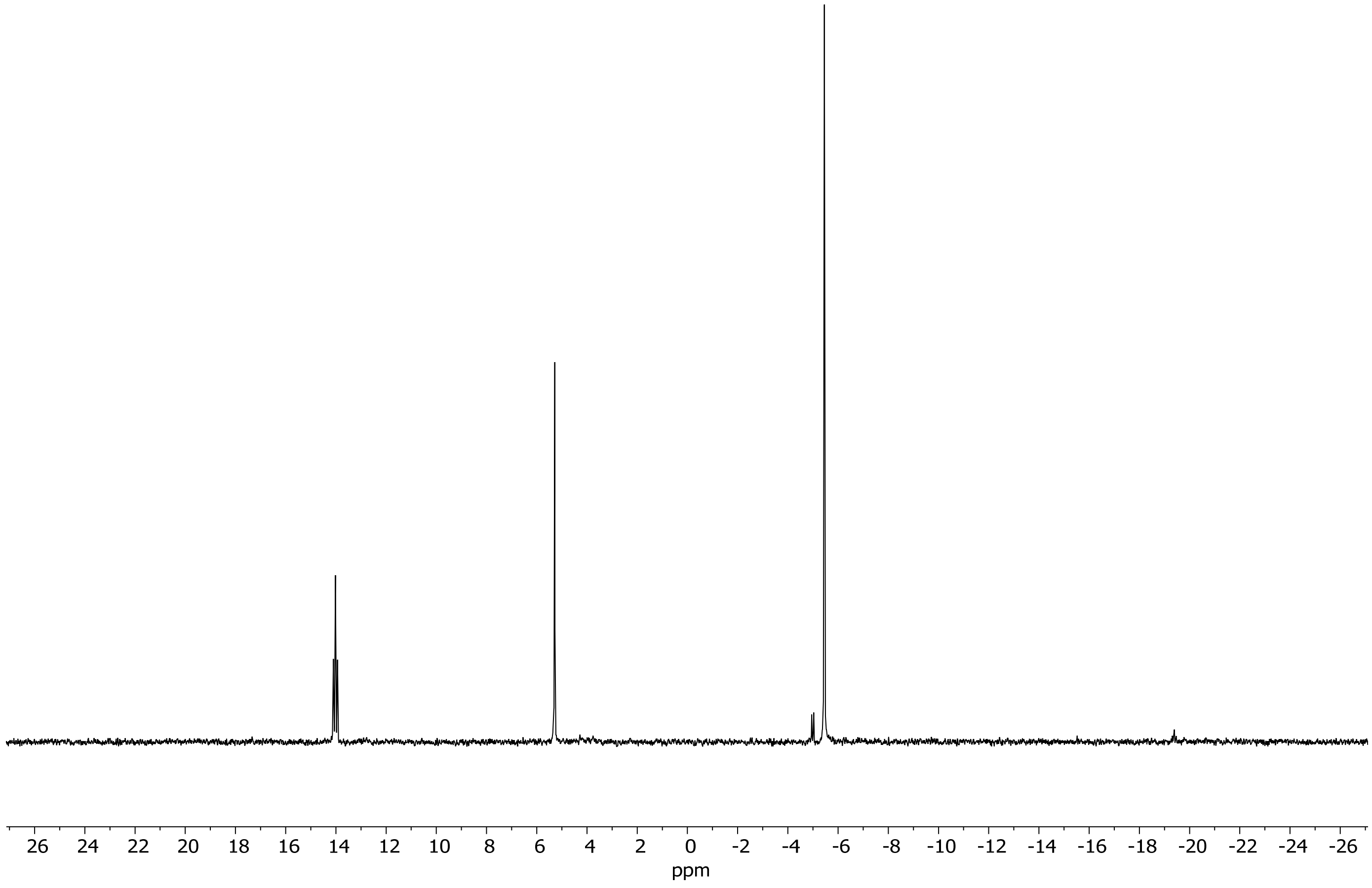

Figure S 36. ^{31}P-NMR (^{1}H-coupled) spectrum of Sample AD-33.

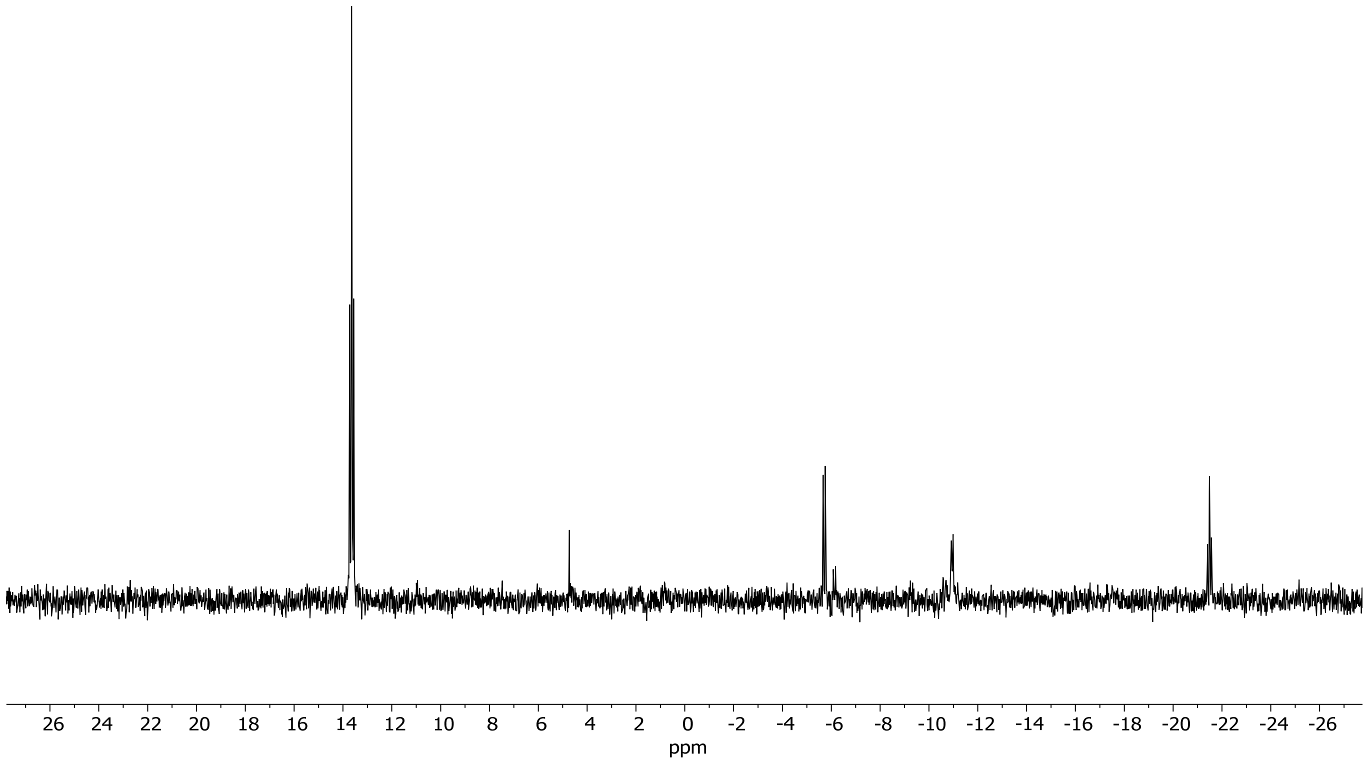

Figure S 37. ^{31}P-NMR (^{1}H-coupled) spectrum of Sample AD-34.

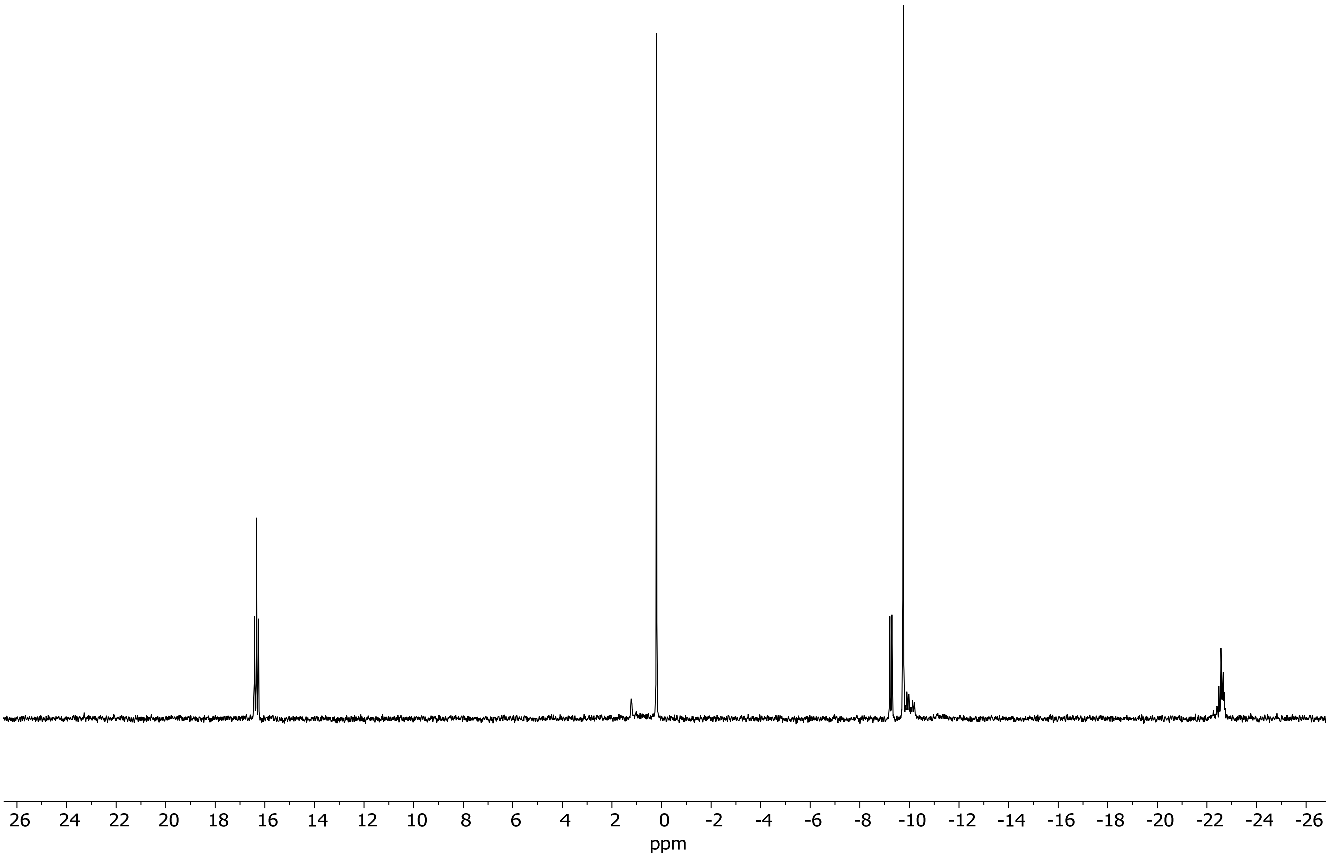

Figure S 38. ^{31}P-NMR (^1H-coupled) spectrum of Sample AD-35.

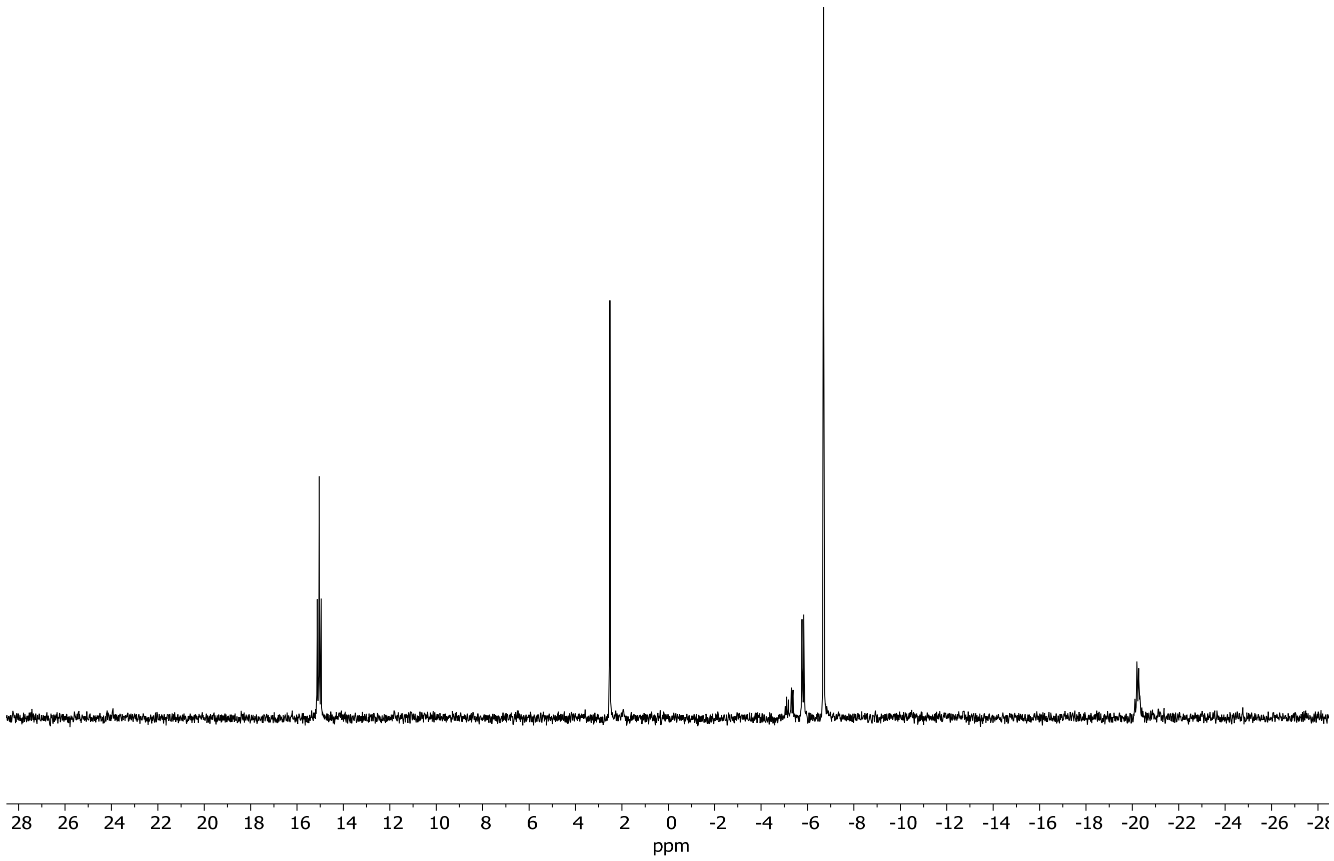

Figure S 39. ^{31}P-NMR (^{1}H-coupled) spectrum of Sample AD-36.

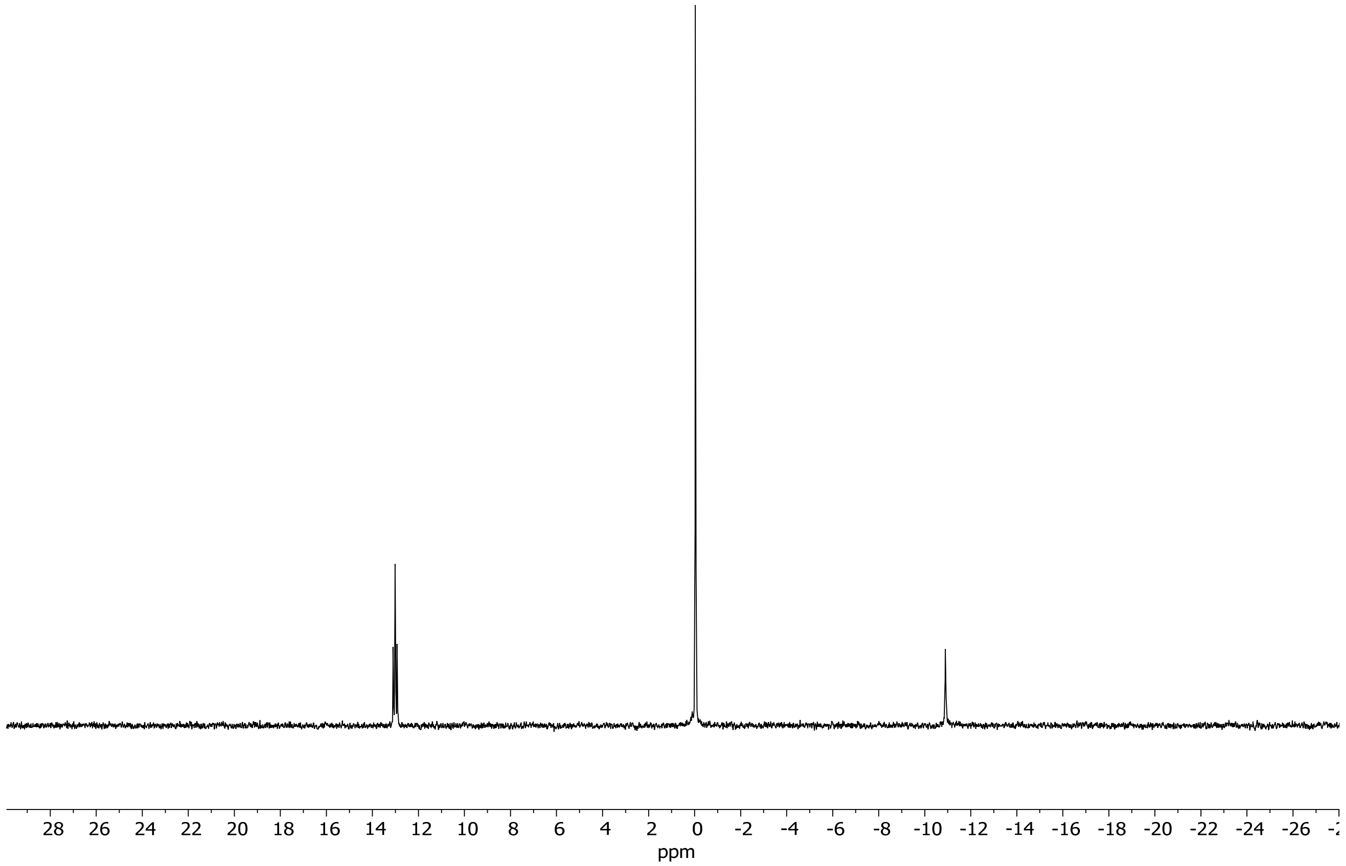

Figure S 40. ^{31}P-NMR (^{1}H-coupled) spectrum of Sample AD-37.

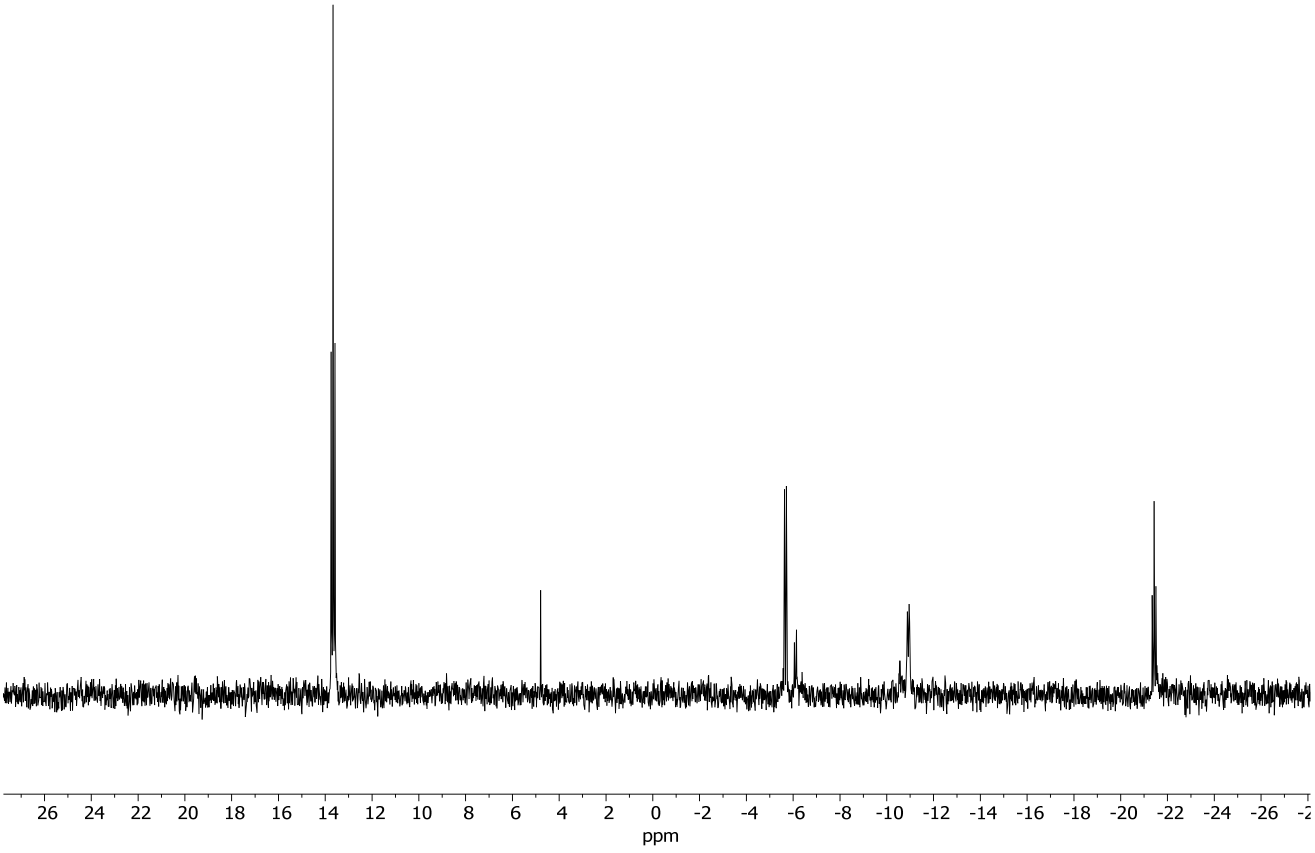

Figure S 41. ^{31}P-NMR (^{1}H-coupled) spectrum of Sample AD-38.

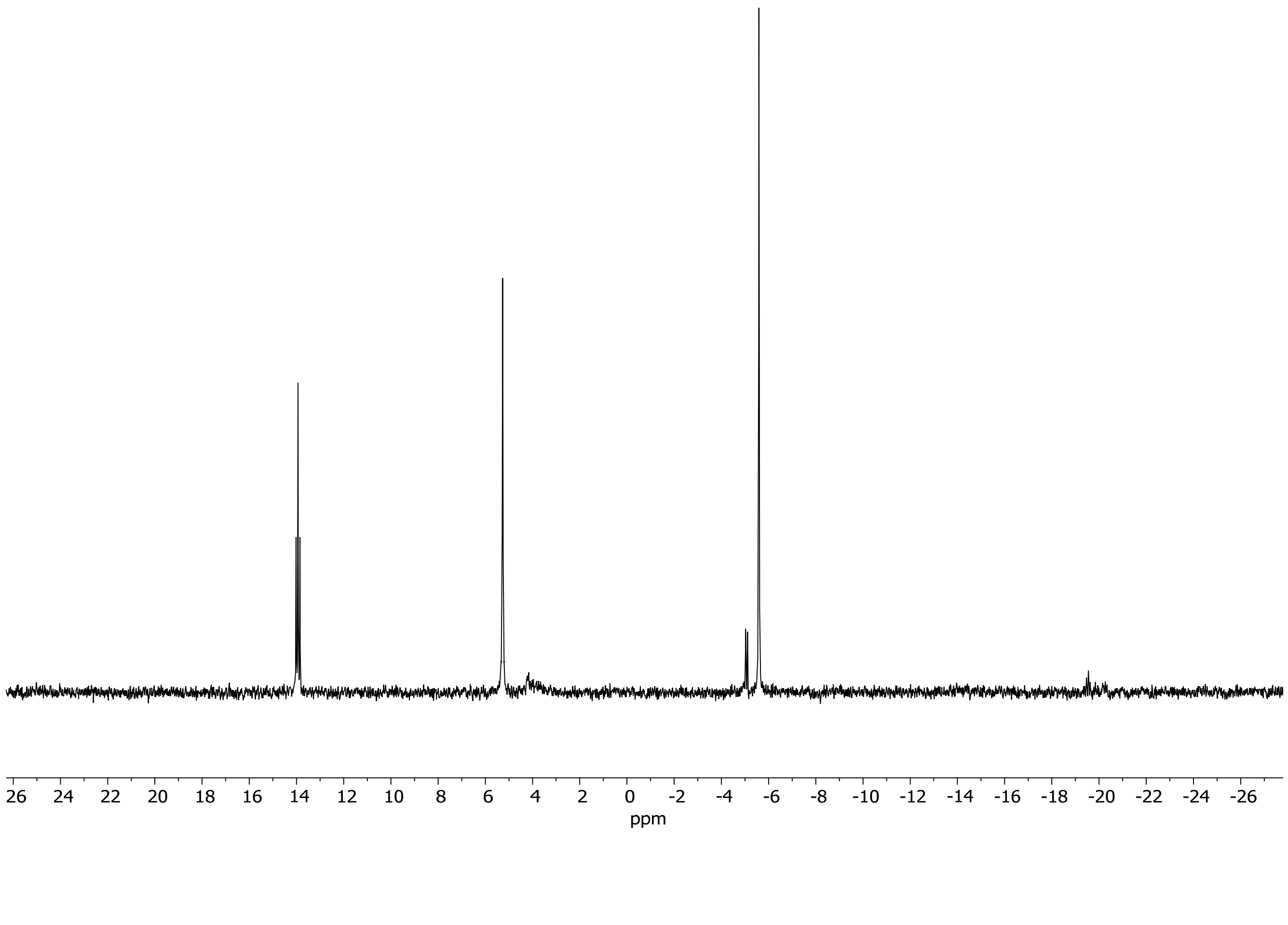

Figure S 42. ^{31}P-NMR (^{1}H-coupled) spectrum of Sample AD-39.

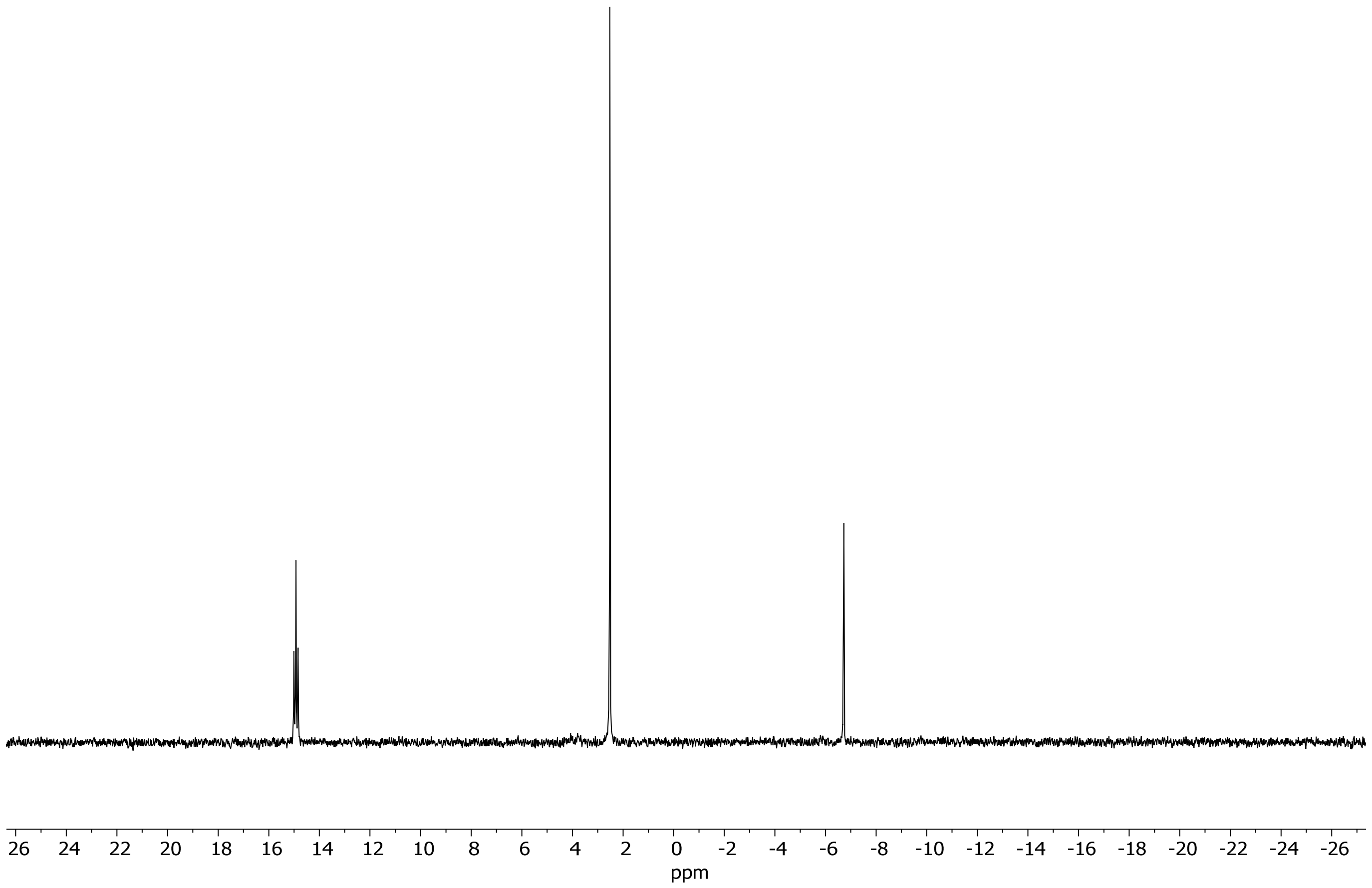

Figure S 43. ^{31}P-NMR (^{1}H-coupled) spectrum of Sample AD-40.

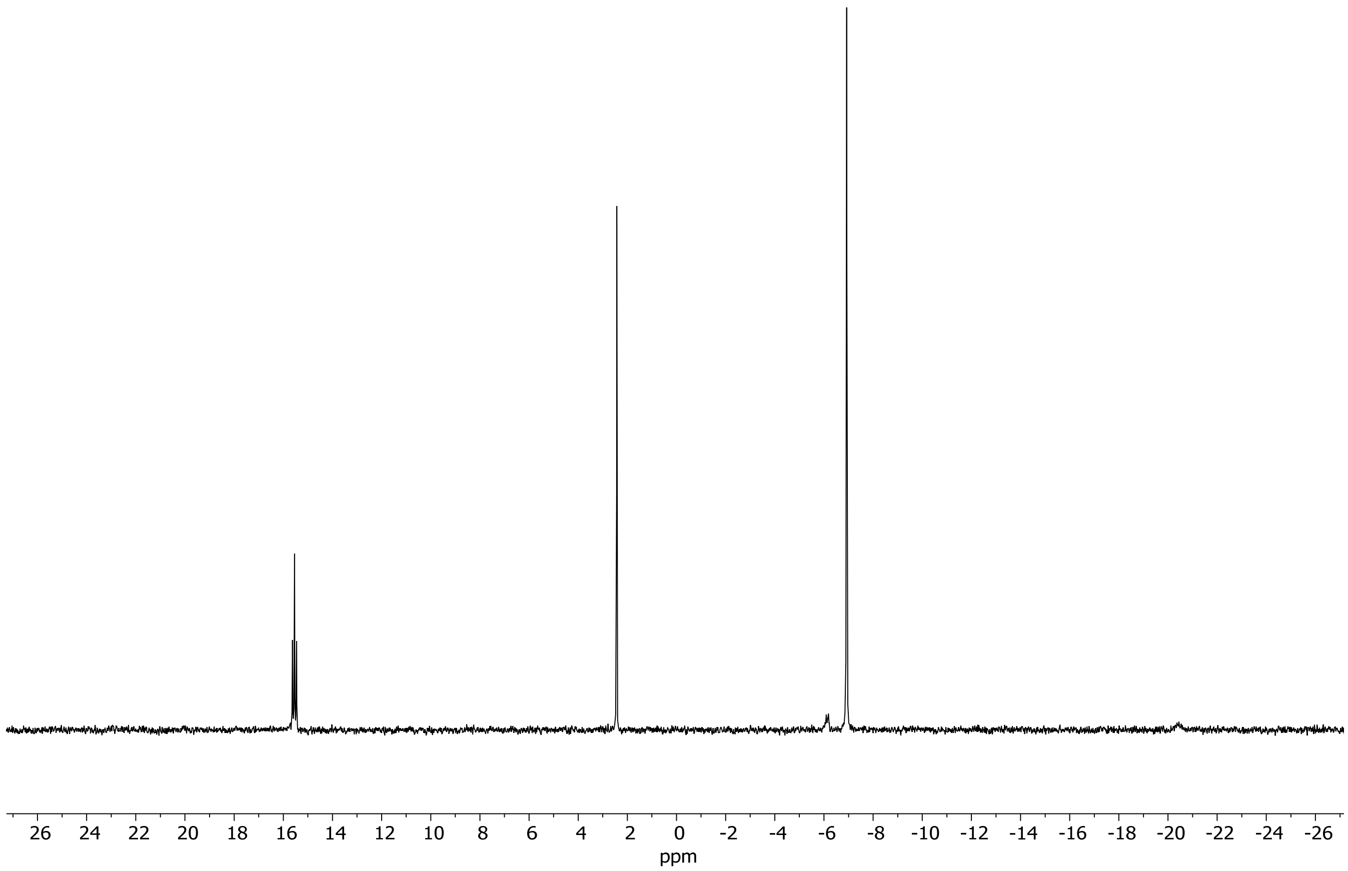

Figure S 44. ^{31}P-NMR (^{1}H-coupled) spectrum of Sample AD-41.

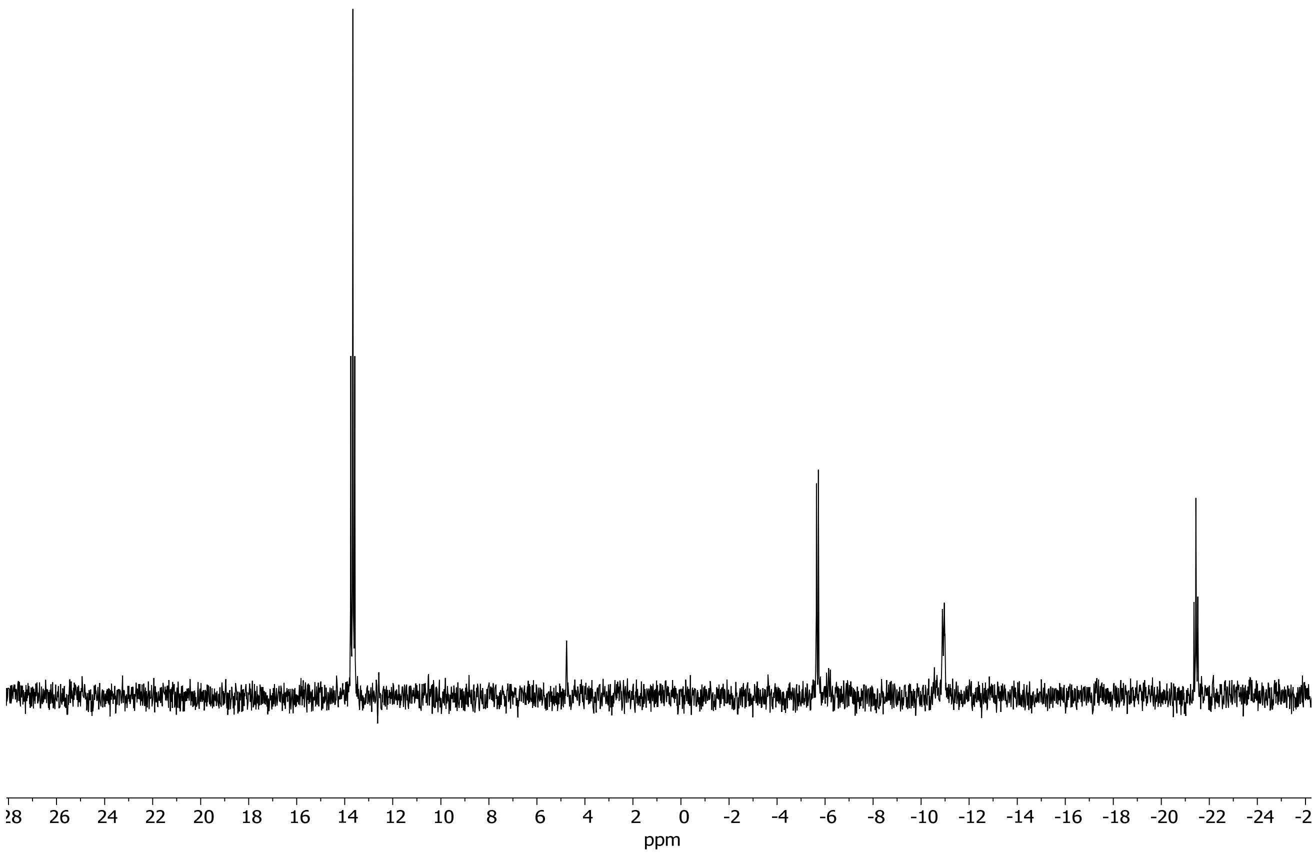
28 26 24 22 20 18 16 14 12 10 8 6 4 2 0 -2 -4 -6 -8 -10 -12 -14 -16 -18 -20 -22 -24 -2
ppm

Figure S 45. ^{31}P-NMR (^{1}H-coupled) spectrum of Sample AD-42.

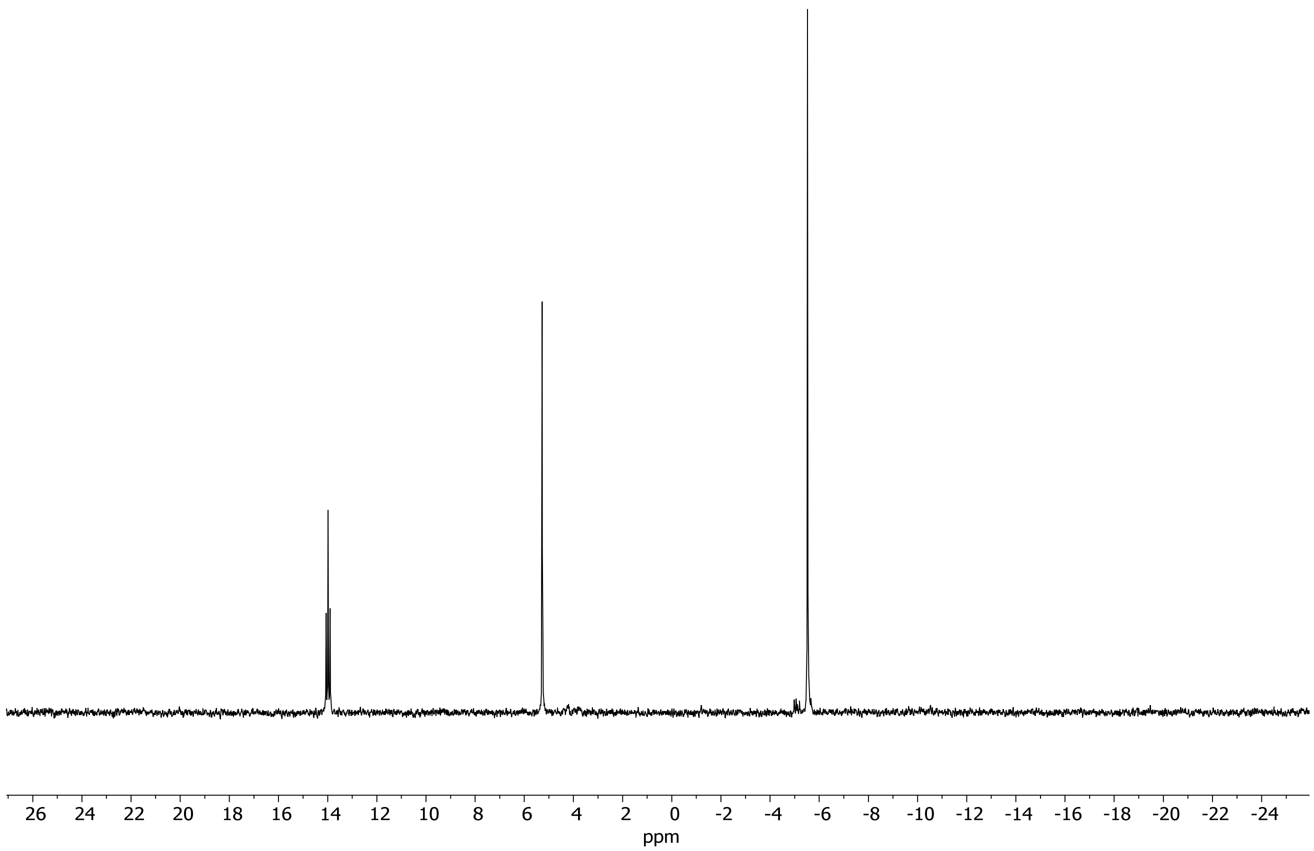

Figure S 46. ^{31}P-NMR (^{1}H-coupled) spectrum of Sample AD-43.

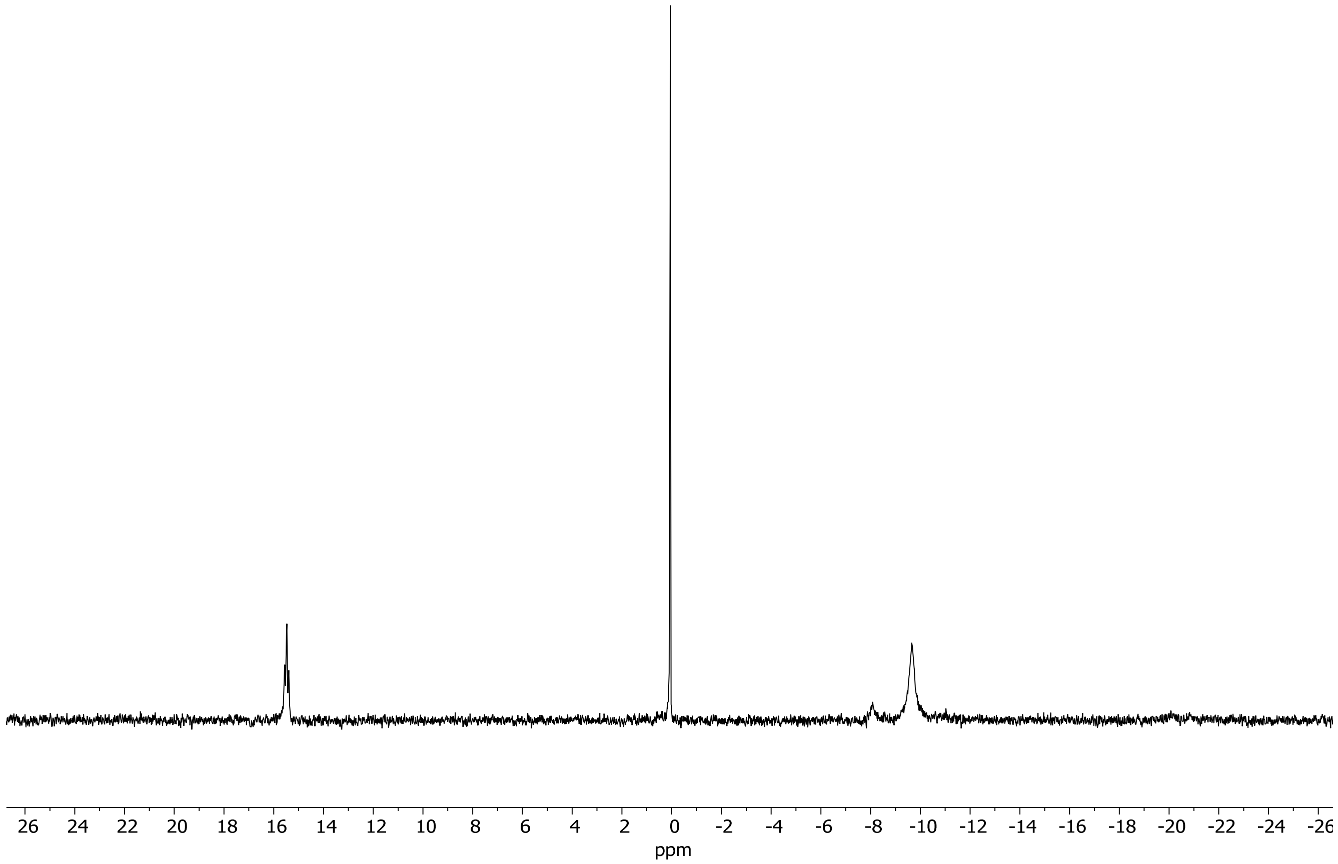

Figure S 47. ^{31}P-NMR (^{1}H-coupled) spectrum of Sample AD-44.

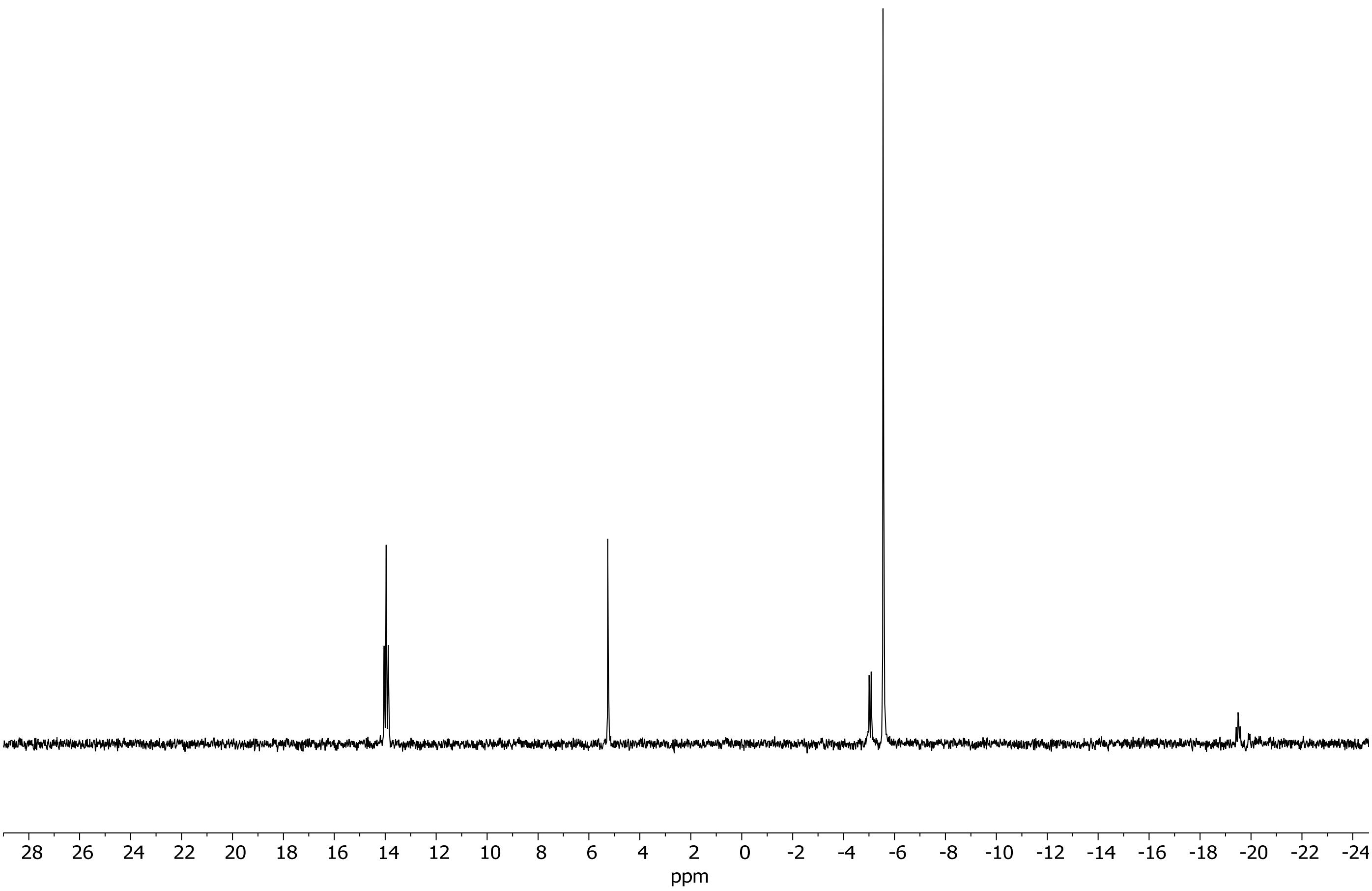

Figure S 48. ^{31}P-NMR (^{1}H-coupled) spectrum of Sample AD-45.

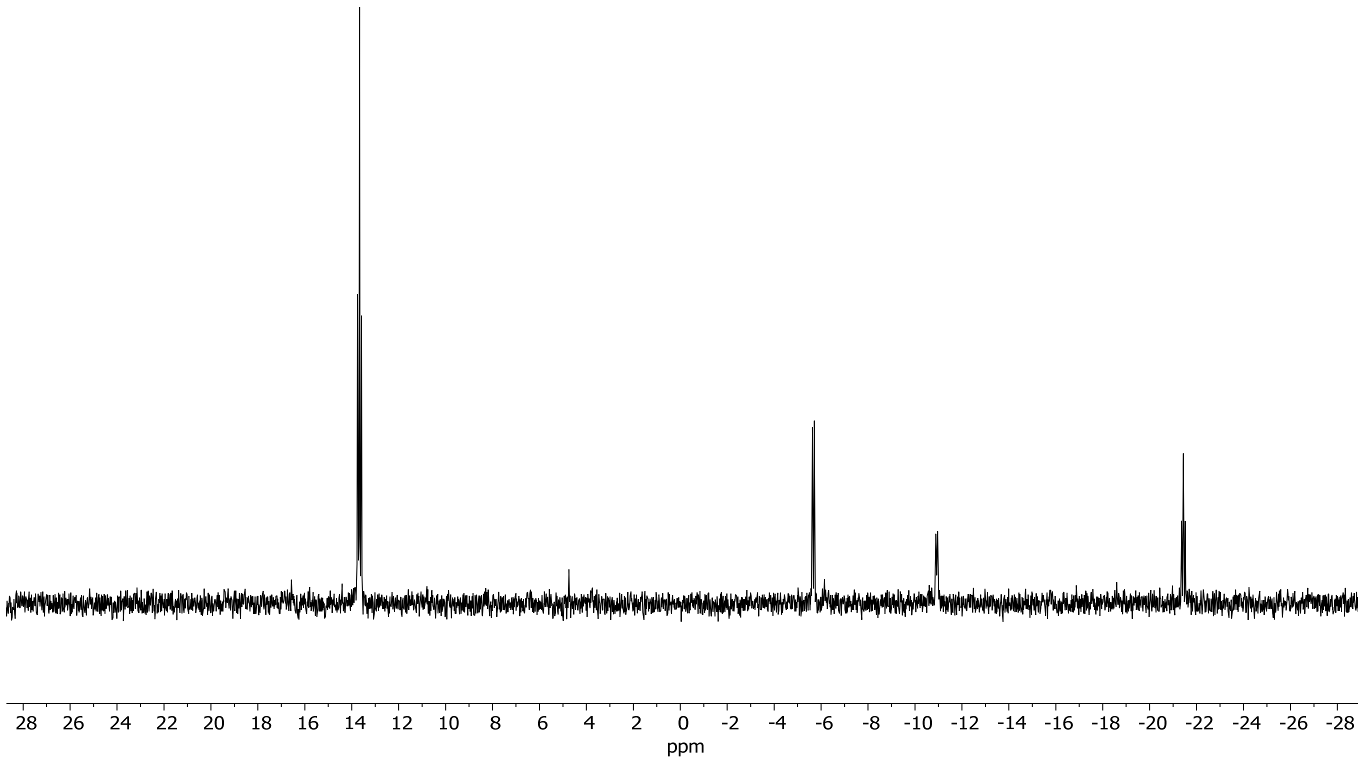

Figure S 49. ^{31}P-NMR (^{1}H-coupled) spectrum of Sample AD-46.

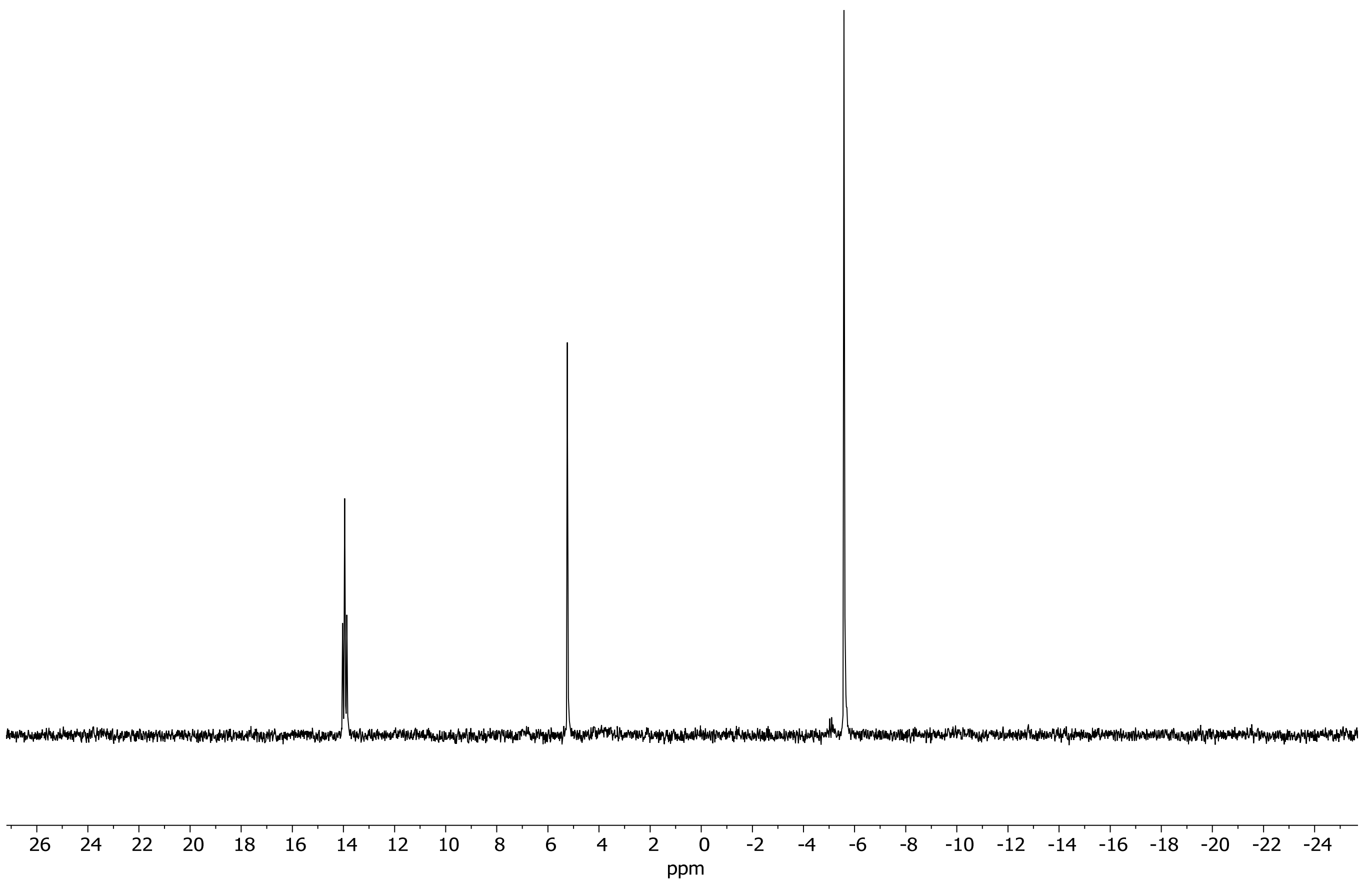

Figure S 50. ^{31}P-NMR (^{1}H-coupled) spectrum of Sample AD-47.

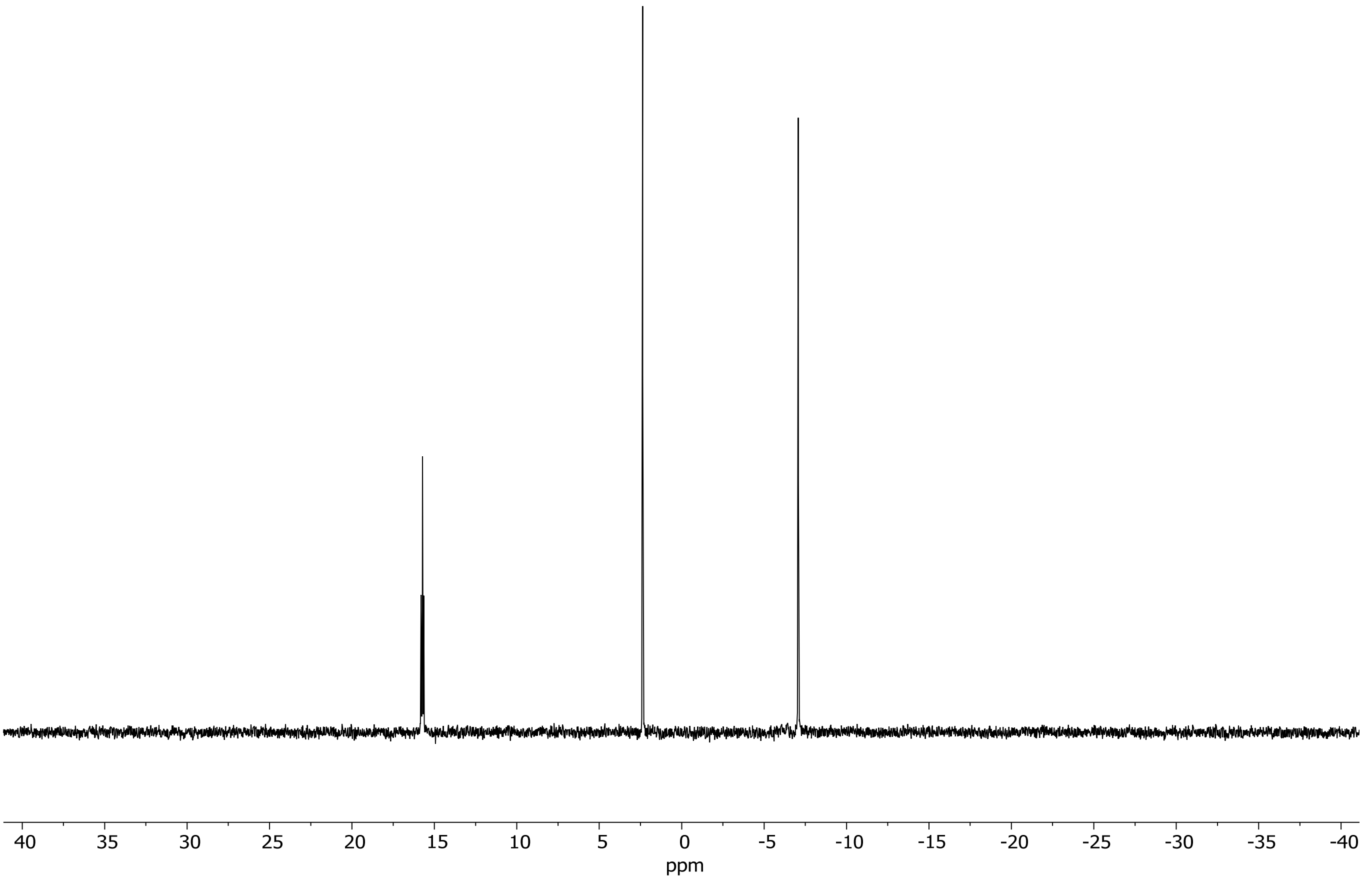

Figure S 51. ^{31}P-NMR (^{1}H-coupled) spectrum of Sample AD-48.

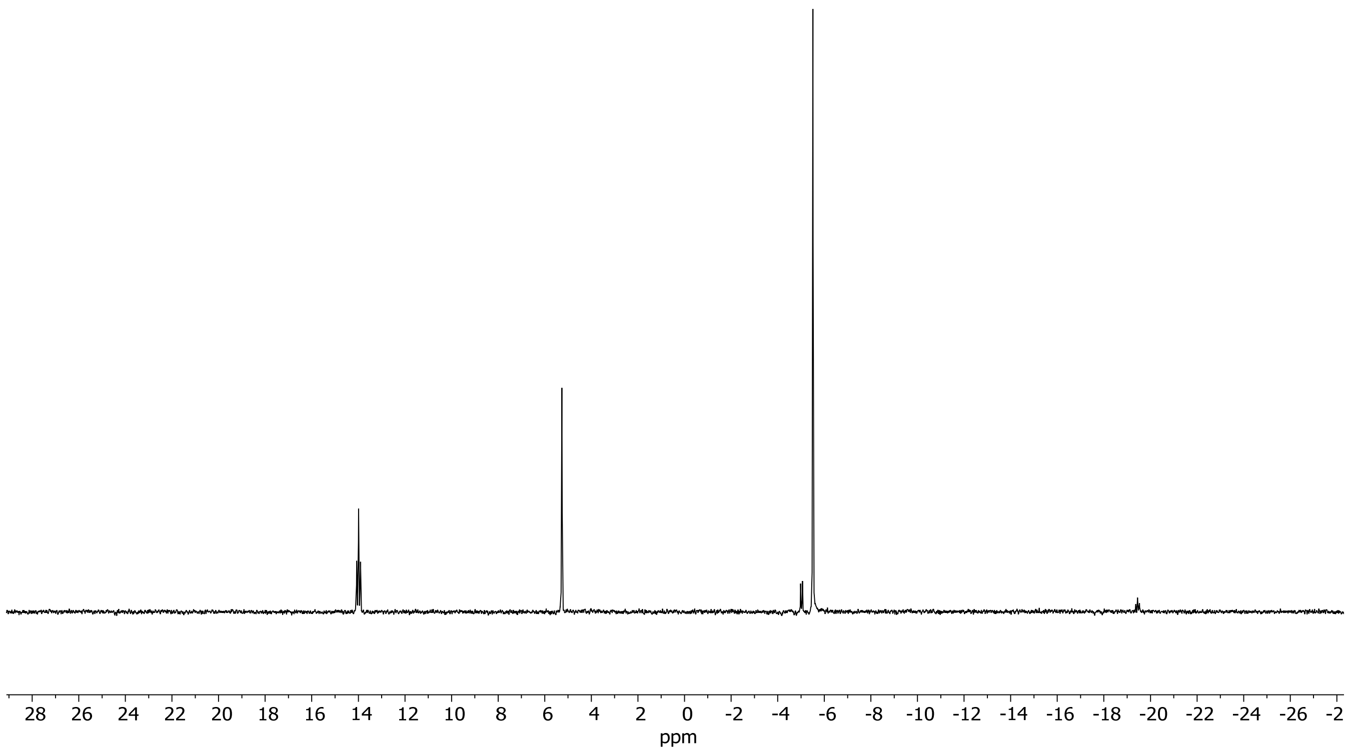

Figure S52 (a)

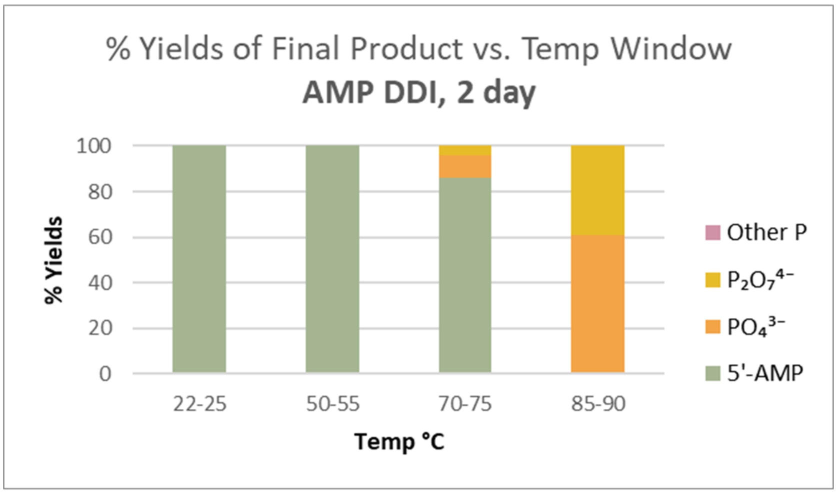
% Yields of Final Product vs. Temp Window
AMP DDI, 2 day
% Yields
100
80
60
40
20
0
22-25
50-55
70-75
85-90
Temp °C
Other P
$P_2O_7^{4-}$
PO_4^{3-}
5'-AMP

(b)

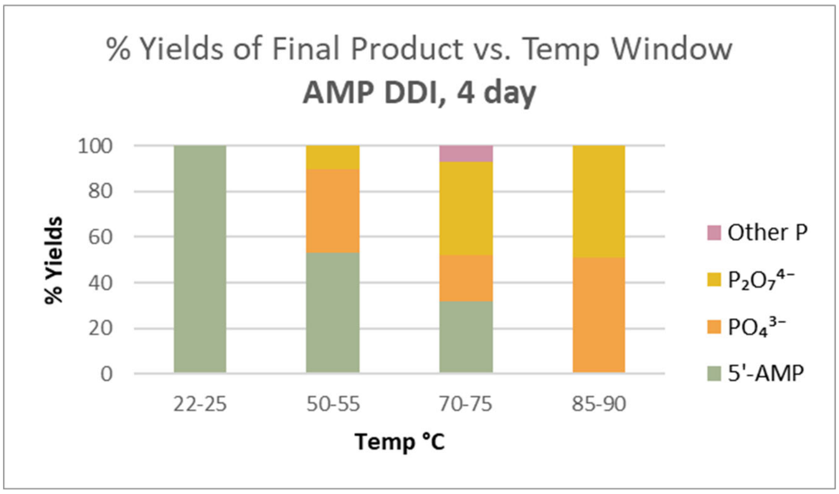
% Yields of Final Product vs. Temp Window
AMP DDI, 4 day
% Yields
100
80
60
40
20
0
22-25
50-55
70-75
85-90
Temp °C
Other P
$P_2O_7^{4-}$
PO_4^{3-}
5'-AMP

(c)

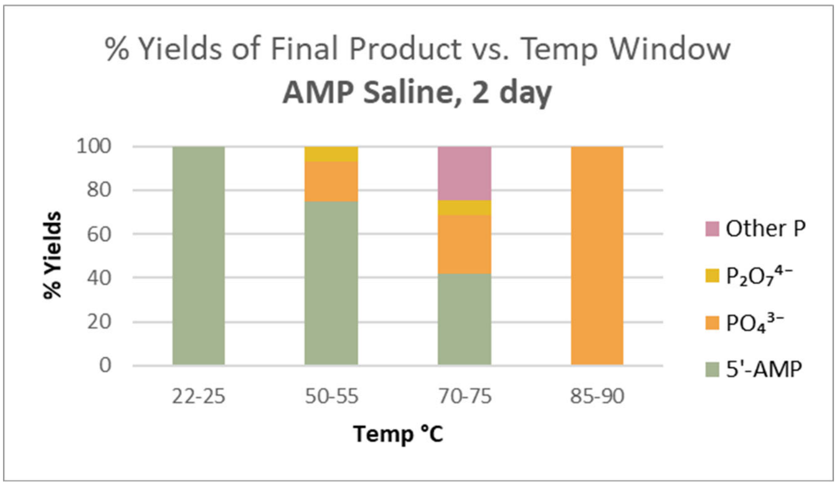
% Yields of Final Product vs. Temp Window
AMP Saline, 2 day
% Yields
100
80
60
40
20
0
22-25
50-55
70-75
85-90
Temp °C
Other P
$P_2O_7^{4-}$
PO_4^{3-}
5'-AMP

(d)

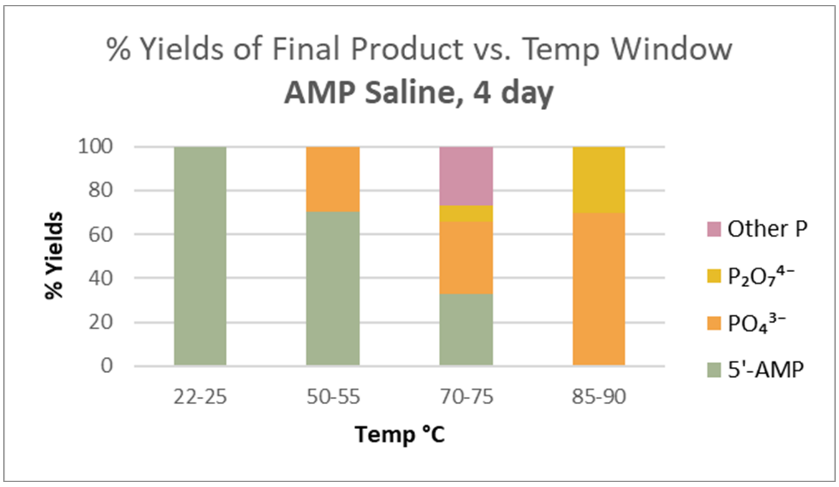
% Yields of Final Product vs. Temp Window
AMP Saline, 4 day
% Yields
100
80
60
40
20
0
22-25
50-55
70-75
85-90
Temp °C
Other P
$P_2O_7^{4-}$
PO_4^{3-}
5'-AMP

(e)

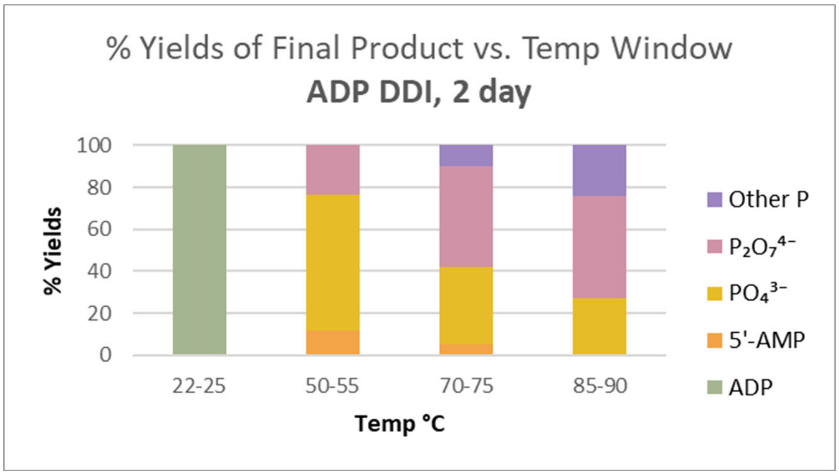
% Yields of Final Product vs. Temp Window
ADP DDI, 2 day
% Yields
100
80
60
40
20
0
22-25
50-55
70-75
85-90
Temp °C
Other P
$P_2O_7^{4-}$
PO_4^{3-}
5'-AMP
ADP

(f)

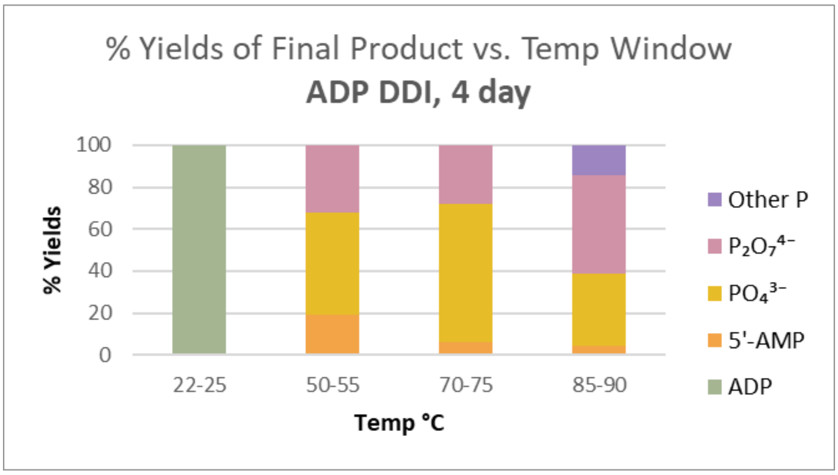
% Yields of Final Product vs. Temp Window
ADP DDI, 4 day
% Yields
100
80
60
40
20
0
22-25
50-55
70-75
85-90
Temp °C
Other P
$P_2O_7^{4-}$
PO_4^{3-}
5'-AMP
ADP

(g)

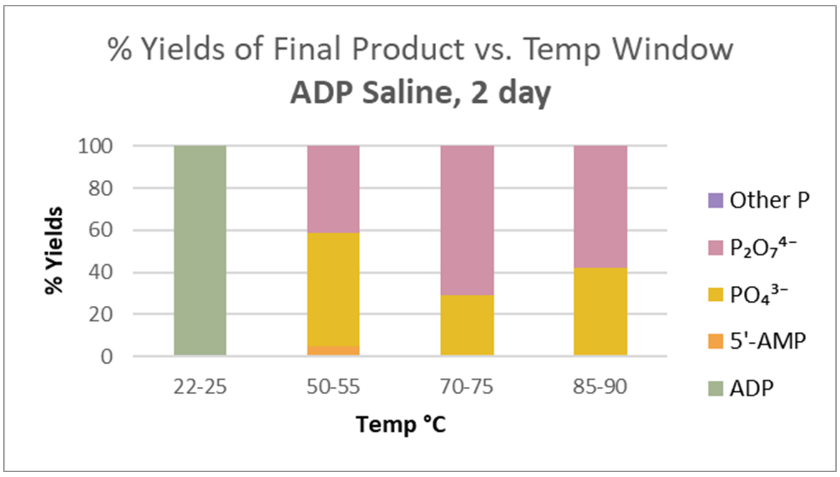

% Yields of Final Product vs. Temp Window
ADP Saline, 2 day
100
80
60
40
20
0
% Yields
22-25
50-55
70-75
85-90
Temp °C
Other P
$P_2O_7^{4-}$
PO_4^{3-}
5'-AMP
ADP

(h)

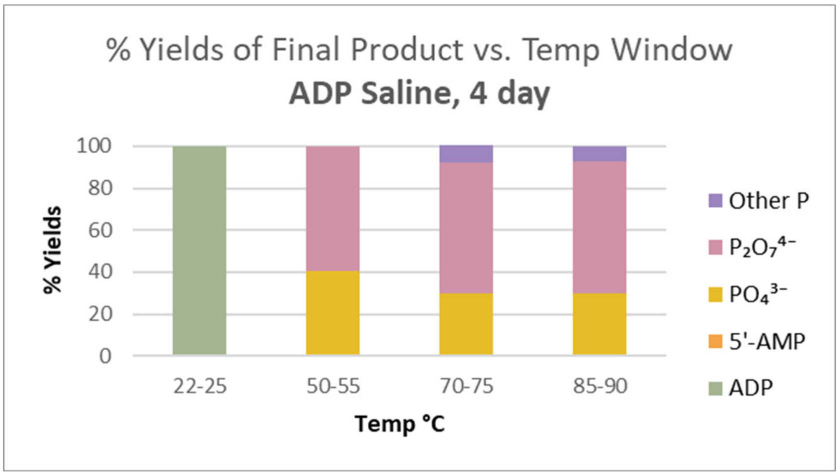

% Yields of Final Product vs. Temp Window
ADP Saline, 4 day
100
80
60
40
20
0
% Yields
22-25
50-55
70-75
85-90
Temp °C
Other P
$P_2O_7^{4-}$
PO_4^{3-}
5'-AMP
ADP

(i)

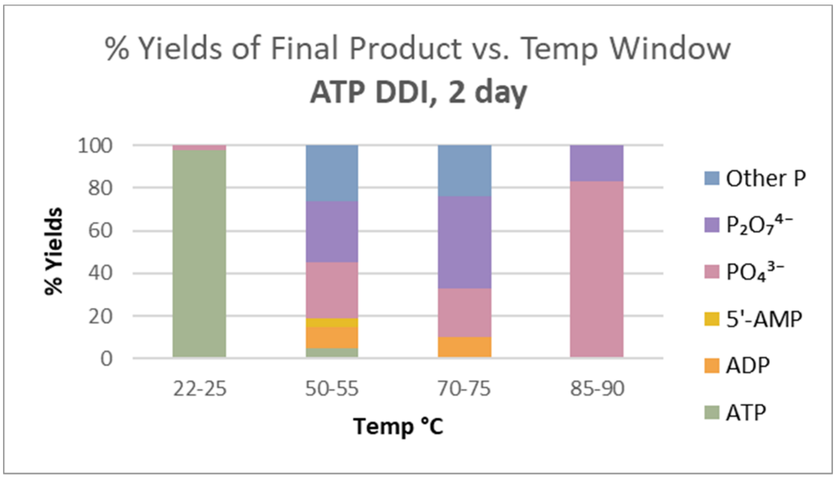

% Yields of Final Product vs. Temp Window
ATP DDI, 2 day
100
80
60
40
20
0
% Yields
22-25
50-55
70-75
85-90
Temp °C
Other P
$P_2O_7^{4-}$
PO_4^{3-}
5'-AMP
ADP
ATP

(j)

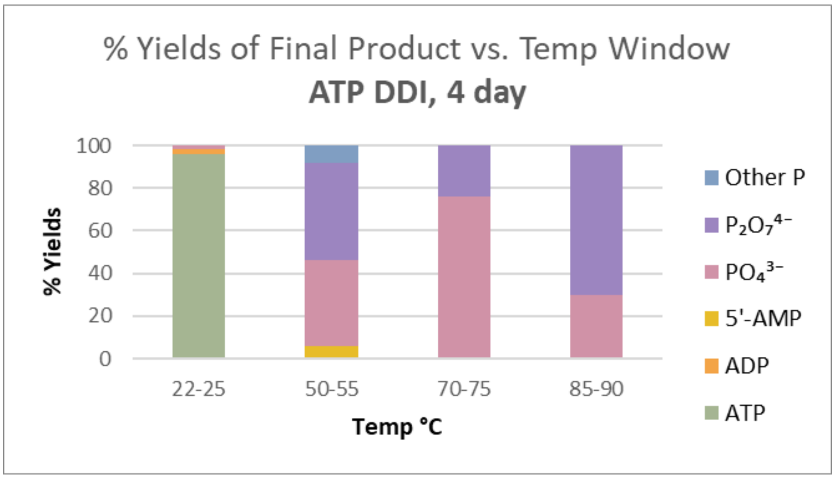
% Yields of Final Product vs. Temp Window
ATP DDI, 4 day
% Yields
100
80
60
40
20
0
22-25
50-55
70-75
85-90
Temp °C
Other P
P₂O₇⁴⁻
PO₄³⁻
5'-AMP
ADP
ATP

(k)

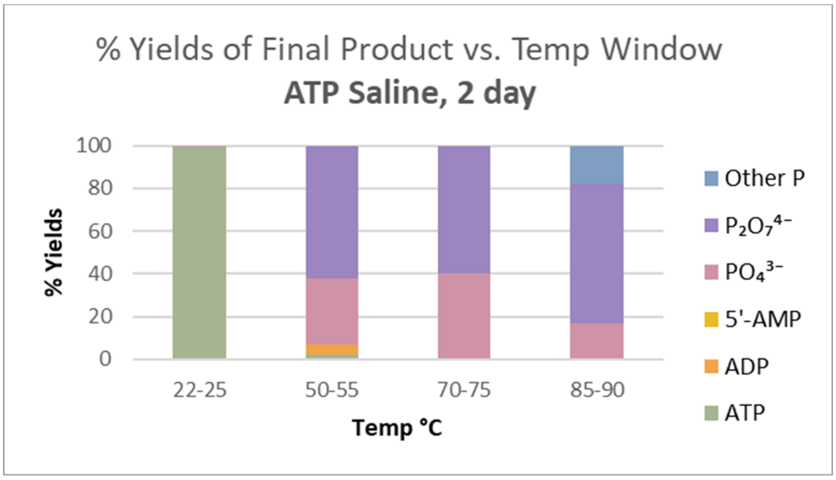
% Yields of Final Product vs. Temp Window
ATP Saline, 2 day
% Yields
100
80
60
40
20
0
22-25
50-55
70-75
85-90
Temp °C
Other P
P₂O₇⁴⁻
PO₄³⁻
5'-AMP
ADP
ATP

(l)

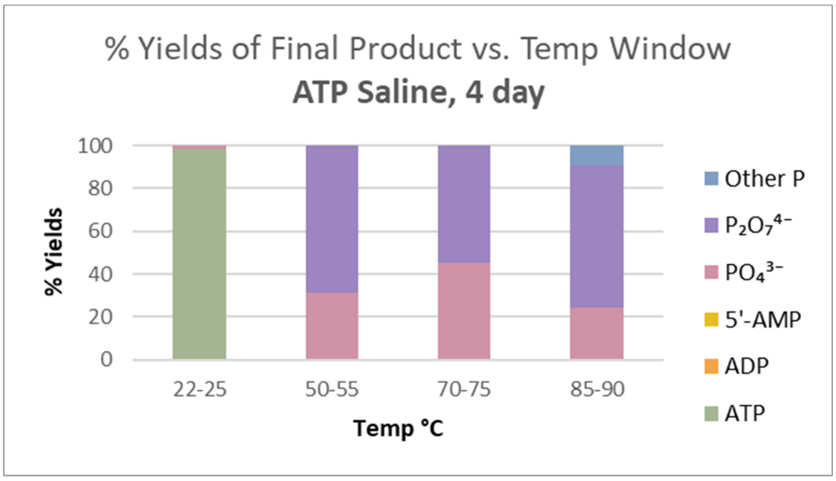
% Yields of Final Product vs. Temp Window
ATP Saline, 4 day
% Yields
100
80
60
40
20
0
22-25
50-55
70-75
85-90
Temp °C
Other P
P₂O₇⁴⁻
PO₄³⁻
5'-AMP
ADP
ATP

II. Phosphorylation reactions with the help of various N-containing additives

AD-CN

(a)

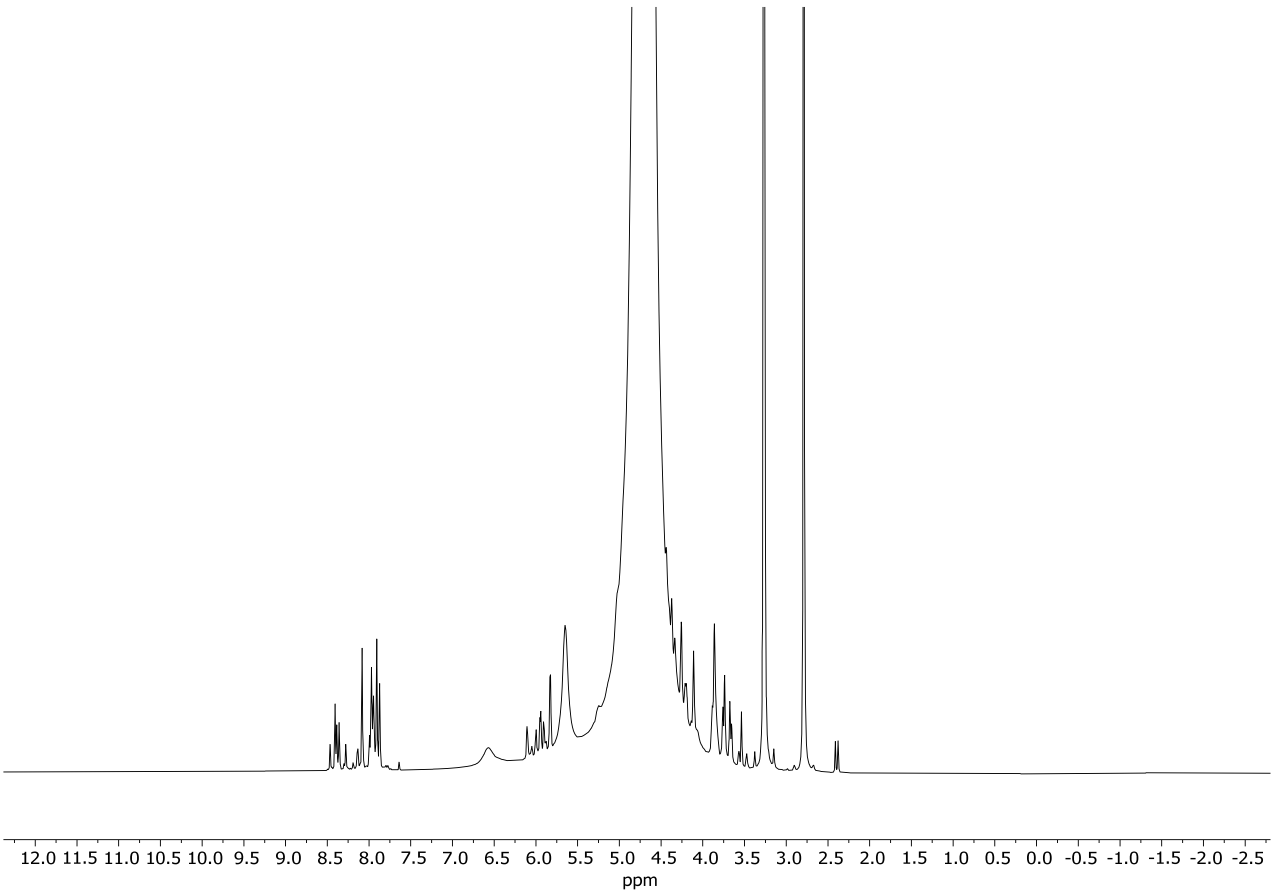

(b)

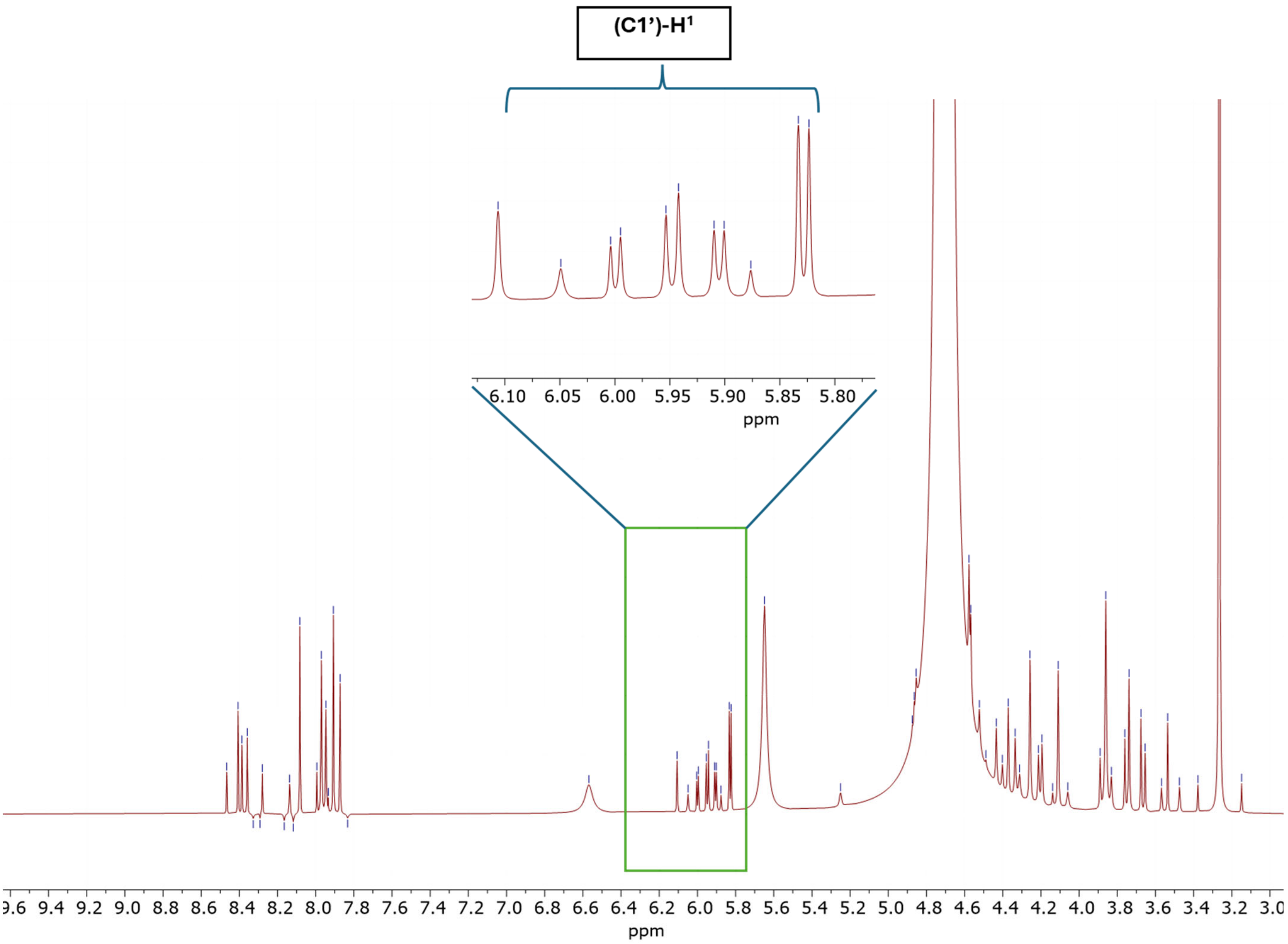
(C1')-H[1]
6.10 6.05 6.00 5.95 5.90 5.85 5.80
ppm
9.6 9.4 9.2 9.0 8.8 8.6 8.4 8.2 8.0 7.8 7.6 7.4 7.2 7.0 6.8 6.6 6.4 6.2 6.0 5.8 5.6 5.4 5.2 5.0 4.8 4.6 4.4 4.2 4.0 3.8 3.6 3.4 3.2 3.0
ppm

(c)

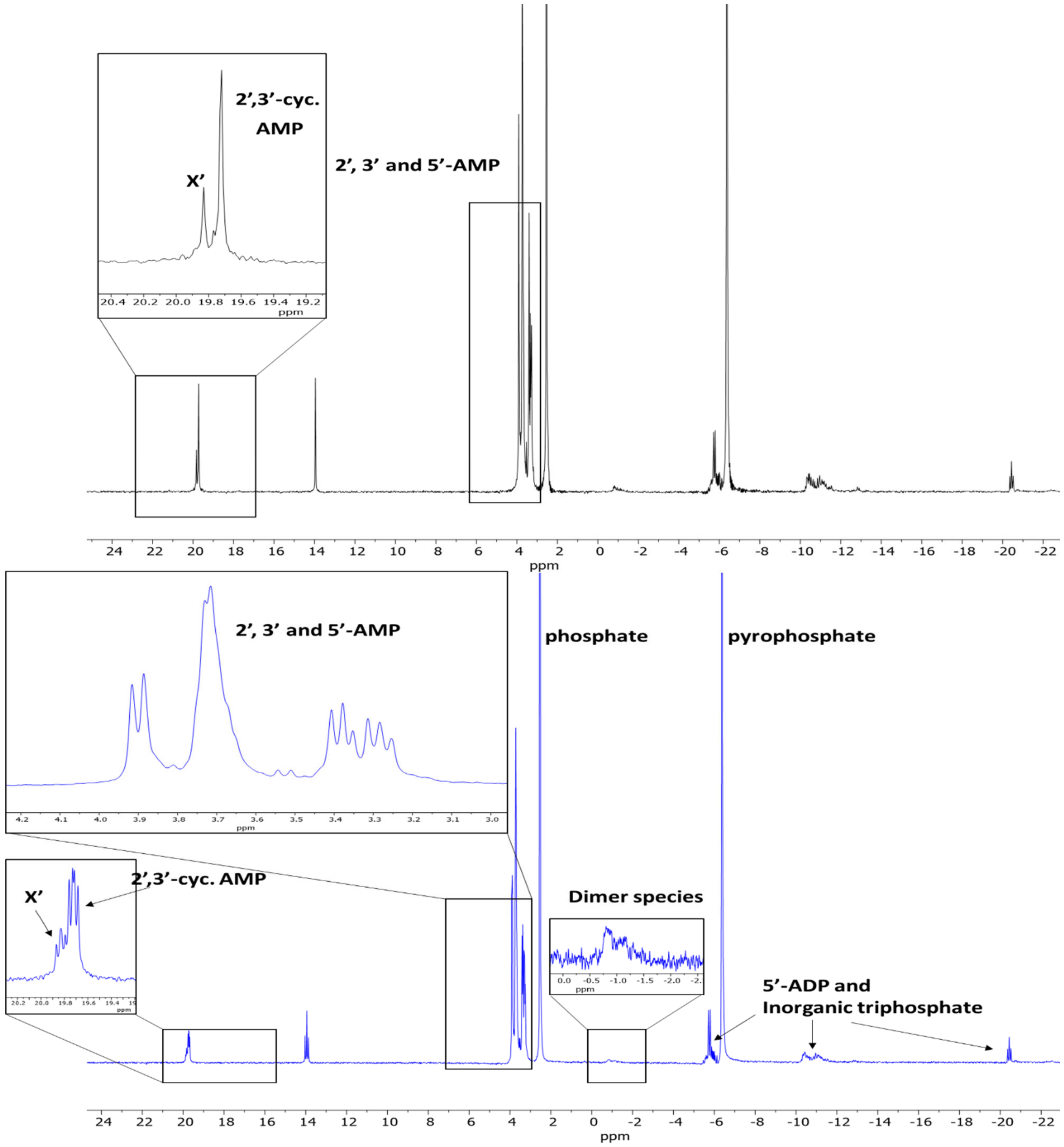

2',3'-cyc. AMP
X'
20.4 20.2 20.0 19.8 19.6 19.4 19.2
ppm
2', 3' and 5'-AMP
24 22 20 18 16 14 12 10 8 6 4 2 0 -2 -4 -6 -8 -10 -12 -14 -16 -18 -20 -22
ppm
2', 3' and 5'-AMP
4.2 4.1 4.0 3.9 3.8 3.7 3.6 3.5 3.4 3.3 3.2 3.1 3.0
ppm
phosphate
pyrophosphate
X'
2',3'-cyc. AMP
Dimer species
5'-ADP and
Inorganic triphosphate
24 22 20 18 16 14 12 10 8 6 4 2 0 -2 -4 -6 -8 -10 -12 -14 -16 -18 -20 -22
ppm

Figure S53. AD-CN. NMR spectra of Sample AD-CN of the phosphorylation of adenosine with ammonium phosphate using cyanamide as additive in water, under evaporative conditions at 65-70 °C. Figure (a): Zoomed part of ^{1}H-NMR spectrum before deconvolution process, (b): zoomed in view of the same figure after deconvolution and top spectrum (a) before the deconvolution process. Both (a) and (b) represent the comparison of the original ^{1}H NMR spectrum with the corresponding deconvolution spectrum and finally (c): ^{31}PNMR both H-coupled and decoupled spectra.

Explanation of Figure S53. AD-CN

In case of ^{31}P-NMR, the blue spectrum is the H-coupled while the black color spectrum is H-decoupled spectrum. In case of H-coupled spectrum from left to right; at 19.8-20.0 ppm a triplet represents 2′, 3′-cyc.AMP while the other smaller triplet (X′) represents a double-phosphorylation product e.g., phosphorylation at 5′. A triplet at 14.0 ppm represents phosphonoacetic acid (PAA) which was used as an internal standard as well as a standard reference. At 3.0-3.9 ppm, doublets represent 2′ and 3′-AMP while a triplet (being overlapped by another peak) would possibly represent 5′-AMP. A tall singlet between 2-3 ppm is the orthophosphate and at 0 to -1 ppm, there are some multiplet representing dimer (adenosine-phosphate-adenosine) molecule and -6 ppm to -22 ppm area represent pyrophosphate (a singlet at -7 ppm), 5′-ADP, and inorganic linear triphosphate (confirmed by a doublet around -6 ppm and a triplet around -22 ppm.

Figure S54 AD-P. 31P-NMR H-decoupled spectrum of Sample AD-P.

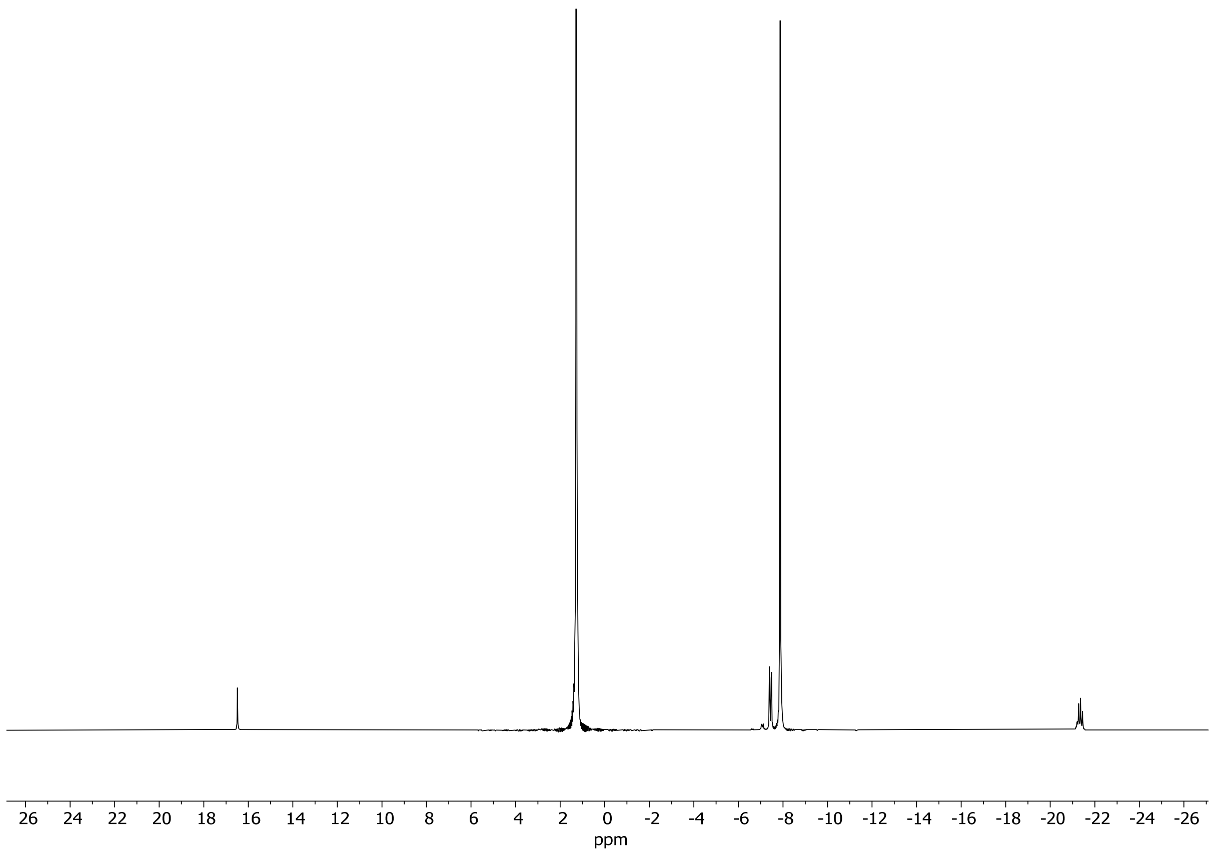

Figure S54 AD-P. ^{31}P-NMR (H-decoupled) NMR spectrum of Sample AD-P of the adenosine phosphorylation by ammonium phosphate without any additive in water, under evaporative conditions at 65-70 °C. This reaction suggests no prominent C-O-P bonds.

Figure S55 MS-1

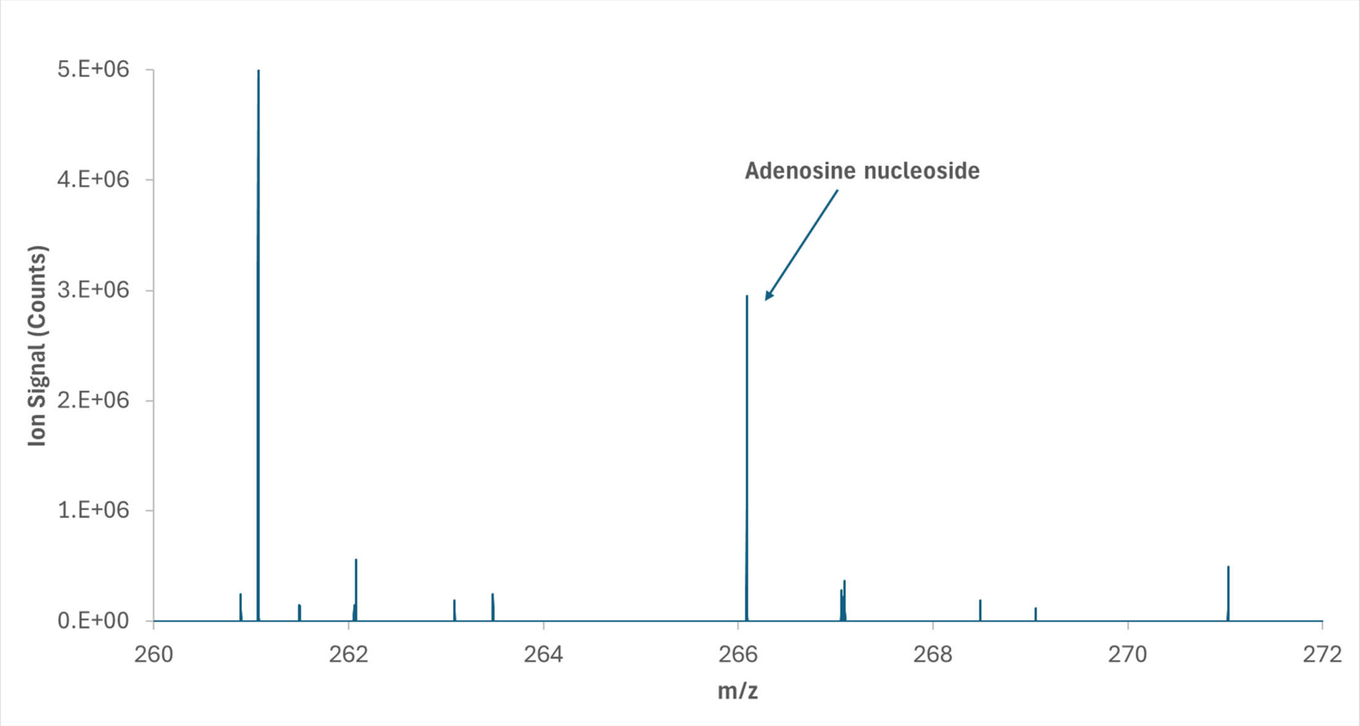

Figure S55 MS-1. Mass spectrometry (direct injection, negative mode) of adenosine-P sample, the corresponding peak at m/z 266 corresponding to the unreacted adenosine nucleoside.

Figure S56 MS-2

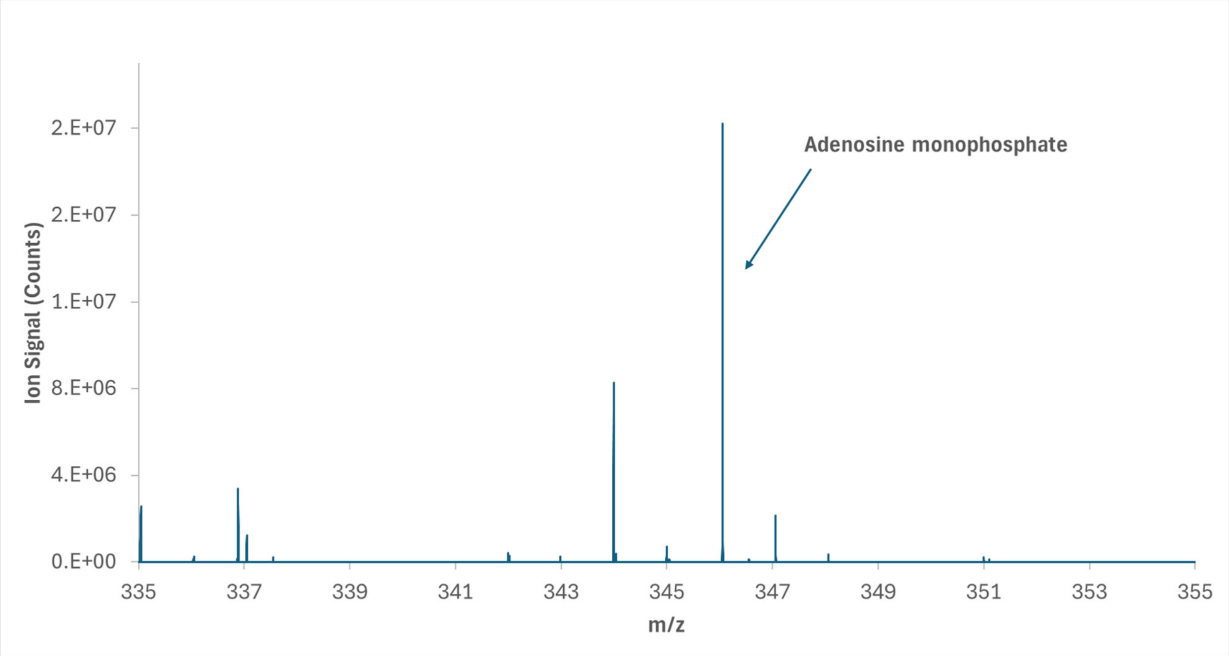

Figure S56 MS-2. Mass spectrometry (direct injection, negative mode) of adenosine-P sample, the corresponding peak at m/z 346 corresponding to AMP species (including 2',3' and 5'-AMPs).

Figure S57 MS-3

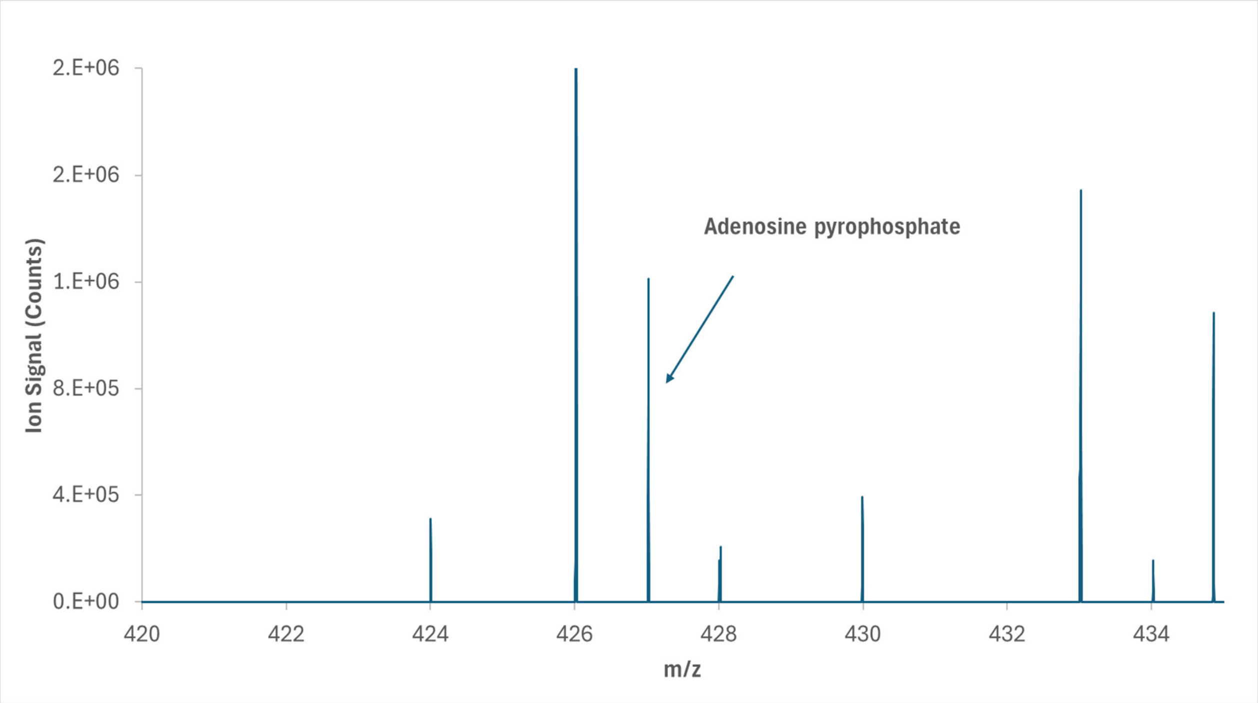

Figure S57 MS-3. Mass spectrometry (direct injection, negative mode) of adenosine-P sample, the corresponding peak at m/z 426 corresponding to ADP.

Figure S58 MS-4

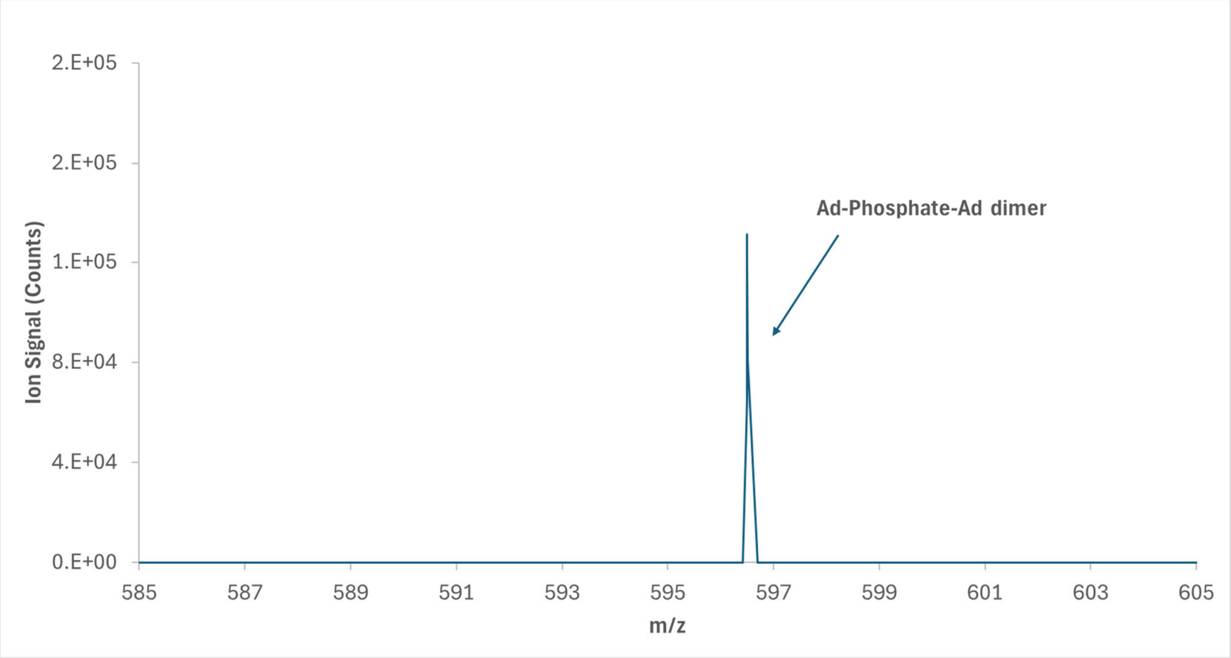

Figure S58 MS-4. Mass spectrometry (direct injection, negative mode) of adenosine-P sample, the corresponding peak at m/z 595 corresponding to adenosine-phosphate-adenosine dimer molecule.

References

1. Pasek et. al. Serpentinization as a route to liberating phosphorus on habitable worlds. Geochim. Cosmochim. Acta., 336, 2022, 332-340.
2. Source downloaded from; https://www.aiinmr.com/wp-content/uploads/2020/06/31P-of-Adenosine-Phoshates.pdf on July 22nd, 2025.
